# CDK6 Levels Regulate Quiescence Exit in Human Hematopoietic Stem Cells

**DOI:** 10.1016/j.stem.2015.01.017

**Published:** 2015-03-05

**Authors:** Elisa Laurenti, Catherine Frelin, Stephanie Xie, Robin Ferrari, Cyrille F. Dunant, Sasan Zandi, Andrea Neumann, Ian Plumb, Sergei Doulatov, Jing Chen, Craig April, Jian-Bing Fan, Norman Iscove, John E. Dick

**Affiliations:** 1 Princess Margaret Cancer Centre, University Health Network, Toronto, ON M5G 1L7, Canada; 2 Department of Molecular Genetics, University of Toronto, Toronto, ON M5S 1A8, Canada; 3 Ecole Normale Supérieure de Cachan, Département de Biologie, Cachan, 94235, France; 4 Ecole Polytechnique Fédérale de Lausanne, LMC, Station 12, Lausanne, CH-1015, Switzerland; 5 Division of Pediatric Hematology/Oncology, Boston Children’s Hospital and Harvard Medical School, Harvard Stem Cell Institute, Boston, MA 02115, USA; 6 Illumina, San Diego, CA 92121, USA; 7 Department of Medical Biophysics, University of Toronto, Toronto, ON M5G 1L7, Canada

## Abstract

Regulated blood production is achieved through the hierarchical organization of dormant hematopoietic stem cell (HSC) subsets that differ in self-renewal potential and division frequency, with long-term (LT)-HSCs dividing the least. The molecular mechanisms underlying this variability in HSC division kinetics are unknown. We report here that quiescence exit kinetics are differentially regulated within human HSC subsets through the expression level of CDK6. LT-HSCs lack CDK6 protein. Short-term (ST)-HSCs are also quiescent but contain high CDK6 protein levels that permit rapid cell cycle entry upon mitogenic stimulation. Enforced CDK6 expression in LT-HSCs shortens quiescence exit and confers competitive advantage without impacting function. Computational modeling suggests that this independent control of quiescence exit kinetics inherently limits LT-HSC divisions and preserves the HSC pool to ensure lifelong hematopoiesis. Thus, differential expression of CDK6 underlies heterogeneity in stem cell quiescence states that functionally regulates this highly regenerative system.

## Highlights

• Human long-term (LT) and short-term (ST) HSCs are equally quiescent
• LT- and ST-HSCs differ in division kinetics and expression of CDK6
• CDK6 expression regulates the timing of exit from quiescence
• Differential regulation of quiescence helps maintain hematopoiesis

## Graphical Abstract

**Figure:**
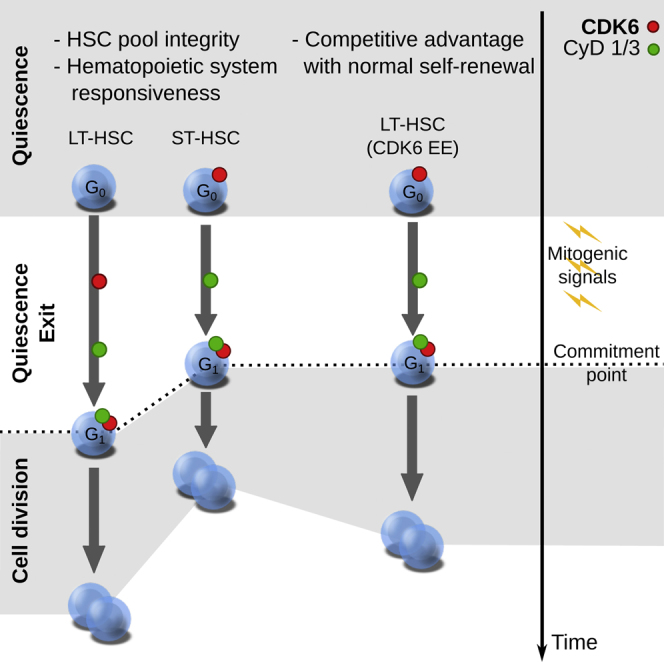

## Teaser

The hematopoietic stem cell (HSC) compartment is heterogeneous in terms of cell cycle properties. Laurenti et al. show that the timing of exit from quiescence in human HSC subsets is controlled by CDK6 expression levels. This differential control has an impact on the long-term preservation of the HSC pool.

## Introduction

Hematopoiesis ensures that blood demand is met under homeostatic and stress conditions through tightly controlled regulation of hematopoietic stem cells (HSCs) and their progeny. HSCs are historically identified by the unique capacity to self-renew, providing long-term, serial reconstitution of the entire hematopoietic system upon their transplantation into myeloablated hosts. Functional self-renewal of HSCs is associated with reduced cell cycle activity. Seminal papers demonstrated that cell cycle becomes more frequent as HSCs gradually differentiate into lineage-restricted progenitors (Bradford et al., 1997; Morrison and Weissman, 1994; Pietrzyk et al., 1985; Suda et al., 1983; Uchida et al., 2003). Although the HSC compartment was thought to be heterogeneous in cycling ability (Micklem and Ogden, 1976) 40 years ago, this has only recently been supported by experimental evidence as follows. (1) Label retaining studies (Foudi et al., 2009; Qiu et al., 2014; Takizawa et al., 2011; Wilson et al., 2008) conclusively established that the HSC pool comprises at least two compartments differing in their frequency of division. (2) The most dormant cells have the highest repopulation capacity and can be reversibly brought into cell cycle through extrinsic cues, especially upon injury (Foudi et al., 2009; Wilson et al., 2008). (3) The HSC pool has been fractionated into long-term (LT-), intermediate-term (IT-), short-term (ST-) HSCs and multipotent progenitors (MPPs) and is hierarchically organized based on progressively reduced repopulation capacity and increased cycling properties (Benveniste et al., 2010; Cheshier et al., 1999; Copley et al., 2012; Foudi et al., 2009; Oguro et al., 2013; Passegué et al., 2005; Qiu et al., 2014; Wilson et al., 2008). While the hierarchically organized HSC subsets are widely thought to prevent HSCs exhaustion and preserve lifelong blood production, knowledge of the molecular mechanisms that govern the variable cycling properties of each HSC subset is lacking.

Quiescence, defined as a reversible absence of cycling, also called G_0_, is a defining feature of HSCs first described in Lajtha (1963). Most transgenic and knockout mouse models altering HSC function decrease quiescence, leading to HSC exhaustion (reviewed in Pietras et al., 2011; Rossi et al., 2012). Quiescence and infrequent cycling of HSCs are considered to protect against damage accumulation, and impaired maintenance of HSC quiescence is thought to contribute to aging and leukemia. However, understanding how HSCs switch from quiescence to cycling and how division, self-renewal, and differentiation are integrated is lacking.

Upon reception of mitogenic signals, multiple processes must occur: HSCs must exit quiescence to enter the cell cycle, which then must be traversed to complete a division. This requires reactivating all the necessary metabolic and cell cycle machinery. Doubling time analysis at homeostasis has shown that ST-HSCs and MPPs divide more frequently than LT-HSCs (Foudi et al., 2009; Oguro et al., 2013; Wilson et al., 2008). Little is known about quiescence exit. It is unclear if and how it is differentially regulated among distinct HSC subsets and if the duration of this exit affects HSC function. We recently showed that the duration of a division starting from G_0_ after stimulation by a mitogenic signal is shorter in IT-HSCs than in LT-HSCs (Benveniste et al., 2010). The unknown mechanism underlying increased cycling in IT/ST-HSCs could theoretically be due to (1) easier activation from external stimuli, (2) less time in G_0_, (3) faster exit from quiescence, (4) faster completion of divisions, or (5) a combination of these. An integrated view is necessary to ascertain how these properties in HSC subsets are molecularly regulated. Here, we establish that the duration of HSC exit from quiescence upon mitogenic stimulation is differentially regulated within the human HSC pool by a CDK6-primed quiescence state in ST-HSCs. Tight control of quiescence exit length via CDK6 levels plays an important role in HSC pool dynamics, preserving integrity and preventing LT-HSCs clonal expansion.

## Results

### Heterogeneity in the Human HSC Pool

**Figure 1:**
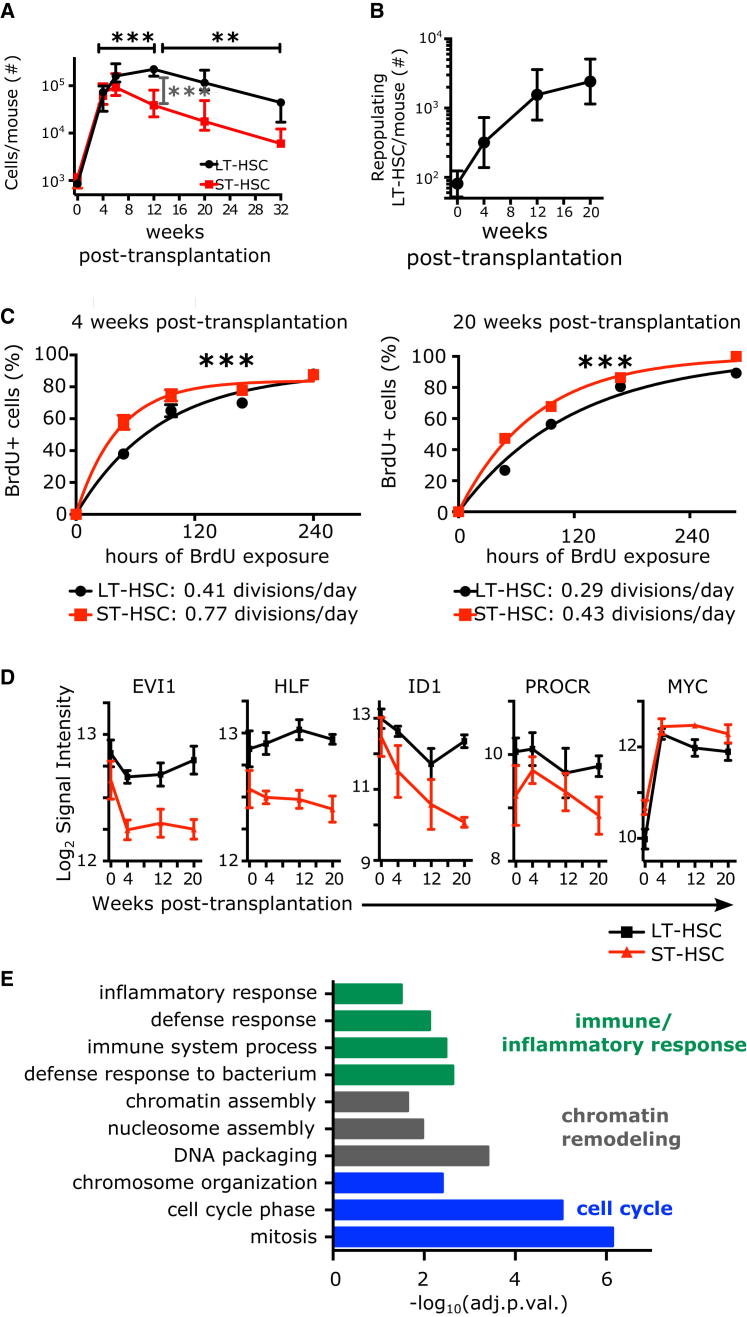
Human HSC Subsets in the Xenograft Divide with Distinct Frequencies and Display Distinct Transcriptional Profiles (A) Number of cells per mouse of indicated populations in the bone marrow of the mice at indicated time points post-transplantation of 70,000 Lin^−^ CB (saturating number of LT-HSCs). Median and interquantile ranges are shown. ^∗∗∗^p < 0.01 by one-way ANOVA and Tukey test. (B) The number of repopulating LT-HSCs per mouse at indicated time points post-transplantation were calculated by multiplying the number of phenotypic LT-HSCs shown in (A) by the frequency of long-term repopulating cells indicated in Table S1. (C) BrdU incorporation kinetics over 12 days of LT-HSC (black) and ST-HSC (red) enriched populations isolated from pools of two to five mice engrafted with 70,000 Lin^−^ CB cells. BrdU was started either at 4 (left panel, expanding phase) or 20 weeks post-transplantation (right panel, equilibrium phase). n = 1–4 pools of three to five mice from six (4 weeks) or one (20 weeks) independent CB samples. Curve is least-squares fit. Left panel: R^2^ > 0.96; right panel: R^2^ > 0.98. Doubling times (half times of fit) in hours are shown in the insert. ^∗∗∗^p < 0.01 by extra-sum of squares test. (D and E) Derivation of a 241-gene signature distinguishing LT- and ST-HSCs in unperturbed CB over 20 weeks in a xenotransplant. (D) Examples of five expression profiles of genes with known HSC function over the course of 20 weeks of xenotransplant (black: LT-HSCs, red: ST-HSCs), mean ± S.E.M shown, n = 3 per time point. (E) Selected gene ontology terms significantly enriched in the 241-gene LT-HSC/ST-HSC core signature. Shown is the −log_10_ of the Benjamini-Hochberg adjusted p value. See also Figure S1.

The cycling properties of mouse HSC subpopulations are described, but they have not been validated in the human HSC hierarchy. Human LT-HSCs, isolated from umbilical cord blood (CB) as Lin^−^ CD34^+^ CD38^−^ CD45RA^−^ CD90^+^ CD49f^+^ (Notta et al., 2011), provide robust multilineage repopulation beyond 30 weeks in the NSG mouse xenograft assay with about 10% frequency (Notta et al., 2011) and efficiently engraft upon secondary transplantation (Table S1). In contrast, Lin^−^ CD34^+^ CD38^−^ CD45RA^−^ CD90^−^ CD49f^−^ cells generate multilineage grafts over intermediate time periods (Notta et al., 2011), but they lack serial transplantation ability and thus have limited self-renewal (Table S1). According to the criteria used in mouse, this population corresponds to ST-HSCs. Importantly, LT- and ST-HSC-enriched populations can be purified with the cell surface markers indicated above from NSG mice repopulated with human cells (Table S1). Similar to transplantation models in mice, phenotypic human LT- and ST-HSCs expand in the first 4 weeks after xenotransplant (with >50% that actively cycle) then regain quiescence by 20 weeks when a transient equilibrium phase is reached (Figures 1A, S1A, and S1B). In our model, functionally repopulating LT-HSCs expanded ≈30-fold in 20 weeks (Figure 1B).

To estimate the division frequency of human HSC subsets, we tracked BrdU incorporation kinetics of phenotypic LT- and ST-HSCs in xenografts and observed, similarly to mouse models, that phenotypic LT-HSCs divide less frequently (1.5- to 1.9-fold) than phenotypic ST-HSCs (Figure 1C), whether in expansion or at equilibrium. Although both subsets highly proliferate after transplantation, we detected fewer ST-HSCs, possibly because of their higher drive to produce differentiated cells (Figure 1A). To capture a core signature of genes distinguishing LT- from ST-HSCs, we subjected LT- and ST-HSCs isolated from CB and at different times after xenotransplantation to transcriptome analysis. Using the Bayesian Estimation of Temporal Regulation algorithm (Aryee et al., 2009), we identified 241 genes showing sustained differential expression between LT- and ST-HSCs independent of environmental effects and changes in proliferation (Table S2 and Figures S1C–S1E). This was significantly more than in our previous static analyses of CB (Laurenti et al., 2013; Notta et al., 2011). This signature contains genes important in murine HSC function (Gazit et al., 2013; Jankovic et al., 2007; Kataoka et al., 2011; Laurenti et al., 2008) (Figure 1D) and is enriched for gene ontology terms related to the immune/inflammatory response, chromatin remodeling, and most significantly, cell cycle regulation (Figure 1E). Thus, human ST-HSCs, similar to mouse, have lower repopulation capacity, more frequent divisions, and a distinct transcriptional profile compared to LT-HSCs.

### LT-HSCs and ST-HSCs Are Equally Quiescent

**Figure 2:**
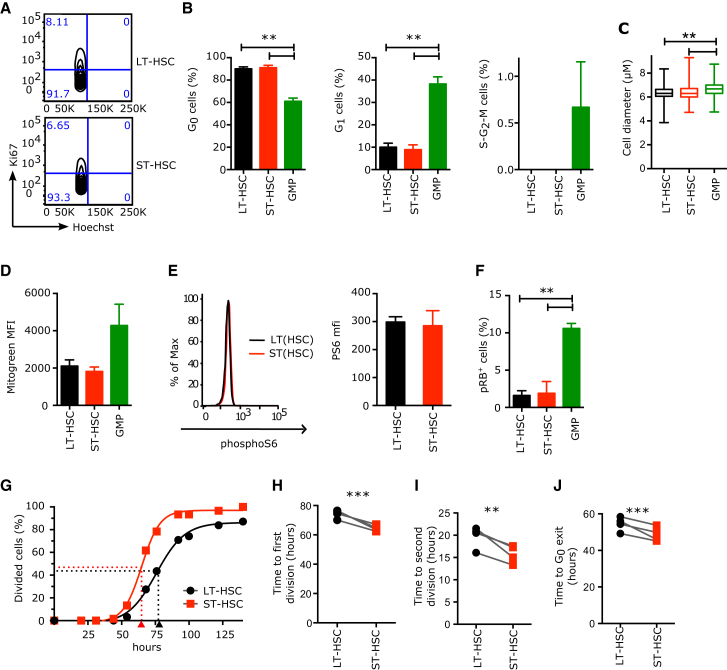
LT- and ST-HSCs Are Equally Quiescent, but upon Mitogenic Stimulation They Differ in the Duration of Divisions Starting from G_0_ or G_1_ (A and B) Proportion of human CB HSC and progenitor cells in each phase of the cell cycle. Parameters were assessed by flow cytometry using Ki67 and Hoechst (Ki67^−^ 2n DNA content, G_0_; Ki67^+^ 2n DNA content, G_1_; Ki67^+^ > 2n DNA content, S-G_2_-M). (A) Representative flow cytometry cell cycle profiles of CB LT- and ST-HSCs and the percentage of cells in each gate. Event count: LT-HSCs (top panel), 1,320 cells; ST-HSCs (bottom panel), 1,143 cells. (B) Mean ± SEM is shown; n = 3 CB samples. (C) Cell diameter of indicated populations measured with ImageJ from microscopy pictures. n > 323 cells from four independent CB samples. (D) Mitochondrial mass as measured by flow cytometry with MitoGreen. MFI, Mean Fluorescence Intensity; mean ± S.E.M shown, n = 2 independent CB samples. (E) PhosphoS6 protein levels as measured by flow cytometry. Left panels: representative flow cytometry plots; black line, LT-HSCs; red line, ST-HSCs. Right panel: median fluorescence intensity of phosphoS6 staining. Mean ± SEM is shown. n = 2 CB samples. (F) Percentage of cells positive for phosphoRB (S807/S811) as measured by flow cytometry; mean ± S.E.M shown, n = 2 independent CB samples. GMP, granulocyte-monocyte progenitors. (G) Cumulative first division kinetics (excluding dead cells) of LT-HSCs (black) and ST-HSCs (red) from a representative CB example. Curve is least-squares sigmoid fit. R^2^ > 0.99. Arrowheads represent time to first division as estimated from sigmoid fit (t_FirstDiv_ = logEC_50_). Time 0 is the time of exposure to mitogenic stimulus. (H) Mean time to first division (in hours). (I) Mean time of cell cycle transit (t_SecondDiv_ = logEC_50_ of sigmoid fit of cumulative second division kinetics; see Figure S2E). (J) Mean time of G_0_ exit (in hours) (t_G0exit_ = t_FirstDiv_ – t_SecondDiv_). In (H)–(J), individual CB samples are shown; gray lines connect LT-HSC and ST-HSC parameters from the same CB. ^∗∗^p < 0.05, ^∗∗∗^p < 0.01 by paired t test. See also Figure S2.

The increased frequency of ST-HSC divisions may be due to (1) more cells actively cycling at any time, (2) increased sensitivity, or (3) faster response to mitogenic stimulation. To resolve the basis for these increased divisions, we investigated the proportions of LT- and ST-HSCs in each cell cycle phase and found them to be identical at all points during the xenotransplantation process (Figure S1B). Freshly isolated from CB, both HSC subsets had more than 90% of cells in G_0_ (Ki67^−^ 2n DNA content, Figures 2A and 2B). Importantly, no cell was found in S-G_2_-M as determined by DNA content (Figures 2A and 2B) and by complete absence of the mitotic marker phosphoH3 (Figure S2A). Cell diameters were equally small in LT- and ST-HSCs (Figure 2C), with both lacking in cytoplasm. Metabolically, both LT- and ST-HSCs showed low mitochondrial activity (Figure 2D) and similar levels of mTOR activation (assessed by phosphoS6 staining; Figure 2E). All these parameters indicate a G_0_ quiescent state. To exclude a possible differential G_1_ arrest state for LT- and ST-HSCs, we analyzed the phosphorylation state of retinoblastoma protein (RB) at S807/S811, a marker upregulated in G_0_ cells before entry into G_1_ (Ren and Rollins, 2004). Both cell types were negative (Figures 2F and S2B). In contrast, granulocyte-monocyte progenitors (GMPs) were largely in G_1_, as most cells were Ki67^+^ with 2n DNA content (Figure 2B) and had a larger diameter (Figure 2C), visible cytoplasm, increased mitochondrial activity (Figure 2D), and RB phosphorylation on S807/S811 (Figure 2F). Collectively, these data establish that both human LT- and ST-HSCs freshly isolated from CB reside in a G_0_ quiescent state lacking all markers of G_1_.

### Distinct Cell Division Durations in HSC Subsets

Since the proportions of LT- and ST-HSCs in G_0_ were identical (Figures 2B and S1B), we hypothesized that the differences in expression of cell cycle genes and frequency of division observed between these two subsets reflected differences in their capacity to exit quiescence upon mitogenic stimulation. Therefore, we measured the duration of single divisions occurring upon activation by a mitogenic signal. Such studies need to be performed with single cells using in vitro assays. We sorted 576 single LT- and ST-HSCs from CB and monitored their divisions over 140 hr in serum-free conditions. As expected, proliferation was higher in ST-HSCs (Figure S2C). The mean time to first division (t_FirstDiv_) varied between CB samples, but on average it was 9 hr shorter in ST-HSCs compared to LT-HSCs (Figures 2G and 2H). The mean time to second division (t_SecondDiv_ = t_G1-S-G2-M_) was also significantly shorter in ST-HSCs than in LT-HSCs (Figures 2I and S2D). Importantly the second division was always shorter than the first, identifying a latency restricted to HSCs that transition out from a non-stimulated quiescent state. In contrast to in vivo repopulation, cells in this assay do not return to G_0_ after division (Figure S2E). Therefore, the latency phenomenon observed in the first division encompasses the events pertaining to the G_0_ to G_1_ transition but may also include portions of early G_1_. For simplicity, it will be hereafter called “G_0_ exit,” and calculated as t_FirstDiv_ – t_SecondDiv_. By this analysis, LT-HSCs egressed from G_0_ less rapidly than ST-HSCs on average by 5.8 hr (Figure 2J). Similar results with slower kinetics were obtained when single LT- and ST-HSCs were cultured in a medium with lower cytokine and nutrient concentrations (Figures S2F and S2G). These parameters are not unique to CB: fully quiescent LT-HSCs isolated from adult bone marrow also displayed a significant delay in G_0_ exit compared to ST-HSCs (Figure S2H). Collectively, upon stimulation by mitogenic signals, the duration of a division is consistently shorter in human ST-HSCs than in LT-HSCs, whether cells need to transition out of quiescence or continuously cycle.

### Distinct Expression of CDK6 Protein in the Quiescent HSC Pool

**Figure 3:**
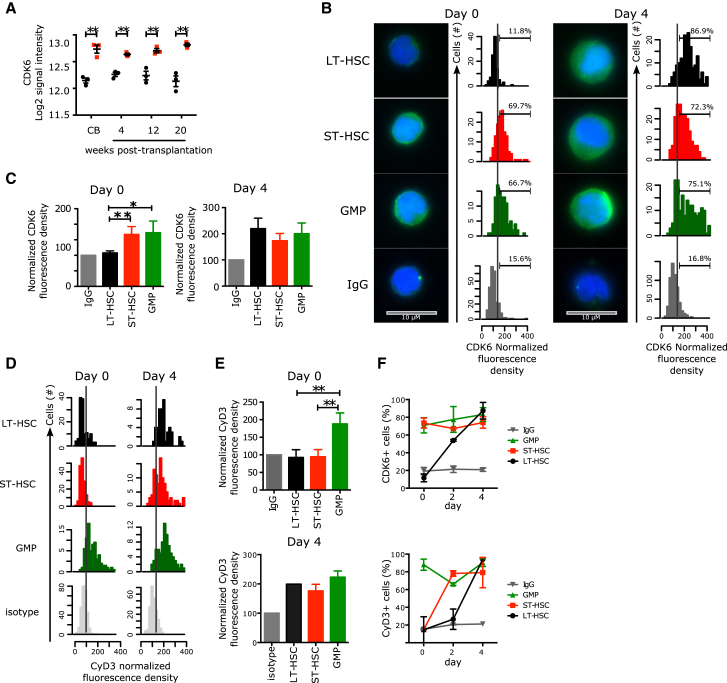
Distinct CDK6 Levels Govern the G_0_ Exit Kinetics of LT- and ST-HSCs (A) Log2 signal intensity for *CDK6* mRNA probe. Shown are individual measures (black circles: LT-HSCs, red squares: ST-HSCs, green triangles: GMPs) and the median and interquantile ranges (horizontal bars); n = 3. All multiple comparisons have been tested. (B) Immunofluorescence for CDK6 protein in LT- and ST-HSCs sorted from CB (left panel) or cultured for 4 days (right panel). Representative pictures and histograms of CDK6 fluorescence density are normalized to the fluorescence density of the IgG control in the same population. Positivity threshold was set over the median + 1 SD of the IgG control distribution and the percetnage of positive cells is indicated. 100–570 cells are analyzed with n = 3 CB samples. Scale bar represents 10 μM. (C) Normalized median CDK6 fluorescence density. Mean ± SEM is shown; n = 3 CB samples. GMPs, granulocyte-monocyte progenitors. (D) Immunofluorescence for CyclinD3 protein in LT-HSCs, ST-HSCs, and GMPs from freshly isolated CB (Day 0, left panel) or after 4 days of culture (Day 4, right panel). Shown are histograms of CyclinD3 fluorescence density normalized to the fluorescence density of the IgG control in the same population. Positivity threshold (dotted line) was set over the median + 1 SD of the IgG control distribution. n = 135–315 cells analyzed for day 0 and n = 49–245 cells for day 4. (E) Normalized median CyclinD3 fluorescence density at the indicated time points. Mean ± SEM is shown; n = 3 CB samples. ^∗∗^p < 0.05 by paired t test. (F) Time course analysis of CDK6 and CyclinD3 upon stimulation by mitogenic signals. Percentages of CDK6^+^ (top panel) or CyclinD3^+^ (bottom panel) cells in each of the indicated populations at the indicated time points after isolation from CB are shown. Mean ± S.E.M shown. n = 3 CB samples, except for day 2, where n = 2 CB samples. ^∗∗^p < 0.05 by paired t test. See also Figure S3.

To identify the molecular determinants underlying differences in the duration of LT- and ST-HSC divisions, we screened the 241-gene core signature for genes known to be involved in either G_0_ exit or G_1_ progression. *CDK6* was selected because its mRNA was consistently upregulated in ST-HSCs both in CB and upon xenotransplantation (Figures 3A, S3A, and S3B), and CDK6/CyclinD complexes regulate G_0_ exit and early G_1_ (Sherr and Roberts, 2004). Importantly, the CDK6 protein was undetectable in most of the quiescent CB LT-HSCs, but it was upregulated after 4 days of culture when all HSC subsets actively cycle (Figures 3B and 3C). In sharp contrast, before culture, ST-HSCs already expressed high levels of CDK6 protein, similar to that found in G_1_ GMPs, despite being quiescent (Figures 3B, 3C, and S3C). Similarly, adult ST-HSCs isolated from either bone marrow or mobilized peripheral blood also expressed high levels of CDK6, though it was undetectable in adult LT-HSCs (Figures S3D–S3F). To gain insight into how ST-HSCs can remain quiescent with high levels of CDK6 protein, and because CDK6 kinase activity depends on its association with CyclinD proteins, we examined the levels of CyclinD1 and CyclinD3 protein in sorted LT- and ST-HSC subsets from purified CB at day 0. We found that neither one expressed CyclinD1 nor CyclinD3; as expected, both were expressed in G_1_ GMPs (Figures S3G, S3H, 3D, and 3E). Therefore the CDK6 in ST-HSCs is not part of an active complex, which explains the absence of RB phosphorylation in these cells (Figure 2F) and their quiescence. To gain insight into how the CyclinD-CDK6 complexes integrate proliferative signals once HSCs are activated, we did a time course analysis of CDK6, CyclinD1, and CyclinD3 protein expression. After 2 days of culture, less than 5% of LT- and ST-HSCs divided (Figure 2G). Interestingly, about 54% of LT-HSCs expressed CDK6 and 44% and 25% express CyclinD1 and CyclinD3, respectively (Figures S3H and 3F). In contrast, almost all ST-HSCs had upregulated CyclinD3 protein by day 2, and 52% express CyclinD1. By day 4, when all LT- and ST-HSCs actively cycle (Figure 2G), each HSC subset expresses both CDK6 and CyclinD3 proteins (Figure 3F). These data indicate that, upon activation by mitogens, the assembly of the CDK/CyclinD complex is more rapid and more robust in ST-HSCs than in LT-HSCs. Overall, these data reveal two unexpected findings. First, ST-HSCs exist in a G_0_ state, yet they express a known driver of G_1_ progression (CDK6) while lacking the cognate partners (CyclinD1 and CyclinD3) of CDK6. Second, the hierarchical organization based on functional repopulation properties also exhibits a hierarchy of CDK6/CyclinD complex components reflecting distinct cycling properties: LT-HSCs are negative for both CDK6 and CyclinD; ST-HSCs express exclusively CDK6; and lineage-restricted progenitors, e.g., GMPs, express both.

### CDK6 Levels Regulate the Duration of G_0_ Exit

**Figure 4:**
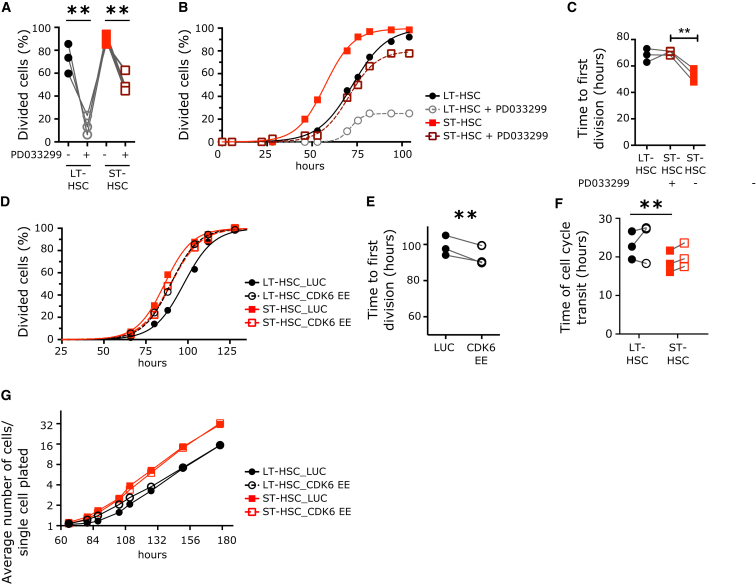
CDK6 Levels Determine the Duration of Quiescence Exit in the HSC Pool (A–C) Cell division duration of single LT- and ST-HSCs after exposure to mitogenic signal in the presence or absence of PD033299 (50 nM). (A) Percentage of cells from the indicated populations that divided after 100 hr in culture. (B) Cumulative first division kinetics (excluding dead cells). Data from a representative CB example are shown. Curve is least-squares sigmoid fit. R^2^ > 0.99. (C) Mean time to first division (hours) (t_firstDiv_ = logEC_50_). (D–G) Cell division duration of single LT- and ST-HSCs after exposure to mitogenic signal with or without CDK6 EE. (D) Cumulative first division kinetics (excluding dead cells) of indicated populations transduced with indicated lentiviral vectors. Data from a representative CB are shown. Curve is least-squares sigmoid fit. R^2^ > 0.99. (E) Mean time to first division (hours) (t_firstDiv_ = logEC_50_). (F) Time of cell cycle transit of indicated populations in hours. (G) Expansion curves of LT- and ST-HSCs in culture. Shown is the average number of cells per single cell plated at the indicated time points after culture initiation. Data are from one representative experiment out of three. Time 0 represents the time of exposure to mitogenic stimulus. In (A), (C), (E), and (F), individual CB samples are shown; gray lines connect parameters from the same condition. ^∗∗^p < 0.05 by paired t test. See also Figure S4.

To gain mechanistic insight into the correlation between CDK6 protein levels and cell division duration within HSC subsets, we altered CDK6 levels and investigated the effect on the kinetics of the first HSC division. To examine loss of function, we measured the duration of cell division when single LT- and ST-HSCs are exposed to a mitogenic stimulus in the presence of the highly specific CDK4-CDK6 inhibitor PD033299. The majority of LT-HSCs never divided in the presence of PD033299 (Figure 4A). Similarly there was a strong reduction in the number of ST-HSCs that could divide (Figure 4A). However, for those ST-HSCs that divided, inhibition of CDK6 brought the length of the first division to that of LT-HSCs (Figures 4B and 4C). Intriguingly, those 10% LT-HSCs dividing in the presence of PD033299 were not further delayed, potentially representing a subset of more “activated” cells within the LT-HSC phenotypic compartment. To examine the consequences of CDK6 gain of function, we enforced expression of CDK6 protein (CDK6 EE) with lentiviral vectors in LT- and ST-HSCs before their first division (Figures S4A and S4B). CDK6 EE did not change any ST-HSC cell cycle parameters (Figures 4D–4F, S4C, and S4E). By contrast, a division starting from G_0_ (first division) of CDK6 EE LT-HSCs was significantly shortened to values similar to those of ST-HSCs (Figures 4D and 4E); control transduced LT-HSCs (LUC) showed no such changes. CDK6 EE did not decrease the duration of a division starting from G_1_ that remained significantly longer than that of ST-HSCs (Figures 4F, S4C, and S4F). Also, CDK6 EE did not affect the long-term proliferative output of LT-HSCs in vitro in conditions where cells do not return to G_0_ (Figure 4G). These approaches show that CDK6 shortens divisions starting from G_0_, but not divisions starting from G_1_. Moreover, variation in CDK6 protein levels between HSC subsets results in active regulation of the duration of the latency that is unique to HSCs transiting out of G_0_, a process we define as G_0_ exit. Importantly, our data also establish that pre-existent CDK6 in ST-HSCs primes them for earlier cell division upon mitogenic stimuli.

### CDK6 EE Confers a Competitive Advantage to LT-HSCs without Exhaustion

**Figure 5:**
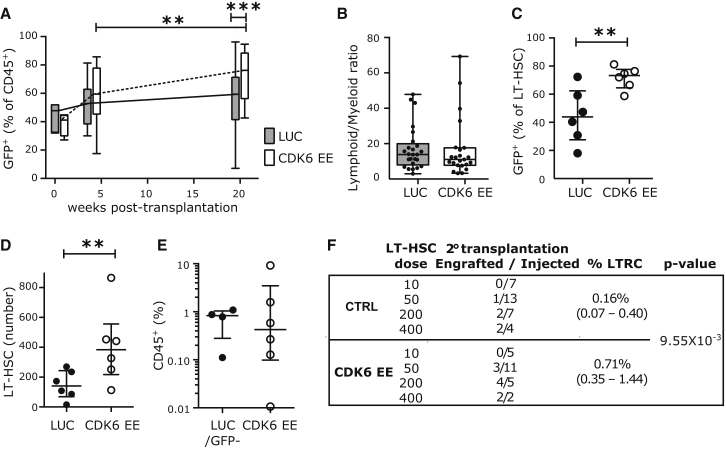
CDK6 EE LT-HSCs Outcompete Wild-Type HSCs without Exhaustion (A–D) NSG mice were injected with sorted Lin^−^ CD34^+^ CD38^−^ cells transduced with CDK6 EE or control (LUC) lentiviral vectors (GFP^+^ cells) and untransduced competitive cells (GFP^−^). Bone marrow was harvested at indicated time points post-transplantation and analyzed by flow cytometry. (A) Percentage of GFP^+^ cells among engrafted human hematopoietic cells (CD45^+^). Time 0 corresponds to percentage of GFP^+^ cells before injection in four independent CB samples. 4 weeks post-transplantation: n = 13 LUC and 14 CDK6 EE mice; 20 weeks post-transplantation: n = 25 LUC and 23 CDK6 EE mice. (B) Lymphoid to myeloid ratio (percentage of CD19^+^/CD33^+^) among GFP^+^ cells at 20 weeks post-transplantation. n = 25 LUC and 23 CDK6 EE mice. In (A) and (B), boxplots represent median, 25^th^, and 75^th^ percentiles and whiskers represent min and max. Gray boxes, LUC; white boxes, CDK6 EE. (C) Percentage of GFP^+^ cells among LT-HSCs at 20 weeks post-transplantation. (D) Absolute number of LT-HSCs at 20 weeks post-transplantation. In (C) and (D), n = 6 mice from two CB samples. Individual mice, median, and interquantile range are shown. In (A)–(D), ^∗^p < 0.1, ^∗∗^p < 0.05, ^∗∗∗^p < 0.01 by Mann-Whitney test. (E and F) CDK6 EE LT-HSCs expand over serial transplantation. LUC, CDK6 EE (GFP^+^), or untransduced (GFP^−^) LT-HSCs were sorted from primary transplanted mice (n = 2 pools of three to five mice) and injected at four different doses into secondary NSG mice. (E) Engraftment levels (percentage of CD45^+^ cells) 12 weeks after secondary transplantation (>0.01% CD45^+^ GFP^+^ or CD45^+^ GFP^−^) at the two highest doses (200 and 400 cells/mouse). Individual mice, median, and interquantile range are shown. (F) Summary table of number of mice engrafted at each dose tested and estimation of LT-HSC frequencies in each group by the ELDA statistical method. See also Figure S5.

In mouse models, failure to maintain quiescence and/or increased cycling are mostly associated with decreased self-renewal and eventual HSC exhaustion (Orford and Scadden, 2008; Pietras et al., 2011; Rossi et al., 2012). To examine the long-term effect of exclusively accelerating the duration of exit from quiescence upon reception of mitogenic stimuli, we enforced CDK6 expression in LT-HSCs in vivo, where HSCs return to G_0_ after most divisions under homeostatic conditions (Wilson et al., 2008 and Figure S1B). Competitive xenotransplantation experiments showed that CDK6 EE does not confer a proliferative advantage within the first 4 weeks post-transplantation, during which HSCs are actively cycling (Figure 5A). However, by 20 weeks post-transplantation, most HSCs have regained quiescence. At this point, CDK6 EE cells, unlike LUC cells, significantly outcompete untransduced cells (median GFP^+^ percentage: LUC, 59.4%; CDK6 EE, 76.2%, p = 0.007, Figure 5A) without displaying any lineage bias (Figures 5B and S5A). This expansion originated from LT-HSCs (Figures 5C and 5D) and extended to all progenitor populations (Figure S5B). To determine whether the CDK6 EE in phenotypic LT-HSCs altered serial repopulating capacity, secondary transplantation was performed. There was no significant difference in the graft size at 12 weeks after secondary transplantation when high numbers of control (LUC/GFP^−^) and CDK6 EE LT-HSCs were transplanted (Figures 5E and S5C). However, limiting dilution analysis revealed a 4-fold increase in the frequency of repopulating LT-HSCs in the CDK6 EE group compared to controls (Figure 5F), confirming LT-HSC expansion over two rounds of transplantation. Importantly, like our in vitro results, CDK6 EE did not change the rate of cell cycle transit of LT- or ST-HSCs (Figure S5D) but accelerated the first division of LT-HSCs (Figure S5E). These data show that the unique shortening in the duration of G_0_ exit conferred by CDK6 EE gives LT-HSCs a competitive advantage without altering self-renewal or differentiation abilities.

### Simulating the Impact of G_0_ Exit Durations on Hematopoietic Homeostasis

**Figure 6:**
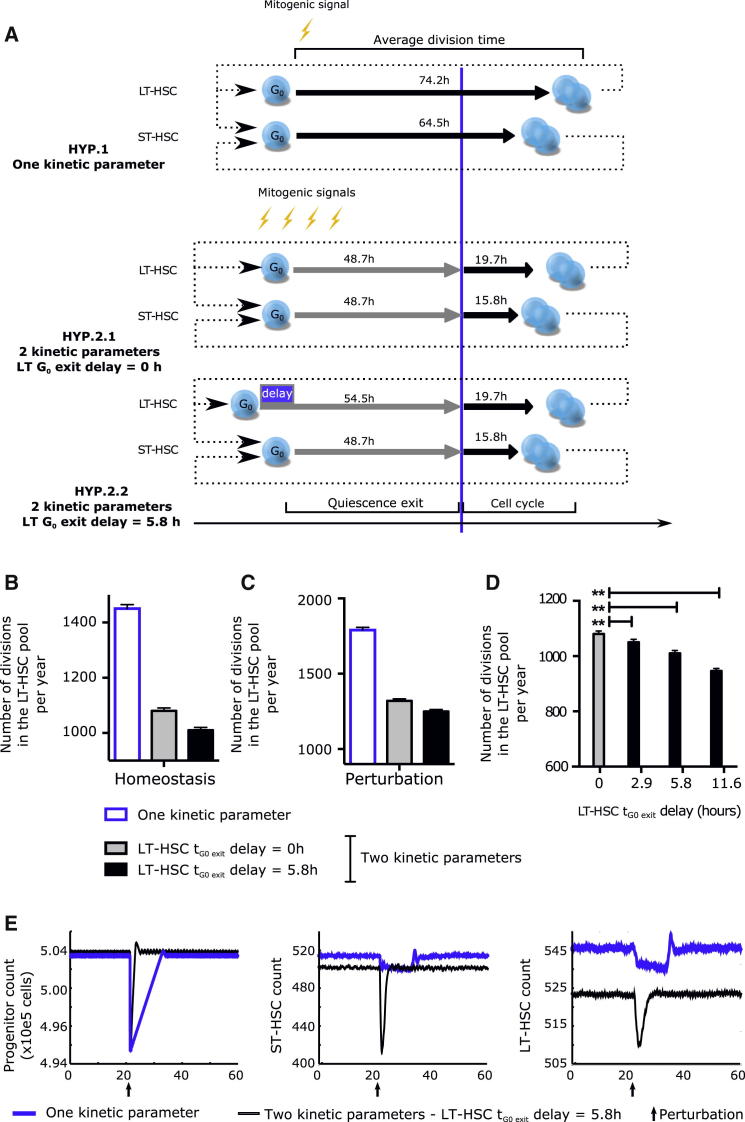
Simulation of the Impact of Delayed G_0_ Exit in LT-HSCs on the HSC Pool with an Agent-Based Model (A) Comparison between the three hypotheses tested by the modeling strategy. Earlier modeling strategies of homeostasis assumed that all HSCs started division upon receiving a mitogenic signal with one characteristic cycling time per HSC subtype (HYP. 1). Rather, we propose that G_0_ exit and cell cycle progression are differentially and independently regulated. This results in two characteristic cycling times per HSC subtype without (HYP. 2.1) or with (HYP. 2.2) a delay in G_0_ exit between LT- and ST-HSCs. Cycling times indicated are as measured in Figures 2G–2J. (B and C) Simulated number of LT-HSC divisions per year in the HSC pool at homeostasis (B) and after perturbation (C). (D) Effect of changing the LT-HSC G_0_ exit delay parameter on the number of LT-HSC divisions. Shown is the number of divisions in the LT-HSC pool per year when the delay of G_0_ exit of LT-HSCs (compared to ST-HSCs) is inputted at 0 (no delay), 2.9, 5.8 (experimental value), or 11.6 hr. (E) Perturbation model: 1% of the progenitor compartment was eliminated at the time indicated by an arrow to simulate injury. Number of progenitor cells (left panel), ST-HSCs (middle panel), and LT-HSCs (right panel) are displayed as a function of time. In (B)–(E), data represent the mean ± SD of 256 runs. The simulations were run with a noise parameter of 5% and an HSC pool exit rate of 24 cells per day. How parameters were chosen and results with different parameters are shown in Figure S6 and discussed in the Supplemental Experimental Procedures.

Our data show that upon activation, LT-HSCs are delayed in their quiescence exit. Because LT-HSCs have been estimated to divide very infrequently, approximately once every 135 and 280 days in mouse and human, respectively (Catlin et al., 2011; Wilson et al., 2008), we sought to quantify consequences of this delay to cell cycle entry in homeostatic conditions. Because it is impossible to experimentally examine homeostatic human HSC pool dynamics over long periods, we turned to computational modeling. Our data strongly suggest that control of cell division is achieved through regulation of quiescence exit and cell cycle transit as two discrete steps. We established an agent-based model to investigate (1) the consequences of independent control of the duration of quiescence exit and (2) the effect of the 5.8 hr delay in LT-HSC quiescence exit. In this model, the maintenance of the number of cells in the system is controlled in a closed loop, and dynamic properties of the model—how often cells divide and how quick the response to injury—arise purely from the different durations of the stages of cell division (Figure S6A). All parameters and assumptions of the model are reported in the Supplemental Experimental Procedures. Most parameters, in particular the division times (mean ± SD), were measured experimentally. When not possible (i.e., HSC pool exit rate and noise), we tested the full range of possible values (discussed in the Supplemental Experimental Procedures) and chose those predicting a number of HSC divisions per year that is in the range reported in the literature for human HSCs (Catlin et al., 2011) (Figures S6B–S6G). With this set of physiologically relevant parameters, we investigated the outcome of (1) a control situation in which division is controlled with one kinetic parameter (from reception of signal to the generation of two daughter cells, with cells committed to divide upon sensing the signal), and (2) a situation in which quiescence exit and cell cycle transit are controlled independently, and where commitment to division happens only once the cell has transitioned out of the quiescence exit phase (Figure 6A). We invariably found that the number of LT-HSC divisions is lower when the duration of a cell division starting from G_0_ is defined by two independent kinetic parameters (quiescence exit and cell cycle transit) rather than a single parameter describing the average division time (Figures 6B and 6C). Furthermore, the overall number of LT-HSC divisions simulated to occur over 1 year was again decreased with a 5.8 hr delay in LT-HSC t_G0 exit_ (Figures 6B and 6C). In fact, a delay as short as 2.6 hr was sufficient to significantly spare the number of LT-HSC divisions (Figure 6D). In addition, in response to perturbation such as might be experienced under hematopoietic stress, the rate of recovery in the HSC and progenitor pools was considerably improved by regulation through two kinetic parameters and even further when the delay in t_G0 exit_ in LT-HSCs was included (Figures 6E, S6H, and S6I). Our model thus demonstrates that the ability to modulate the length of G_0_ exit independently of changes in duration of cell cycle transit provides better robustness to homeostatic and stress response hematopoiesis. Importantly, a delay in the duration of G_0_ exit in LT-HSCs compared to ST-HSCs leads to further optimization, indicating that regulation of the duration of the G_0_ exit phase rather than that of a whole division is key to controlling HSC pool maintenance and hematopoietic system responses.

## Discussion

Our study provides key insights into the regulation of cycling within the HSC pool and furthers our understanding of quiescence. We establish that the level of CDK6 functions as a master regulator of the duration of quiescence exit. CDK6 is differentially regulated at the transcriptional and post-transcriptional level in HSC subsets. In particular, the absence of CDK6 protein in LT-HSCs results in a 5–6 hr delay to G_0_ exit. The cumulative effect of this delay limits LT-HSC divisions and ultimately preserves HSC pool integrity in the long term. Because human HSC possess unique mechanisms to prevent propagation when damaged (van Galen et al., 2014; Milyavsky et al., 2010), we speculate that delayed G_0_ exit may also be crucial to coordinate repair and LT-HSC fate choices upon their exposure to stress.

In line with the importance of the relative levels of the CyclinD-CDK partners in mediating cell cycle entry and progression (Sherr and Roberts, 2004), our data indicate that the presence of CDK6 in ST-HSC is sufficient to place these cells in the “starting blocks” for division upon mitogenic signaling. Production and activation of CyclinD-CDK complexes is gradual and involves many levels of regulation including gene transcription, protein stability, assembly, and nuclear import (Sherr and Roberts, 2004). Consistent with our results a recent study found that a constitutive knockout of CDK6 does not affect HSCs in homeostasis, but their activation in vivo by mitogenic signals such as 5-FU or IFN is prevented (Scheicher et al., 2014). In line with what is seen in HSCs, memory T cells segregate fully formed CyclinD3/CDK6 complexes in their cytoplasm, which, upon antigen stimulation, allow them to enter cell cycle faster than naive T cells wherein both CDK6 and CyclinD3 are expressed at much lower levels (Veiga-Fernandes and Rocha, 2004). Together, these findings support a model in which any molecular configuration that puts cells closer to an active CDK/CyclinD complex is likely to result in faster cell cycle entry/G_0_ exit. Our data further indicate that deeper (LT-HSCs) and shallower (ST-HSCs) states of quiescence are an embedded feature of the hematopoietic hierarchy at homeostasis. Furthermore, the CDK6-primed G_0_ state of ST-HSCs does not overlap with G_alert_, a recently described injury-stimulus-induced adaptive mechanism that positions stem cells to rapidly respond to further stress by activating the mTORC1 pathway (Rodgers et al., 2014). We found that homeostatic ST-HSCs display similar levels of mitochondrial and mTORC activity to that of LT-HSCs, indicating that they are not in G_alert_. Rather, the injury-independent pre-existent diversity in quiescent states that we report coexists with, and is upstream of, G_alert_.

In a high output system like blood, which is sustained by a limited number of active HSCs, a number of theoretical frameworks describe how HSC heterogeneity, notably in division rates, contributes to lifelong maintenance of hematopoiesis. Previous modeling strategies (Glauche et al., 2009; Hoffmann et al., 2008; Roeder and Loeffler, 2002) describe the division properties of HSCs by a single, unique parameter that is usually derived from average division frequencies and thus includes the time spent in G_0_ plus the time from the reception of the signal to the end of division. In contrast to these prior studies, we explicitly model the control of the duration of cell division from the time of the mitogenic signal, using a computational model where the signal is automatically generated depending on needs. This framework allows investigation of which division duration control strategy better preserves HSC pool integrity and maximizes system responsiveness. In this context, “quiescence exit” is a phase during which cells receive and accumulate signals prior to committing to division. Our simulations show that when cell division controlled by two independent characteristic times (one before and one after the commitment point), it is far more efficient than if cells are committed to divide within a fixed period after sensing a signal. Control is further optimized when quiescence exit is differentially regulated between LT- and ST-HSCs. Thus, we propose that, even though its molecular boundaries remain to be defined, the duration of quiescence exit is a biologically relevant time interval and the regulation of this duration inherently determines the dynamics of blood formation. Overall our data point to a model of homeostasis where the deeply quiescent state of LT-HSCs with a long duration of exit from quiescence and the CDK6-primed G_0_ state of ST-HSCs together provide a means to achieve efficient production of cells from ST-HSCs while limiting the number of divisions that LT-HSCs undergo.

Our data show that when the G_0_ exit delay is abolished due to CDK6-enforced expression, the CDK6 EE LT-HSCs divide more in repopulation assays, due to repeated rounds of accelerated G_0_ exits. Accelerating exit from quiescence (at least via CDK6 EE) does not alter the balance between self-renewal and differentiation or impair LT-HSC maintenance; rather, LT-HSCs acquire a competitive advantage. This result is in striking contrast with most situations of increased cycling presented in the literature (reviewed in Pietras et al., 2011) that cause impaired HSC function. Interestingly, two of the most notable examples that increase HSC division without damaging their long-term function are p18 knockout (Yuan et al., 2004) and miR-126 knockdown (Lechman et al., 2012). Because p18 is an Ink4 family member known to repress CDK6, and because our recent data suggest that miR-126 also targets CDK6 (E. Lechman and J.E.D., unpublished data), it will be interesting to verify if the delay in G_0_ exit of LT-HSCs is also suppressed in these models. Similarly, it needs to be addressed how G_0_ exit duration is affected in cases where increased cycling leads to HSC exhaustion. Indeed, why increased cycling is generally associated with impaired LT-HSC maintenance remains hypothetical (Orford and Scadden, 2008; Pietras et al., 2011; Rossi et al., 2012). In view of our own results, we speculate that LT-HSCs may be pushed toward differentiation at the expense of self-renewal if they shorten or bypass phases of the cell cycle other than G_0_ exit, undergo several rounds of division without returning to G_0_, or show imbalances in key differentiation genes. Importantly, our experimental and computational modeling data establish that fully functional LT-HSCs can acquire competitive advantages in a purely kinetic way. Such a phenomenon may be crucial during aging or in the initial steps of leukemia, where clonal dominance may uniquely arise as a consequence of the accelerated duration of G_0_ exit of LT-HSCs. Furthermore, recent work indicates that PD033299, which selectively inhibits CDK6, might be efficacious against multiple myeloma (Huang et al., 2012) and MLL-rearranged AML (Placke et al., 2014), malignancies where the pre-leukemic cell of origin is thought to be an HSC. Overall, the finding that the duration of G_0_ exit is a highly relevant biological parameter that controls stem cell pool dynamics warrants further investigation of whether perturbation of stem cell-specific quiescence exit mechanisms represents an early step of malignancy.

## Experimental Procedures

### CB Lineage Depletion

All CB samples were obtained with informed consent according to procedures approved by the institutional review boards of the University Health Network, Trillium, and Credit Valley Hospital. Mononuclear cells were obtained by centrifugation on Lymphoprep medium (Stem Cell Technologies) and were depleted of Lin^+^ cells (lineage depletion) by negative selection with the StemSep Human Progenitor Cell Enrichment Kit according to the manufacturer’s protocol (Stem Cell Technologies). Lin^−^ CB cells were stored at −150°C.

### Cell Preparation for Cell Sorting

Lin^–^ cells were thawed by drop-wise addition of IMDM/DNase (100 μg/ml, Roche) and were resuspended at 1 × 10^6^ cells /ml. Cells were then stained with the following (with all antibodies from BD, unless stated otherwise): FITC—anti-CD45RA (1:50, 555488), PE—anti-CD90 (1:50, 555596), PECy5—anti-CD49f (1:50, 551129), V450—anti-CD7 (1:33.3, 642916), PECy7—anti-CD38 (1:100, 335790), APC—anti-CD10 (1:50, 340923), and APCCy7—anti-CD34 (1:100, custom made by BD). Cells were sorted on FACS Aria III (Becton Dickinson) or MoFlo (Beckman Coulter) sorters, consistently yielding >95% purity. LT-HSCs were sorted based on the following markers: CD34^+^ CD38^−^ CD45RA^−^ CD90^+^ CD49f^+^; ST-HSCs, based on CD34^+^ CD38^−^ CD45RA^−^ CD90^−^ CD49f^−^; and GMPs, based on CD34^+^ CD38^−^ CD10^−^ CD7^−^ CD45RA^+^.

### Single-Cell Experiments

Single LT-HSCs or ST-HSCs were sorted into 96-well round-bottom Nunc plates in 100 μl of either high or low cytokines media, using FACS Aria III (Becton Dickinson). Cells were centrifuged 5 min at 400 × *g* and incubated at 37°C for 1 week. Cells were visualized and counted in each well twice a day using an inverted microscope. High cytokine condition medium was by StemPro (Stem Cell Technologies) supplemented with StemPro nutrients (Stem Cell Technologies), L-glutamine (GIBCO), Pen/Strep (GIBCO), human LDL (Stem Cell Technologies, 50 ng/ml), and the following cytokines (all from Miltenyi): SCF (100 ng/ml), Flt3L (20 ng/ml), TPO (100 ng/ml), EPO (3 units/ml), IL-6 (50 ng/ml), IL-3 (10 ng/ml), and GM-CSF (20 ng/ml). Low cytokine condition medium was composed of X-VIVO 10 medium (BioWhittaker) supplemented with 1% BSA (Roche), L-glutamine (GIBCO), Pen/Strep (GIBCO), and the following cytokines (all from Miltenyi): SCF (100 ng/ml), Flt3L (100 ng/ml), TPO (50 ng/ml), and IL7 (IL-7; 10 ng/ml).

### Immunofluorescence

5 × 10^3^ sorted LT-HSCs, ST-HSCs, or GMPs sorted by flow cytometry were fixed ovre 10 min at room temperature (RT) in PBS and 2% paraformaldehyde, washed in PBS, distributed in 150 μl of PBS on polylysine-coated slides, and incubated overnight in a humidified chamber at RT. Cells were then permeabilized over 10 min in 0.2% Triton (SIGMA), washed twice in PBS, and blocked over 20 min using 150 μl of PBS and 10% Goat Serum (Life Technologies). Cells were stained over 1 hr at RT in 150 μl of primary antibody solution in PBS and 10% Goat Serum with appropriate concentrations (CDK6, mouse monoclonal ab54576, Abcam, or CDK6 B-10, Santa Cruz sc7961; mouse IgG, Santa Cruz sc-2025; Cyclin D3 (C-16), Santa Cruz: sc-182; rabbit IgG, Santa Cruz sc-2027). After cells were washed twice in PBS, secondary antibody solution (goat anti-mouse Alexa 488, Life Technologies, A11001) was added over 45 min at RT in the dark in 150 μl PBS (10% Goat Serum) with the appropriate concentration (usually 1:500). Slides were visualized on an Axioimager microscope and fluorescence quantification and cell diameter measurements were performed with ImageJ software.

Cell cycle analysis assay, xenotransplantation, the derivation of cell cycle parameters, modeling, transcriptome studies, bioinformatics, qPCR, lentiviral transduction, and mitochondrial mass measurements are reported in the Supplemental Experimental Procedures.

## Author Contributions

E.L. and J.E.D designed the study; E.L., S.X., C.F. and C.F. analyzed and interpreted the data; E.L., S.X., C.F., R.F, S.Z., and SD performed experiments; C.F.D. performed computational modeling; A.N. and I.P. cloned lentiviral vector plasmids and did RT-PCR; J.C. and C.A. did gene-expression profiling experiments;, J.B.F. and N.I. supervised specific experiments; E.L. wrote the manuscript; E.L., S.X., C.F., R.F, C.F.D, N.I., and J.E.D edited the manuscript; and J.E.D. supervised the study. E.L., C.F., and S.X. contributed equally to this study.

## Accession Numbers

The GEO database accession number for the gene expression data reported in this paper is GSE58299.

## Supplemental Information

Document S1. Figures S1–S6, Table S1, and Supplemental Experimental Procedures

Table S2. List of 241 Genes Differentially Expressed between LT- and ST-HSCs as Determined by BETR Algorithm, Related to Figure 1

Document S2. Article plus Supplemental Information
