# Supplementary material for: CDK6 Levels Regulate Quiescence Exit in Human Hematopoietic Stem Cells
Source: Cell Stem Cell. 2015 Mar 5;16(3):302–13. doi: 10.1016/j.stem.2015.01.017 (PMC4359055; doi:10.1016/j.stem.2015.01.017)
Supplement: Document S1. Figures S1–S6, Table S1, and Supplemental Experimental Procedures [file mmc1.pdf]

Cell Stem Cell
Supplemental Information

CDK6 Levels Regulate Quiescence Exit in Human Hematopoietic Stem Cells

Elisa Laurenti, Catherine Frelin, Stephanie Xie, Robin Ferrari, Cyrille F. Dunant, Sasan Zandi, Andrea Neumann, Ian Plumb, Sergei Doulatov Jin Chen, Craig April, Jian-Bing Fan, Norman Iscove, and John E. Dick

Supplemental Information

Figure S1

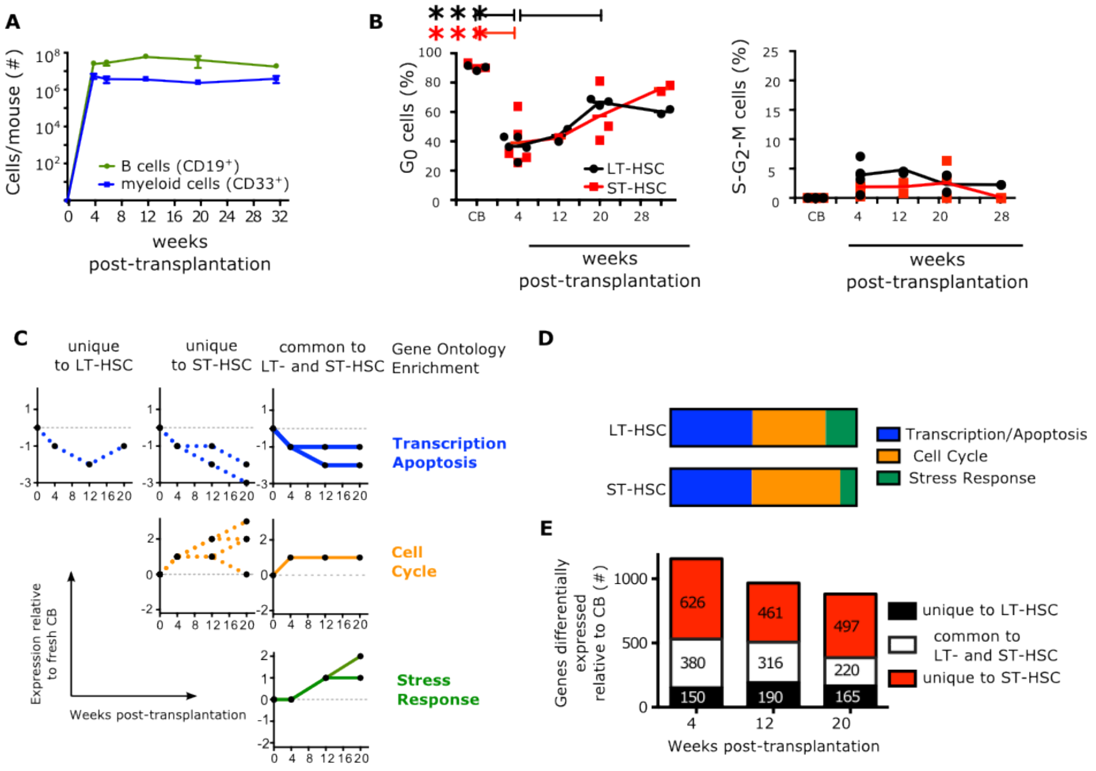

Figure S1, related to Fig.1: Human LT- and ST-HSC functional and transcriptome kinetics upon xenotransplantation.

A, Kinetics of reconstitution of the lymphoid (CD19$^{+)}$ and myeloid lineage (CD33^{+}) in the bone marrow of xenotransplanted mice. Number of cells per mouse of the indicated populations at the indicated time points post transplantation of 70000 Lin^{-} CB (saturating number of LT-HSCs). Median and interquantile range shown. **B,** Quantification of LT-, ST-HSC and progenitor cells in G_0 (Ki67^{-} Hoechst^{-} assayed by flow cytometry as in CB or at the indicated time points post-transplantation. Each point represents individual pools of CB or individual pools of 3-5 mice, and the horizontal bars represent the mean in 2 to 4 independent experiments; black: LT-HSCs, red: ST-HSCs. ***: $p<0.01$ by ANOVA and Tukey test. **C,** Significant gene

expression profiles observed upon transplantation into NSG mice identified by the STEM algorithm. Lines connecting points represent changes in gene expression relative to CB for each profile. Branching indicates multiple possible behaviors for that group of genes. Profiles with significant correlation were grouped in transcriptional programs (rows), and selected Gene Ontology terms associated with each program are shown in the right panel. **D,** Proportion of genes (%) changed across the time course belonging to the transcriptional programs indicated. **E,** Number of differentially expressed genes in LT- or ST-HSC at the indicated time points after transplantation relative to the same population in CB as determined by analysis with the limma algorithm (FDR<0.05).

Figure S2

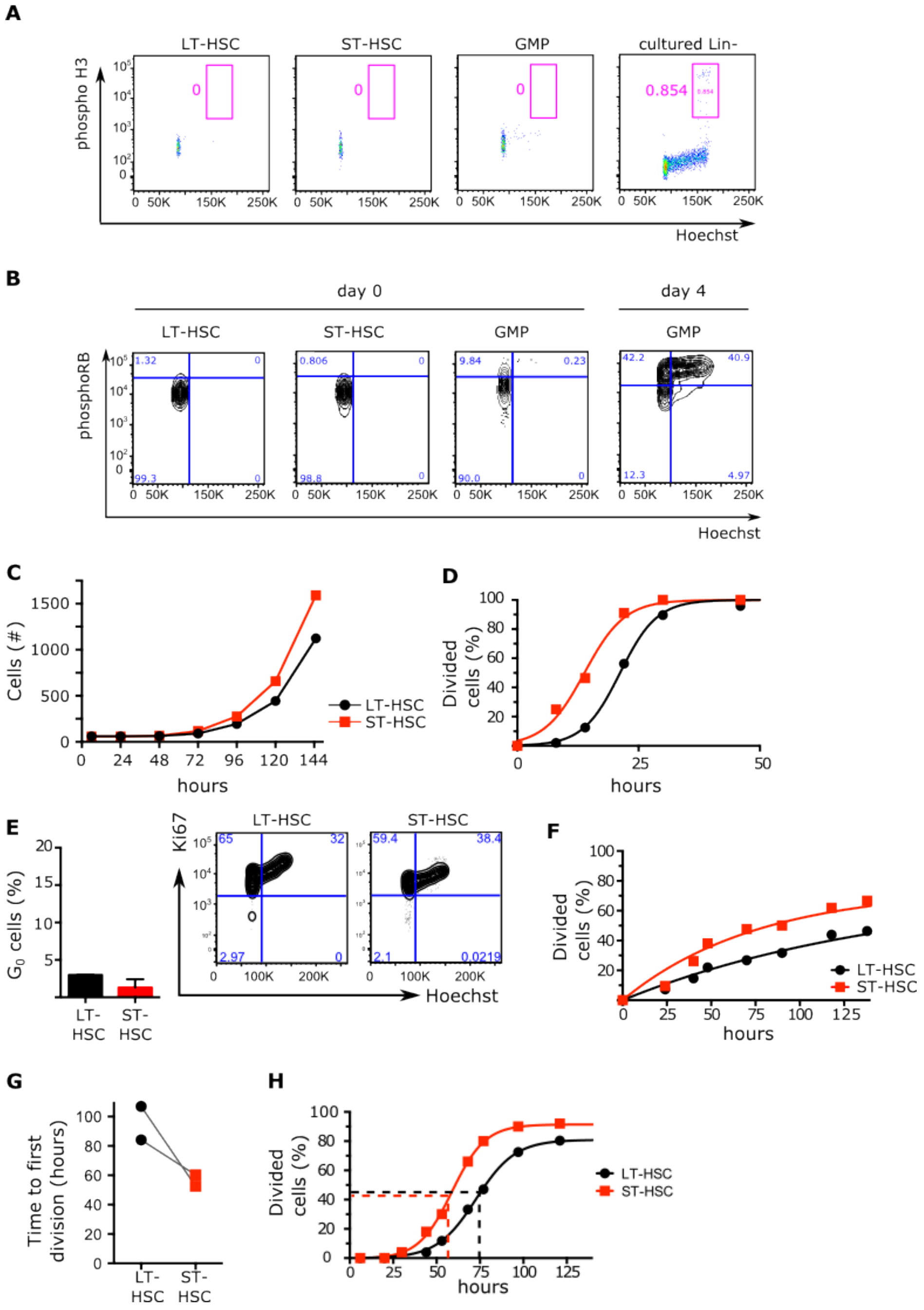

Figure S2, related to Fig.2: characterization of LT- and ST-HSC quiescent states and duration of division upon mitogenic stimulation.

A, absence of phosphoH3 in LT- and ST-HSCs isolated from CB. Examples of representative flow cytometry plots. Pink box represents the gate for phosphoH3 positive cells. n=2 CB. **B,** phosphoRB is absent in LT- and ST-HSCs isolated from

CB. Representative flow cytometry plots of phosphoRB staining (S807/S811 phosphorylation). Cells in the indicated populations either freshly isolated from CB (top panel: day 0) or cultured for 4 days (positive control for the staining, right panel). **C,** ST-HSCs proliferate faster than LT-HSCs in vitro. Growth curve of LT- (black) and ST-HSCs (red), one representative CB is shown, number of initial cells: LT-HSCs: 61, ST-HSC:58. **D,** Cumulative second division kinetics of LT-HSCs (black) and ST-HSCs (red) from a representative CB example. Curve is best sigmoid fit (least squares). R^2 >0.99. **E,** Percentage of cells in the G_0 phase of the cell cycle after 3 days of culture, as measured by flow cytometry ($Ki67^-$ 2n DNA content), representative plot in right panel, quantification from n=2 independent CBs in left panel. **F,G:** In vitro proliferation assay of single LT-HSCs and ST-HSCs in low cytokine and nutrient conditions. n>59 cells from 2 independent CBs. **F,** Cumulative first division kinetics of LT-HSCs (black) and ST-HSCs (red) from a representative CB example. Curve is fit from one-phase association. R^2 >0.96. **G,** Time to first division in hours (half-time). **H,** Cumulative first division kinetics (excluding dead cells) of LT-HSCs (black) and ST-HSCs (red) from a representative adult bone marrow example. Curve is best sigmoid fit (least squares). R^2 >0.99. Arrowheads represent time to first division as estimated from sigmoid fit ($t_{FirstDiv}$ = logEC50).

Figure S3

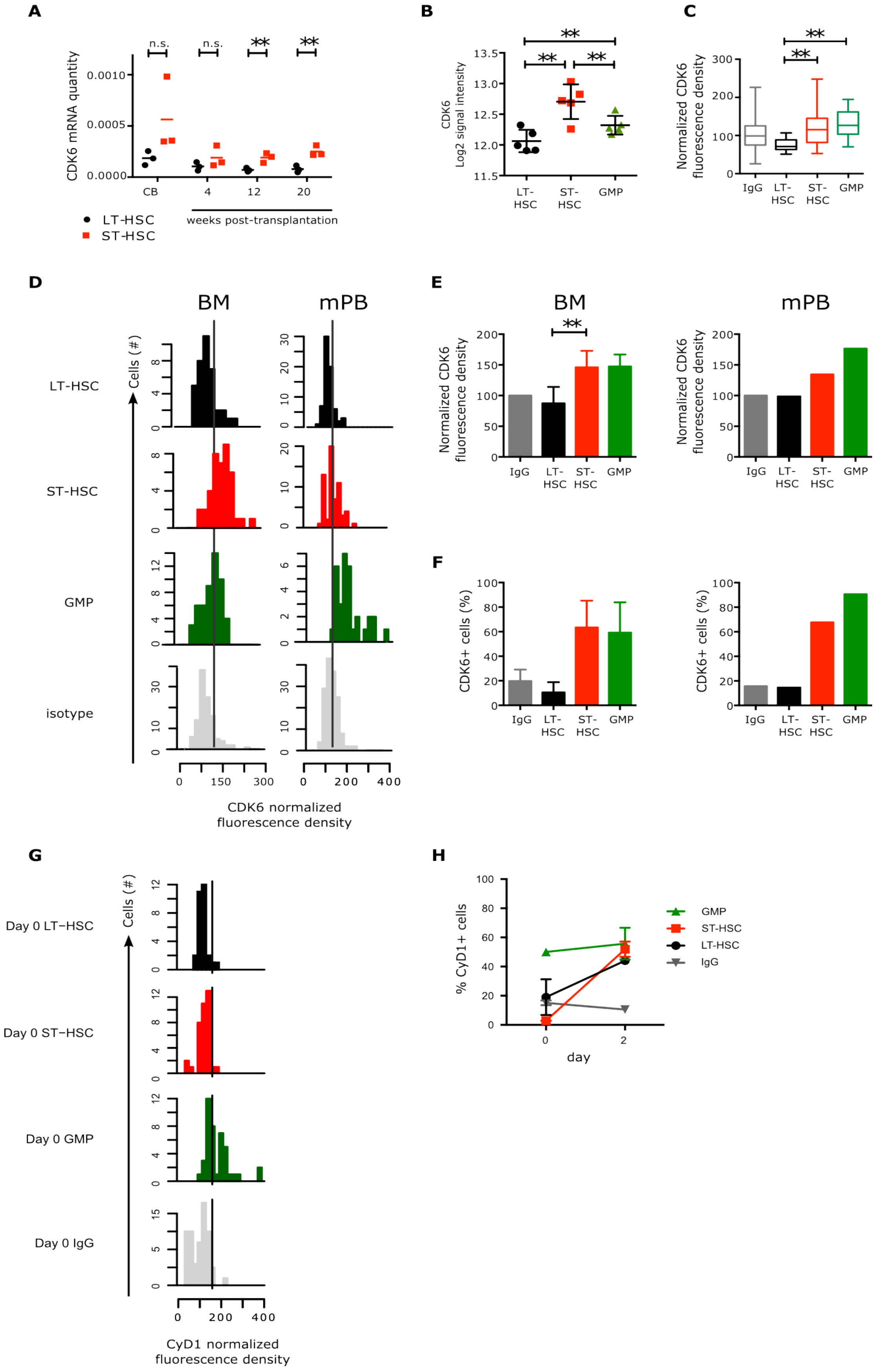
A
CDK6 mRNA quantity
0.0010
0.0005
0.0000
n.s.
n.s.
**
**
CB
4
12
20
weeks post-transplantation
LT-HSC
ST-HSC
B
CDK6
Log2 signal intensity
13.5
13.0
12.5
12.0
11.5
**
**
**
LT-HSC
ST-HSC
GMP
C
Normalized CDK6 fluorescence density
300
200
100
0
**
**
IgG
LT-HSC
ST-HSC
GMP
D
BM
mPB
LT-HSC
ST-HSC
GMP
isotype
Cells (#)
0
150
300
0
200
400
CDK6 normalized fluorescence density
E
BM
mPB
Normalized CDK6 fluorescence density
200
150
100
50
0
**
IgG
LT-HSC
ST-HSC
GMP
F
CDK6+ cells (%)
100
80
60
40
20
0
IgG
LT-HSC
ST-HSC
GMP
G
Day 0 LT-HSC
Day 0 ST-HSC
Day 0 GMP
Day 0 IgG
Cells (#)
0
200
400
CyD1 normalized fluorescence density
H
% CyD1+ cells
100
80
60
40
20
0
0
2
day
GMP
ST-HSC
LT-HSC
IgG

Figure S3, related to Fig.3: CDK6 is transcriptionally and post-transcriptionally upregulated in ST-HSCs across ontogeny.

A, *CDK6* mRNA is expressed at significantly higher levels in ST-HSCs than in LT-HSCs purified from CB. *CDK6* mRNA quantity as measured by qRT-PCR at the indicated time points. mRNA quantity was normalized to the geometric mean of 2 housekeeping genes (*GAPDH* and *ACTB*). All multiple comparisons have been tested. **B,** Log2 signal intensity for the *CDK6* microarray probe in the dataset attached to this manuscript. Mean ± S.E.M. shown. **A,** n=3; **B,** n=5. **C,** CDK6 protein levels in CB assessed with a different antibody from that used in Fig. 3. Boxplots represent median, 25th and 75th percentiles, whiskers represent min and max. n=33-129 cells from 2 independent CB samples. **D,** CDK6 protein expression in LT- and ST-HSCs isolated from adult hematopoietic tissues. Immunofluorescence for CDK6 protein in LT- and ST-HSCs sorted from adult bone marrow (BM, left panel) or mobilized peripheral blood (mPB, right panel). Shown are histograms of CDK6 fluorescence density normalized to the fluorescence density of the IgG control in the same population. Positivity threshold (dotted line) was set over the median ± 1s.d. of the IgG control distribution. n= 75-194 cells analyzed for BM and n=32-152 cells for mPB. **E,** normalized median CDK6 fluorescence density. Mean ± SEM shown. Left panel: BM; Right panel: mPB. **F,** Percentage of $CDK6^{+}$ cells. Mean ± SEM shown; n=2 BMs and n=1 mPB. **: p<0.05 by paired t-test. **G-H**: Time course analysis of CyclinD1 upon stimulation with mitogenic signals Immunofluorescence for CyclinD1 protein in LT-, ST-HSCs and GMPs from freshly isolated CB (**G**) and 2 days after mitogenic stimulation (**H**). **G:** shown are histograms of CyclinD1 fluorescence density normalized to the fluorescence density of the IgG control in the same population. Positivity threshold (black line) was set over the median + 1s.d. of the

IgG control distribution. **H:** Percentage of CDK6 (top panel) or CyclinD3 (bottom panel) positive cells in each of the indicated populations at the indicated time points after isolation from CB. n=61-191 cells analyzed for day 0 and n=31-156 cells for day 2. Mean ± S.E.M. shown.

Figure S4

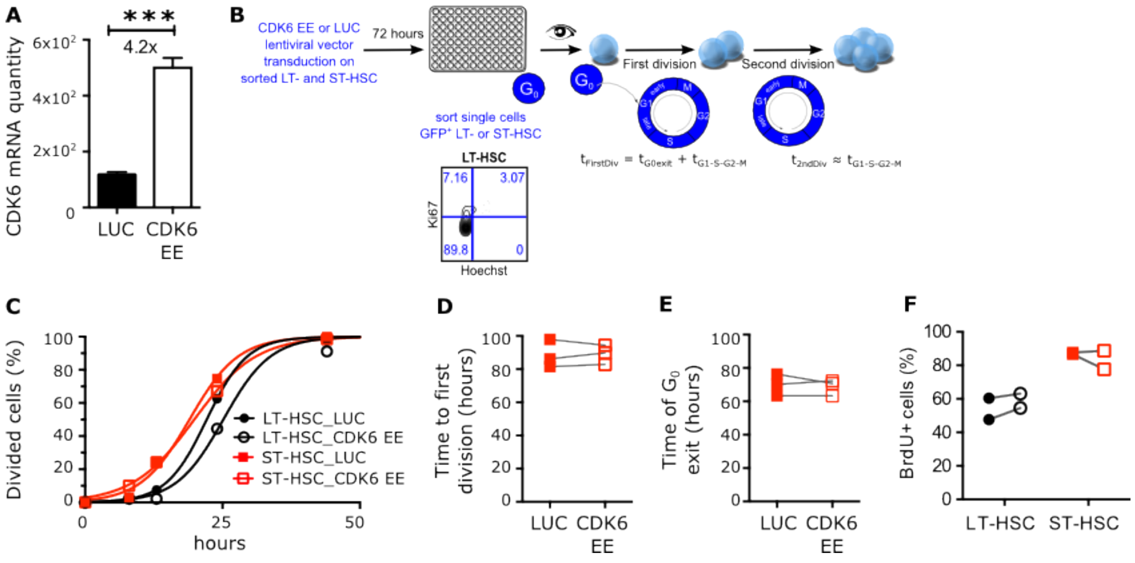

Figure S4, related to Fig.4: CDK6 EE does not affect ST-HSCs.

A, mRNA levels of *CDK6* in Lin⁻ CB cells 3 days after transduction with LUC or CDK6 EE virus. Values are normalized to *GAPDH* and *ACTB*. Mean ± S.E.M. shown. n=3 CB. **B-E:** single cell tracking of LUC or CDK6 EE LT- and ST-HSCs. **B,** Schematic representation of the experiment. Importantly after transduction, cells are cultured in low cytokine and nutrient conditions and do not exit G_0 by the time of the second sort. One representative flow cytometry plot shown (lower panel). Sorted single GFP^+ cells are cultured in high cytokine and nutrient conditions and divisions are monitored. Time 0 represent the time of exposure to mitogenic stimulus. **C,** Cumulative second division kinetics of LT-HSCs (black) and ST-HSCs (red) transduced with LUC (solid symbols) or CDK6EE (empty symbols) from a representative CB example. Curve is best sigmoid fit (least squares). R^2 >0.99. **D,** Time to first division of LUC or CDK6 EE ST-HSC. **E,** Time of G_0 exit LUC or CDK6 EE ST-HSC. ***: p<0.01 and **: p<0.05 and n.s.: non significant by paired t-test. **C-E:** LT-HSCs: black; ST-HSCs: red; filled symbols: LUC; empty symbols:

CDK6 EE. **F.** Relative measure of cell cycle progression through a short pulse of BrdU incorporation**.** LT- and ST-HSCs were transduced with either control (LUC, full symbols) or CDK6 EE (empty symbols), cultured for 4 days then exposed to 100uM BrdU for 8 hours. % BrdU+ cells was then assessed by flow cytometry. n=2 CB

Figure S5

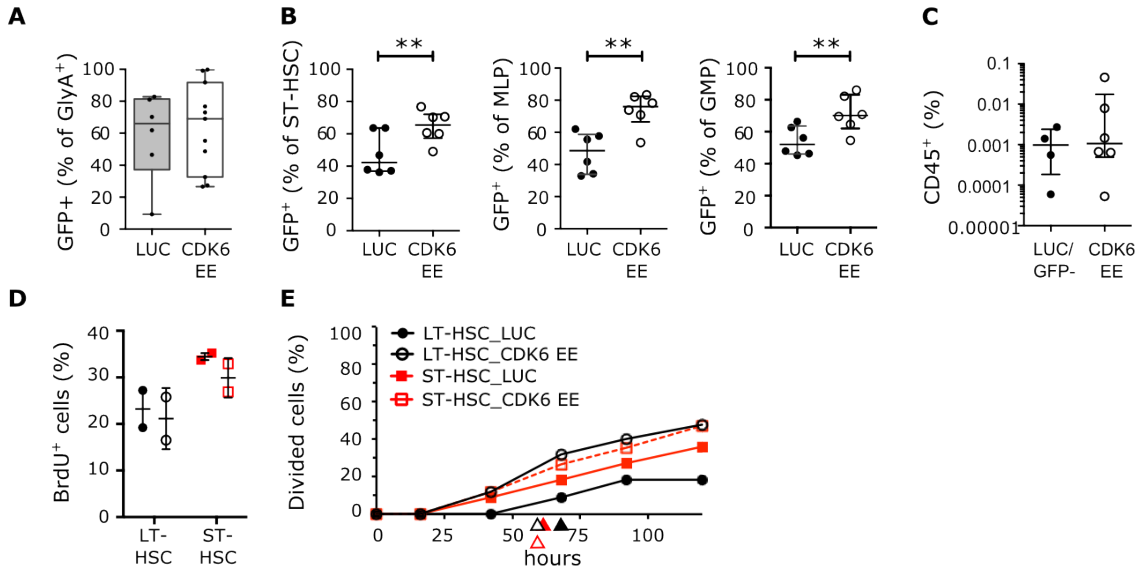

Figure S5, related to Fig.5: CDK6 EE in vivo expands all progenitor populations but does not decrease HSC self-renewal

Competitive xenotransplant experiments were carried out as detailed in Fig. 5. **A**, % GFP$^+$ among engrafted human erythroid cells (GlyA$^+$). Mice that had less than 0.3% GlyA+ cells were excluded. Boxplots represent median, 25th and 75th percentiles, whiskers represent min and max. n=6 mice for LUC, n=11 mice for CDK6 EE. **B,** Competitive advantage of CDK6 EE LT-HSC persists in downstream progenitors. % GFP$^+$ among ST-HSC, MLP and GMP at 20 weeks post-transplantation. n=6 mice from 2 CB. Individual mice, median and interquantile range shown. **: $p<0.05$ by Mann-Whitney test. MLP: Multilymphoid Progenitors, GMP: Granulocyte-Monocyte progenitors. **C,** The repopulation potential of CDK6 EE LT-HSCs does not decrease upon secondary transplant. Shown is the % human CD45$^+$ cells relative to the number of CDK6EE or control (LUC) LT-HSCs injected into secondary xenografts. The analysis was performed 12 weeks after secondary transplantation. Shown are

individual mice. n=4 for LUC and n=6 for CDK6 EE. **D,E**: CDK6 EE division kinetics in vivo. **D,** CDK6 EE does not increase BrdU incorporation in vivo. Mice transplanted with CDK6 EE or control (LUC) transduced cells were administered BrdU for 2 days at 8 weeks post-transplantation. The percentage of BrdU positive cells was assayed by flow cytometry in CDK6 EE or LUC LT- and ST-HSCs. Shown are individual measures (circles, LT-HSCs; squares, ST-HSCs) and median (horizontal bar). n=2 pools of 3-5 mice. **E,** CDK6 EE LT-HSCs harvested from xenografts perform their first division as fast as ST-HSCs. Single LT- and ST-HSCs with LUC or CDK6 EE were isolated from xenografted mice at 20 weeks post-transplantation and cultured in cytokine conditions promoting HSC self-renewal (SCF, TPO, FLT3). Divisions were monitored over time. Arrowheads indicate time to first division. n=25-66 cells from pools of 2-3 mice.

Figure S6

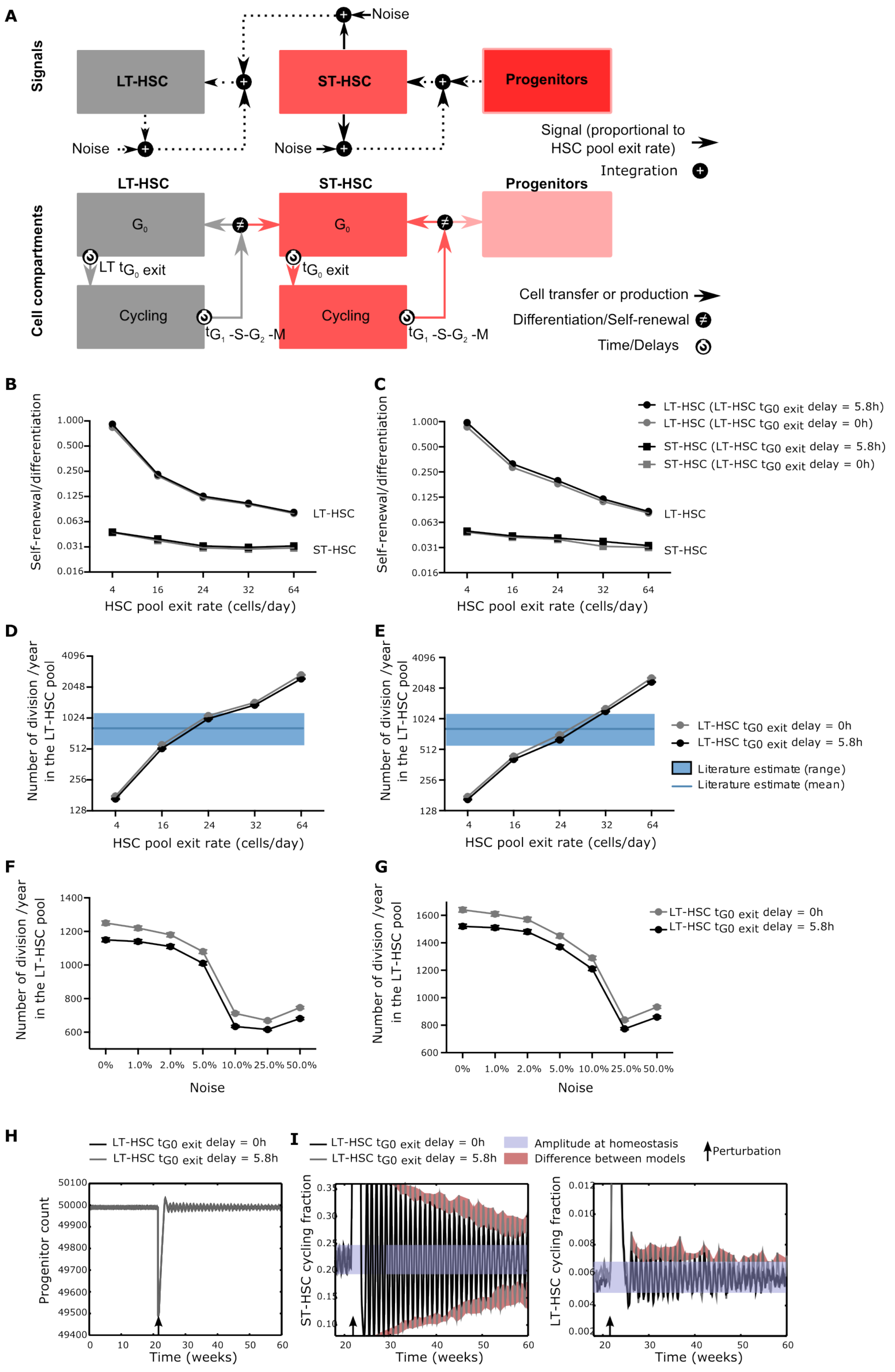
A
Signals
LT-HSC
ST-HSC
Progenitors
Noise
Signal (proportional to HSC pool exit rate)
Integration
Cell compartments
G0
Cycling
LT tG0 exit
tG0 exit
tG1 -S-G2 -M
Cell transfer or production
Differentiation/Self-renewal
Time/Delays
B
C
Self-renewal/differentiation
HSC pool exit rate (cells/day)
LT-HSC (LT-HSC tG0 exit delay = 5.8h)
LT-HSC (LT-HSC tG0 exit delay = 0h)
ST-HSC (LT-HSC tG0 exit delay = 5.8h)
ST-HSC (LT-HSC tG0 exit delay = 0h)
D
E
Number of division /year in the LT-HSC pool
LT-HSC tG0 exit delay = 0h
LT-HSC tG0 exit delay = 5.8h
Literature estimate (range)
Literature estimate (mean)
F
G
Noise
H
I
Progenitor count
Time (weeks)
ST-HSC cycling fraction
LT-HSC cycling fraction
Amplitude at homeostasis
Difference between models
Perturbation

Figure S6, related to Fig.6 and Supplemental Experimental procedures: Agent-based model of the impact of LT-HSC G_0 exit delay on hematopoiesis.

A, Schematic representation of the agent-based model. Top panel: the signals that push cells into division move upstream as indicated by the dashed arrow. Bottom panel: hierarchical organisation of populations and how cells move from one compartment to the other (solid arrows) upon division. Moving from any state (G_0 to Cycling, Cycling to G_0) is characterised by a time unique to the state and cell type as measured experimentally and indicated in Fig.6A. **B-E**: Effect of modifying the HSC pool exit rate. **B-C,** Self-renewal/differentiation ratio of LT- and ST-HSCs as a function of the HSC exit rate (cells/day) when the simulations were run either with 5% (**B**) or 10% (**C**) noise. **D-E:** number of divisions in the LT-HSC pool (500 cells) per year as a function of the HSC exit rate (cells/day) when the simulations were run either with 5% (**D**) or 10% (**E**) noise. The blue band represents the range of LT-HSC division per year based on experimental estimations (Catlin et al., 2011). **F-G,** Effect of modifying noise levels. Number of divisions in the LT-HSC pool (500 cells) per year as a function of the noise (%) when the simulations were run either with an exit rate of 24 (**F**) or 32 (**G**) cells/day. **H-I** Perturbation model: 1% of the progenitors compartment was eliminated at the time indicated by an arrow to simulate injury. Shown are the simulations run with the noise parameter at 5%. **H,** Number of progenitor cells as a function of time. **I,** Fraction of HSC cycling as a function of time. Left panels: ST-HSC; Right panels: LT-HSCs. Homeostatic amplitude is highlighted by a light purple box, while the difference between the model with LT-HSC t_{Goexit} delay = 0 hours and that with LT-HSC t_{Goexit} delay = 5.8 hours is shown in the red shaded area. The data relative to this figure panels are also discussed in Supplemental Experimental Procedures.

Table S1, related to Fig.1: Limiting dilution analysis of cell populations isolated from xenografts at the time indicated.

Time of isolation after primary transplantation	Cell surface phenotype	Dose (cells)	Secondary transplantation Engrafted /Total mice	% LTRC	p-value
4 weeks	$CD49f^+$ $CD90^+$	50	2/11	0.48%	$1.91 x 10^{-3}$
		300	4/5	(0.20 - 1.10)	
	$CD49f^-$ $CD90^-$	50	0/8	0 - 0.19	
		300	0/4		
12 weeks	$CD49f^+$ $CD90^+$	150	2/6	0.40%	$2.64 x 10^{-3}$
		750	6/6	(0.17 - 0.92)	
	$CD49f^-$ $CD90^-$	150	0/6	0.04%	
		750	2/6	(0.01 - 0.17)	
20 weeks	$CD49f^+$ $CD90^+$	10	0/2	1.28%	$1.34 x 10^{-6}$
		50	4/6	(0.61 - 2.70)	
		200	6/6		
		400	4/4		
	$CD49f^-$ $CD90^-$	50	0/3	0 - 0.15	
		200	0/5		
		400	0/2		

Primary xenograft mice were transplanted with 70000 Lin^- cells to establish robust human engraftment. At the indicated time points after primary transplantation (4, 12 or 20 weeks, first column), Lin^- $CD34^+$ $CD38^-$ $CD45RA^-$ $CD49f^+$ $CD90^+$ and Lin^- $CD34^+$ $CD38^-$ $CD45RA^-$ $CD49f^-$ $CD90^-$ were purified from pools of 5-9 primary mice, and were transplanted at the indicated doses in secondary mice. All secondary xenografts were sacrificed 12 weeks after secondary transplantation to verify human engraftment. Engrafted mice: >0.05% human $CD45^+$ in injected femur and non-injected bones. LTRC: Long term repopulating cells. These data collectively show that the same cell surface marker phenotypes used to purify LT- and ST-HSCs out of cord blood can be used to purify LT- and ST-HSCs enriched populations after transplantation into NSG mice.

Table S2, related to Fig.1: list of 241 genes differentially expressed between LT- and ST-HSCs as determined by BETR algorithm.

Laurenti_TableS2.xlsx

Supplemental Experimental Procedures

Cell cycle analysis assays

For Ki67-Hoechst and pRB assays cells were fixed after thawing or flow-sorting with Cytofix/Cytoperm buffer (BD) for 15 min on ice. Cells were stained with FITC-anti-Ki67 (1:50, BD, 556027) or anti-pRB (S807/811, 1:30, Cell Signalling, #8516) overnight in Permwash buffer (BD). For pRB staining, cells were then washed and stained with anti-rabbit IgG Alexa488 (1:500, Life Technologies, A11008) for 30 min, then with Hoechst 33342 (1:10,000; Life Technologies) for 5 min at RT. Samples were analyzed on a LSRII cytometer (BD). For assessment of HSC proliferation in vivo using BrdU incorporation assays, mice were administered drinking water complemented with 4% sucrose and 1 mg/mL BrdU for a 2 to 10 days. Mice were then sacrificed at the indicated timepoints; bone marrow and human CD34+ cells were enriched as described above. Cells were stained with FITC–anti-CD45RA (1:50), PE–anti-CD90 (1:50), PECy5–anti-CD49f (1:50), PECy7–anti-CD38 (1:100), APCCy7–anti-CD34 (1:100), then the BrdU staining was performed with the APC BrdU Flow Kit (BD Pharmingen) with APC-anti-BrdU according to the manufacturer's protocol.

Xenotransplantation

Animal experiments were done in accordance to institutional guidelines approved by University Health Network Animal care committee. Treatments were randomized. NSG mice (NOD.Cg-*Prkdc*scid*Il2rg*tm1Wjl/SzJ; Jackson Laboratory) were sublethally irradiated (250 rads) 24 hours before intrafemoral injection. For xenografts with saturating number of HSC, 6-7x10^4 Lin- CB cells were transplanted per mouse (males or females, 12-16 weeks old), directly after thawing. Competitive

transplantation was performed by injecting each mouse with the equivalent of $1x10^4$ $CD34^+$ $CD38^-$ cells after 4 days of culture in low cytokine conditions medium. In limiting dilution secondary transplantation assays, the indicated sorted populations were injected at defined doses in female mice (12-16 weeks old). LT-HSC frequency was estimated using the ELDA software (http://bioinf.wehi.edu.au/software/elda/; (Hu and Smyth, 2009). For analysis, mice were euthanized at the indicated time points after transplantation. The injected femur and other bones were flushed separately in Iscove's modified Dulbecco's medium (IMDM) and cells were stained with: (dilution of 1:100 and all from BD and unless specified otherwise): phycoerythrin–anti-CD19 (349209), PE–anti-GlyA (Beckman Coulter, A07792), PECy5–anti-CD45 (Beckman Coulter, A07785), PECy7–anti-CD14 (1:200; Beckman Coulter, A22331), APC–anti-CD33 (551378) and V450–anti-CD15 (642917). For LDA experiments 2 CD45 antibodies were used: PECy5–anti-CD45 (Beckman Coulter, A07785) and APC-Cy7-anti-CD45 (557833). For purification of human HSC populations from xenotransplanted mice, bone marrow from individual mice or from pools of 2-10 mice was first enriched for CD34+ cells either by negative selection with the StemSep Human Progenitor Cell Enrichment Kit (Stem Cell Technologies) or by positive selection with the CD34 Microbead kit (Miltenyi) and double column purification by autoMACS technology (Miltenyi). CD34+ enriched cells were then either directly analyzed by flow cytometry or flow-sorted.

Derivation of cell cycle parameters

Single cell experiments data were analyzed with a custom-made R script (version 2.15.2). Briefly the script first excludes all wells with either no cell or more than one cell reported at the beginning of the experiment. The number of cells that have

divided at least once at each time point is then counted. Cumulative first division counts are then plotted over time. Cells that died without dividing are excluded. Similar curves were derived for the second division. The curves obtained by this method were fitted with GraphPad Prism Software (version 6.0b). For high cytokine medium experiments, curves were fitted with a sigmoid model with variable slope. For low cytokine medium conditions curves were fitted with a one-phase association model. Best fits were evaluated with R^2 corrected value. EC50 was calculated as the time at which 50% of cells have divided.

Lentiviral vector constructs and transduction.

Overexpression studies used a bidirectional MA1 vector (Amendola et al., 2005) in which transgene expression is driven by the SFFV promoter (van Galen et al., 2014b). CDK6 cDNA obtained from the Mammalian Gene Collection through PlasmID repository. Viral particles were produced as described (Mazurier et al., 2004) and were titrated on 293T human embryonic kidney cells. For transduction, $5x10^4$ $CD34^+$ $CD38^-$ cells, $5x10^3$ LT- or ST-HSCs purified by flow cytometry were incubated for 3–5 h in low cytokine condition medium. Cells were then incubated for 16-24 hours in the same medium supplemented with virus at a multiplicity of infection of 30–60 transforming units per mL.

Quantitative RT-PCR

RNA was extracted from 1×10^3 to 5×10^3 cells in TRIzol (Life Technologies) supplemented with 25 mg linear polyacrilamyde (LPA; Life Technologies) according to the manufacturer's protocol. Then, cDNA was reverse-transcribed with SuperScript VILO cDNA synthesis kit (Life Technologies) and was purified with QIAquick PCR

purification Kit (QIAGEN). Real-time PCR was done with SYBR Green PCR Master Mix (Applied Biosystems) and 200 nM primers (Qiagen) on an Applied Biosystems 7900HT instrument. *CDK6* and *GAPDH* primers used were Quantitect Primer Assays (Qiagen, *CDK6*: cat #QT00019985, *GAPDH*: cat #QT00079247); *ACTB* primers: forward, CCTGGCACCCAGCACAAT; reverse, GGGCCGGACTCGTCATAC. SDS software (Applied Biosystems) was used for absolute gene-expression quantification by the standard curve method. Two housekeeping genes (*ACTB* and *GAPDH*) were analyzed for comparison; data presented are relative to the geometric mean of expression of those two genes.

Mitochondrial mass measurements

Cord blood mononuclear cells were stained with CD34-APC-Cy7, CD38-PE-Cy7, CD90-PE, CD45RA-FITC, CD49f-PE, washed and then stained with Mitotracker Green FM (Life Technologies, M7514) for 30 minutes at 37°C at 1:40000 for analysis by flow cytometry.

Microarray profiling

RNA was extracted with TRizol (Life Technologies)- cDNA synthesis and preamplification were done as described (Fan et al., 2012). Samples were randomized on the microarray chips to minimize batch effects. Whole-genome gene-expression analysis was performed using the human Whole-Genome DASL HT-12 Assay (version 4.0) R2 (Illumina) (April et al., 2009).

Bioinformatics analysis.

Software of the R project for statistical computing (version 2.15.2) and Bioconductor (version 2.10) were used for all bioinformatics analyses. Quality control was performed using the *P*-detection values of <0.05, hierarchical clustering and PCA based on the Pearson correlation coefficient of all samples. Data were quantile-normalized ('normalizeQuantiles' command of limma software (Linear Models for Microarray Data; version 3.6.9), then were $\log_2$-transformed. All subsequent analyses used this data set. For the derivation of transcriptional programs, the STEM algorithm(Ernst et al., 2005) was used with the following parameters: a maximum of 50 model profiles, a maximum unit change between time points of 1 and a minimum correlation for clustering similar profiles >0.5. For gene-ontology enrichment with this software, *P* values were corrected with 500 randomizations and were considered significant with an FDR of <0.05. Genes differentially expressed between CB and any individual time point post-transplantation were identified with the limma package (version 3.6.9), which calculates the moderated *t*-test statistic for a particular contrast. All *t*-test scores were controlled for multiple-hypothesis testing with the Benjamini-Hochberg method, and genes with an adjusted *P* value of <0.05 were considered differentially expressed. To derive a core signature of gene expression differences between LT- and ST-HSCs in the time-course experiment, the BETR algorithm(Aryee et al., 2009) was used, with α set as 0.05. All genes with adjusted p.value < 0.05 were considered differentially expressed between LT- and ST-HSCs.

Derivation of a model describing the impact of G_0 exit delay on HSC pool dynamics.

Modeling was performed with a dynamic agent based model in a C++ custom code, compiled on g++ -std=c++11 -O3, g++ (SUSE Linux) 4.8.1 20130909 [gcc-4_8-branch revision 202388]. As the dynamics of the model essentially stem from delays, the system is formally of infinite order. It cannot therefore be expressed in differential form. The model consists of two modules: one that concerns the duration of the divisions (Fig.S6A – bottom panel), and one that concerns how the signals to divide are generated (Fig.S6A – top panel). Each cell in the HSC compartment considered is modeled individually: it has its own G_0 exit time and cell cycle transit time. The progenitor compartment is simplified: only the total number of cells in that compartment is tracked. The rules governing the life of each cell are: 1) Each cell has an individual timer. 2) When a new cell arises, it is given a G_0 exit time and cycle time randomly following a Gaussian distribution. The parameters of that Gaussian are given by the mean ± SD measured in the experiments (Fig. 2G-J; Fig.6A). 3) If a cell receives a signal for division for a duration equal or superior to its G_0 exit time, it divides. The signal also contains the information of which cell type is missing. The missing cell type information is averaged over the G_0 exit time. 4) The division gives rise to a new cell, the old one is kept. The nature of the new cell is determined randomly, according to the proportion received in the signal. For LT-HSCs, self-renewal gives rise to a LT-HSC, and asymmetric division gives rise to a new ST-HSCs. For ST-HSCs, self-renewal gives rise to a ST-HSC, and asymmetric division gives rise to a new progenitor. Symmetric division yielding to two progenitors does not occur in this model. 5) Upon division, the cell may die with a 5% probability (corresponding to the average death rate experimentally measured in the HSC compartment; data not shown). In this model, 3 types of signals are generated: 1) The number of progenitors is computed and the difference to the homeostatic number is

calculated. A noise is applied. 2) The number of ST-HSCs is computed and the difference to the homeostatic number is calculated. A noise is applied. 3) the number of LT-HSCs is computed and the difference to the homeostatic number is calculated. A noise is applied. At each time step of the simulation (2 minutes): 1) Cells increment their individual clocks. 2) If necessary, cells divide, self-renew, differentiate or die. 3) The progenitor pool loses a number of cells equal to the HSC exit rate. 4) Signals 1 and 2 are sent to the ST-HSC pool. Signals 2 and 3 to the LT-HSC pool. HSC pool exit occurs following a circadian rhythm where the frequency goes from 0 to double over the day. A control with no circadian rhythm was run in each case: no significant differences were observed. All runs were repeated 256 times, which was enough to reduce the standard error of all tracked quantities to 1% at most.

The model is based on the following assumptions and experimentally measured parameters: 1) The populations considered are hierarchically organized in the following order: LT-HSC, ST-HSC, progenitors. 2) Initially these populations contain a fixed number of cells at a ratio of 1:1:1000 for LT-HSC:ST-HSC:Progenitors which represents the experimental measure by flow cytometry and limiting dilution assays (data not shown). The simulations were run with initial populations set at 500 LT-HSCs, 500 ST-HSCs and 50000 Progenitors, which are also defined to be the target numbers to be maintained for homeostasis. 3) Each cell within the LT- and ST- HSC compartments is modeled to be in G_0, G_0 exit, or a cycling state. 4) Signals to divide or not divide are generated from the difference between the number of cells in a pool and the target number. 5) LT and ST HSCs always go back to G_0 when they divide. 6) There is no symmetrical division that produces 2 differentiated cells. 7) HSC death rate was defined by AnnexinV experimentally at

5% (data not shown). 8) There is noise in the system (the noise function is generated as a uniformly distributed random integer). The number of cells receiving the signal to exit G_0 is thus equal to the number of missing cells plus a randomly distributed uniform number representing noise, all other cells remain quiescent.

There are 2 parameters that cannot be measured experimentally and whose value may affect the outcomes of the simulations: 1) the HSC pool exit rate: this parameter determines how many cells get a signal to divide 2) different levels of noise. Therefore, in this implementation we tested all range of possible HSC pool exit rates: from 0 to 100% of compartment (Fig. S6B-E) as well as all reasonable noise values (Fig. S6F-G), to ensure our conclusions are robust. For values of HSC pool exit above 128 cells/day (25% of the pool), the number of progenitors cannot be maintained. By comparison, given our measured cycle times, a pool of 500 cells could give rise to a maximum of 177 cells/days if they divided continuously. The noise simulates variability in the mitogenic signals received. Indeed, it is unlikely that the number of missing cells be known to the unit, and, on the other hand, a signal containing no information cannot be used in a control loop.

There are 2 parameters that arise for any given HSC exit rate and noise value: 1) the self-renewal/differentiation ratio and 2) the number of divisions/year. This last parameter has been estimated experimentally in humans so we have chosen the combination of HSC pool exit rate (24 cells/day) and noise (5%) that outputs the number of divisions per year closest to the range reported in the literature (Catlin et al., Blood, 2011).

Two experiments were conducted: one at steady-state (homeostasis) and one where a perturbation is applied to simulate a stress/injury situation. The outcomes of maintaining a 5.8 hours G_0 exit delay in LT-HSCs compared to ST-HSCs (LT-HSC t_{G0exit} delay = 5.8h) were compared to a situation where there is no difference in G_0 exit duration between LT- and ST-HSCs (LT-HSC t_{G0exit} delay = 5.8h), or a situation in which HSCs are governed by a unique kinetic parameter (Fig.6A). The perturbation consisted in killing 10% of the progenitors at once, which subsequently requires all ST-HSCs to divide at least 10 times to return to the steady-state situation. As discussed above, simulations were run with 5 distinct HSC pool exit rates and 6 different noise parameters and all converged to the same conclusions. Five robust findings emerge. First, while the choice of the HSC pool exit rate and noise parameters affects the absolute values of the self-renewal/differentiation ratios, these are always higher for LT-HSCs, thus corroborating experimental data attributing the highest self-renewal capacity to LT-HSCs. This ratio is largely not influenced by the existence of a delay in G_0 exit in LT-HSCs (Fig.S6B-C). Second, in all the combinations of parameters tested, and maintaining a cell cycle transit time of LT-HSCs longer than that of ST-HSCs, the number of divisions in LT-HSCs was always minimized when LT-HSCs were set to have a delayed G_0 exit than ST-HSCs (Fig. 6B-C, Fig.S6D-G). This is an important property for the HSC pool as it is believed that LT-HSCs form a reservoir of pristine cells and each division is likely to accumulate damage. Third, we verified the dynamic range in which longer LT-HSC quiescence exit times may minimize LT-HSC divisions. Even if the LT-HSC G_0 exit delay is halved (2.9 hours), the number of LT-HSC divisions is still significantly lower than when quiescence exit times are not differentially maintained in LT- and ST-HSCs. Fourth, the progenitor population is maintained with similar efficiency

independent of whether the LT-HSCs cells have a longer t_{G0exit} than ST-HSCs (Fig.S6H). Fifth, after perturbation, the return to steady-state is faster when the LT-HSCs are delayed in their G_0 exit (Fig. 6H, Fig.S6H-I). In the case in which there is no delay in G_0 exit times in LT-HSC compared to ST-HSC, spikes in the number of cycling cells mean that the system at that point is less responsive to further perturbations (Fig.S6I). This last finding indicates that the control of the number of downstream cells is overall more robust when the G_0 exit times differ between LT- and ST-HSCs. Collectively, additional differential control of the delay in G_0 exit of LT-HSCs further limits LT-HSC division and increases the overall robustness of the system. Differential regulation of G_0 exit duration in the HSC hierarchy is thus key to maintain the integrity of the HSC pool.

Supplemental References

Amendola, M., Venneri, M.A., Biffi, A., Vigna, E., and Naldini, L. (2005). Coordinate dual-gene transgenesis by lentiviral vectors carrying synthetic bidirectional promoters. Nat. Biotechnol. *23*, 108–116.

April, C., Klotzle, B., Royce, T., Wickham-Garcia, E., Boyaniwsky, T., Izzo, J., Cox, D., Jones, W., Rubio, R., Holton, K., et al. (2009). Whole-genome gene expression profiling of formalin-fixed, paraffin-embedded tissue samples. PloS One *4*, e8162.

Aryee, M.J., Gutiérrez-Pabello, J.A., Kramnik, I., Maiti, T., and Quackenbush, J. (2009). An improved empirical bayes approach to estimating differential gene expression in microarray time-course data: BETR (Bayesian Estimation of Temporal Regulation). BMC Bioinformatics *10*, 409.

Catlin, S.N., Busque, L., Gale, R.E., Guttorp, P., and Abkowitz, J.L. (2011). The replication rate of human hematopoietic stem cells in vivo. Blood *117*, 4460–4466.

Ernst, J., Nau, G.J., and Bar-Joseph, Z. (2005). Clustering short time series gene expression data. Bioinforma. Oxf. Engl. *21 Suppl 1*, i159–i168.

Fan, J.-B., Chen, J., April, C.S., Fisher, J.S., Klotzle, B., Bibikova, M., Kaper, F., Ronaghi, M., Linnarsson, S., Ota, T., et al. (2012). Highly Parallel Genome-Wide Expression Analysis of Single Mammalian Cells. PLoS ONE *7*, e30794.

Van Galen, P., Kreso, A., Wienholds, E., Laurenti, E., Eppert, K., Lechman, E.R., Mbong, N., Hermans, K., Dobson, S., April, C., et al. (2014). Reduced Lymphoid

Lineage Priming Promotes Human Hematopoietic Stem Cell Expansion. Cell Stem Cell *14*, 94–106.

Mazurier, F., Gan, O.I., McKenzie, J.L., Doedens, M., and Dick, J.E. (2004). Lentivector-mediated clonal tracking reveals intrinsic heterogeneity in the human hematopoietic stem cell compartment and culture-induced stem cell impairment. Blood *103*, 545–552.
