# Supplementary material for: CDK6 Levels Regulate Quiescence Exit in Human Hematopoietic Stem Cells
Source: Cell Stem Cell. 2015 Mar 5;16(3):302–13. doi: 10.1016/j.stem.2015.01.017 (PMC4359055; doi:10.1016/j.stem.2015.01.017)
Supplement: Document S2. Article plus Supplemental Information [file mmc3.pdf]

Article

Cell Stem Cell

CDK6 Levels Regulate Quiescence Exit in Human Hematopoietic Stem Cells

Graphical Abstract

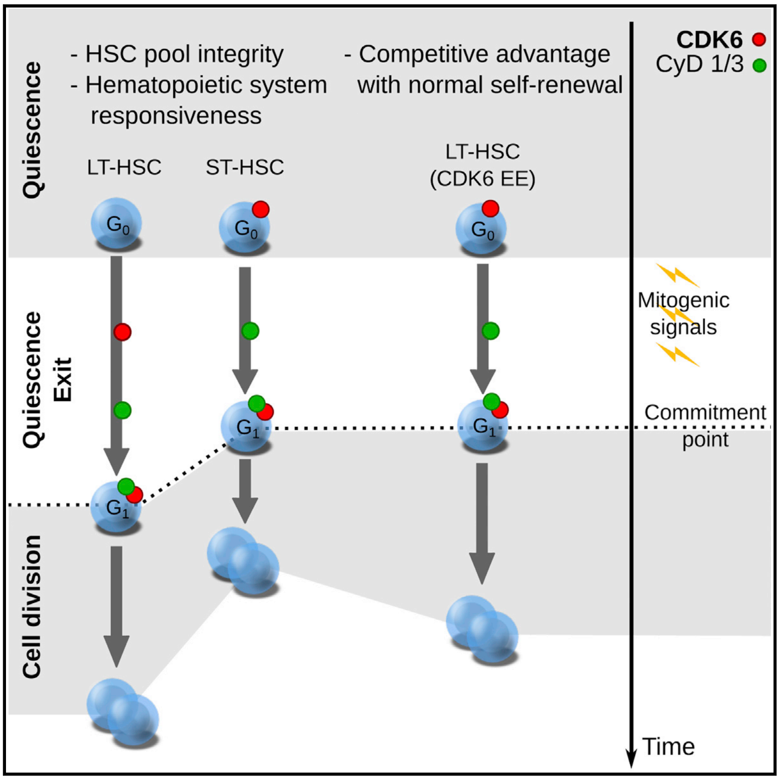

Highlights

- Human long-term (LT) and short-term (ST) HSCs are equally quiescent
- LT- and ST-HSCs differ in division kinetics and expression of CDK6
- CDK6 expression regulates the timing of exit from quiescence
- Differential regulation of quiescence helps maintain hematopoiesis

Authors

Elisa Laurenti, Catherine Frelin, ..., Norman Iscove, John E. Dick

Correspondence

el422@cam.ac.uk (E.L.), jdick@uhnresearch.ca (J.E.D.)

In Brief

The hematopoietic stem cell (HSC) compartment is heterogeneous in terms of cell cycle properties. Laurenti et al. show that the timing of exit from quiescence in human HSC subsets is controlled by CDK6 expression levels. This differential control has an impact on the long-term preservation of the HSC pool.

Accession Numbers

GSE58299

Laurenti et al., 2015, Cell Stem Cell *16*, 302–313
March 5, 2015 ©2015 The Authors
http://dx.doi.org/10.1016/j.stem.2015.01.017

CellPress

CDK6 Levels Regulate Quiescence Exit in Human Hematopoietic Stem Cells

Elisa Laurenti,[1,8,9,*] Catherine Frelin,[1,8] Stephanie Xie,[1,8] Robin Ferrari,[1,3] Cyrille F. Dunant,[4] Sasan Zandi,[1] Andrea Neumann,[1] Ian Plumb,[1] Sergei Doulatov,[5] Jing Chen,[6] Craig April,[6] Jian-Bing Fan,[6] Norman Iscove,[1,7] and John E. Dick[1,2,*]
[1]Princess Margaret Cancer Centre, University Health Network, Toronto, ON M5G 1L7, Canada
[2]Department of Molecular Genetics, University of Toronto, Toronto, ON M5S 1A8, Canada
[3]Ecole Normale Supérieure de Cachan, Département de Biologie, Cachan, 94235, France
[4]Ecole Polytechnique Fédérale de Lausanne, LMC, Station 12, Lausanne, CH-1015, Switzerland
[5]Division of Pediatric Hematology/Oncology, Boston Children's Hospital and Harvard Medical School, Harvard Stem Cell Institute, Boston, MA 02115, USA
[6]Illumina, San Diego, CA 92121, USA
[7]Department of Medical Biophysics, University of Toronto, Toronto, ON M5G 1L7, Canada
[8]Co-first author
[9]Present address: Wellcome Trust – Medical Research Council Cambridge Stem Cell Institute, Department of Haematology, University of Cambridge, Cambridge, CB2 0AH, UK
*Correspondence: el422@cam.ac.uk (E.L.), jdick@uhnresearch.ca (J.E.D.)
http://dx.doi.org/10.1016/j.stem.2015.01.017
This is an open access article under the CC BY license (http://creativecommons.org/licenses/by/4.0/).

SUMMARY

Regulated blood production is achieved through the hierarchical organization of dormant hematopoietic stem cell (HSC) subsets that differ in self-renewal potential and division frequency, with long-term (LT)-HSCs dividing the least. The molecular mechanisms underlying this variability in HSC division kinetics are unknown. We report here that quiescence exit kinetics are differentially regulated within human HSC subsets through the expression level of CDK6. LT-HSCs lack CDK6 protein. Short-term (ST)-HSCs are also quiescent but contain high CDK6 protein levels that permit rapid cell cycle entry upon mitogenic stimulation. Enforced CDK6 expression in LT-HSCs shortens quiescence exit and confers competitive advantage without impacting function. Computational modeling suggests that this independent control of quiescence exit kinetics inherently limits LT-HSC divisions and preserves the HSC pool to ensure lifelong hematopoiesis. Thus, differential expression of CDK6 underlies heterogeneity in stem cell quiescence states that functionally regulates this highly regenerative system.

INTRODUCTION

Hematopoiesis ensures that blood demand is met under homeostatic and stress conditions through tightly controlled regulation of hematopoietic stem cells (HSCs) and their progeny. HSCs are historically identified by the unique capacity to self-renew, providing long-term, serial reconstitution of the entire hematopoietic system upon their transplantation into myeloablated hosts. Functional self-renewal of HSCs is associated with reduced cell cycle activity. Seminal papers demonstrated that cell cycle becomes more frequent as HSCs gradually differentiate into lineage-restricted progenitors (Bradford et al., 1997; Morrison and Weissman, 1994; Pietrzyk et al., 1985; Suda et al., 1983; Uchida et al., 2003). Although the HSC compartment was thought to be heterogeneous in cycling ability (Micklem and Ogden, 1976) 40 years ago, this has only recently been supported by experimental evidence as follows. (1) Label retaining studies (Foudi et al., 2009; Qiu et al., 2014; Takizawa et al., 2011; Wilson et al., 2008) conclusively established that the HSC pool comprises at least two compartments differing in their frequency of division. (2) The most dormant cells have the highest repopulation capacity and can be reversibly brought into cell cycle through extrinsic cues, especially upon injury (Foudi et al., 2009; Wilson et al., 2008). (3) The HSC pool has been fractionated into long-term (LT-), intermediate-term (IT-), short-term (ST-) HSCs and multipotent progenitors (MPPs) and is hierarchically organized based on progressively reduced repopulation capacity and increased cycling properties (Benveniste et al., 2010; Cheshier et al., 1999; Copley et al., 2012; Foudi et al., 2009; Oguro et al., 2013; Passegué et al., 2005; Qiu et al., 2014; Wilson et al., 2008). While the hierarchically organized HSC subsets are widely thought to prevent HSCs exhaustion and preserve lifelong blood production, knowledge of the molecular mechanisms that govern the variable cycling properties of each HSC subset is lacking.

Quiescence, defined as a reversible absence of cycling, also called G_0, is a defining feature of HSCs first described in Lajtha (1963). Most transgenic and knockout mouse models altering HSC function decrease quiescence, leading to HSC exhaustion (reviewed in Pietras et al., 2011; Rossi et al., 2012). Quiescence and infrequent cycling of HSCs are considered to protect against damage accumulation, and impaired maintenance of HSC quiescence is thought to contribute to aging and leukemia. However, understanding how HSCs switch from quiescence to

cycling and how division, self-renewal, and differentiation are integrated is lacking.

Upon reception of mitogenic signals, multiple processes must occur: HSCs must exit quiescence to enter the cell cycle, which then must be traversed to complete a division. This requires reactivating all the necessary metabolic and cell cycle machinery. Doubling time analysis at homeostasis has shown that ST-HSCs and MPPs divide more frequently than LT-HSCs (Foudi et al., 2009; Oguro et al., 2013; Wilson et al., 2008). Little is known about quiescence exit. It is unclear if and how it is differentially regulated among distinct HSC subsets and if the duration of this exit affects HSC function. We recently showed that the duration of a division starting from G_0 after stimulation by a mitogenic signal is shorter in IT-HSCs than in LT-HSCs (Benveniste et al., 2010). The unknown mechanism underlying increased cycling in IT/ST-HSCs could theoretically be due to (1) easier activation from external stimuli, (2) less time in G_0, (3) faster exit from quiescence, (4) faster completion of divisions, or (5) a combination of these. An integrated view is necessary to ascertain how these properties in HSC subsets are molecularly regulated. Here, we establish that the duration of HSC exit from quiescence upon mitogenic stimulation is differentially regulated within the human HSC pool by a CDK6-primed quiescence state in ST-HSCs. Tight control of quiescence exit length via CDK6 levels plays an important role in HSC pool dynamics, preserving integrity and preventing LT-HSCs clonal expansion.

RESULTS

Heterogeneity in the Human HSC Pool

The cycling properties of mouse HSC subpopulations are described, but they have not been validated in the human HSC hierarchy. Human LT-HSCs, isolated from umbilical cord blood (CB) as Lin^- $CD34^+$ $CD38^-$ $CD45RA^-$ $CD90^+$ $CD49f^+$ (Notta et al., 2011), provide robust multilineage repopulation beyond 30 weeks in the NSG mouse xenograft assay with about 10% frequency (Notta et al., 2011) and efficiently engraft upon secondary transplantation (Table S1). In contrast, Lin^- $CD34^+$ $CD38^-$ $CD45RA^-$ $CD90^-$ $CD49f^-$ cells generate multilineage grafts over intermediate time periods (Notta et al., 2011), but they lack serial transplantation ability and thus have limited self-renewal (Table S1). According to the criteria used in mouse, this population corresponds to ST-HSCs. Importantly, LT- and ST-HSC-enriched populations can be purified with the cell surface markers indicated above from NSG mice repopulated with human cells (Table S1). Similar to transplantation models in mice, phenotypic human LT- and ST-HSCs expand in the first 4 weeks after xenotransplant (with >50% that actively cycle) then regain quiescence by 20 weeks when a transient equilibrium phase is reached (Figures 1A, S1A, and S1B). In our model, functionally repopulating LT-HSCs expanded ≈30-fold in 20 weeks (Figure 1B).

To estimate the division frequency of human HSC subsets, we tracked BrdU incorporation kinetics of phenotypic LT- and ST-HSCs in xenografts and observed, similarly to mouse models, that phenotypic LT-HSCs divide less frequently (1.5- to 1.9-fold) than phenotypic ST-HSCs (Figure 1C), whether in expansion or at equilibrium. Although both subsets highly proliferate after transplantation, we detected fewer ST-HSCs, possibly because of their higher drive to produce differentiated cells (Figure 1A). To capture a core signature of genes distinguishing LT- from ST-HSCs, we subjected LT- and ST-HSCs isolated from CB and at different times after xenotransplantation to transcriptome analysis. Using the Bayesian Estimation of Temporal Regulation algorithm (Aryee et al., 2009), we identified 241 genes showing sustained differential expression between LT- and ST-HSCs independent of environmental effects and changes in proliferation (Table S2 and Figures S1C–S1E). This was significantly more than in our previous static analyses of CB (Laurenti et al., 2013; Notta et al., 2011). This signature contains genes important in murine HSC function (Gazit et al., 2013; Jankovic et al., 2007; Kataoka et al., 2011; Laurenti et al., 2008) (Figure 1D) and is enriched for gene ontology terms related to the immune/inflammatory response, chromatin remodeling, and most significantly, cell cycle regulation (Figure 1E). Thus, human ST-HSCs, similar to mouse, have lower repopulation capacity, more frequent divisions, and a distinct transcriptional profile compared to LT-HSCs.

LT-HSCs and ST-HSCs Are Equally Quiescent

The increased frequency of ST-HSC divisions may be due to (1) more cells actively cycling at any time, (2) increased sensitivity, or (3) faster response to mitogenic stimulation. To resolve the basis for these increased divisions, we investigated the proportions of LT- and ST-HSCs in each cell cycle phase and found them to be identical at all points during the xenotransplantation process (Figure S1B). Freshly isolated from CB, both HSC subsets had more than 90% of cells in G_0 ($Ki67^-$ 2n DNA content, Figures 2A and 2B). Importantly, no cell was found in S-G_2-M as determined by DNA content (Figures 2A and 2B) and by complete absence of the mitotic marker phosphoH3 (Figure S2A). Cell diameters were equally small in LT- and ST-HSCs (Figure 2C), with both lacking in cytoplasm. Metabolically, both LT- and ST-HSCs showed low mitochondrial activity (Figure 2D) and similar levels of mTOR activation (assessed by phosphoS6 staining; Figure 2E). All these parameters indicate a G_0 quiescent state. To exclude a possible differential G_1 arrest state for LT- and ST-HSCs, we analyzed the phosphorylation state of retinoblastoma protein (RB) at S807/S811, a marker upregulated in G_0 cells before entry into G_1 (Ren and Rollins, 2004). Both cell types were negative (Figures 2F and S2B). In contrast, granulocyte-monocyte progenitors (GMPs) were largely in G_1, as most cells were $Ki67^+$ with 2n DNA content (Figure 2B) and had a larger diameter (Figure 2C), visible cytoplasm, increased mitochondrial activity (Figure 2D), and RB phosphorylation on S807/S811 (Figure 2F). Collectively, these data establish that both human LT- and ST-HSCs freshly isolated from CB reside in a G_0 quiescent state lacking all markers of G_1.

Distinct Cell Division Durations in HSC Subsets

Since the proportions of LT- and ST-HSCs in G_0 were identical (Figures 2B and S1B), we hypothesized that the differences in expression of cell cycle genes and frequency of division observed between these two subsets reflected differences in their capacity to exit quiescence upon mitogenic stimulation. Therefore, we measured the duration of single divisions occurring upon activation by a mitogenic signal. Such studies need to be performed with single cells using in vitro assays. We sorted 576

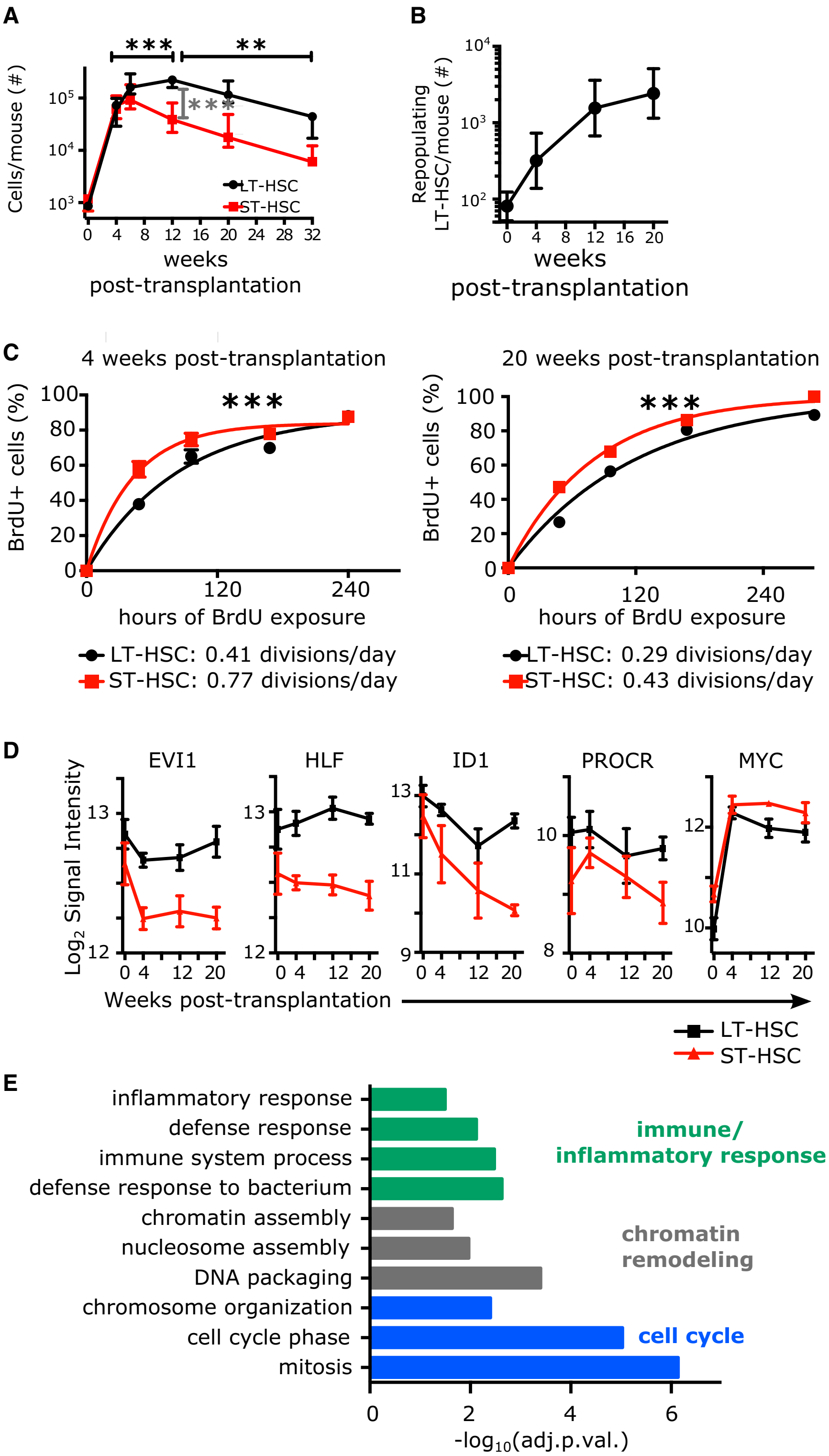

Figure 1. Human HSC Subsets in the Xenograft Divide with Distinct Frequencies and Display Distinct Transcriptional Profiles

(A) Number of cells per mouse of indicated populations in the bone marrow of the mice at indicated time points post-transplantation of 70,000 Lin^- CB (saturating number of LT-HSCs). Median and interquantile ranges are shown. ***$p < 0.01$ by one-way ANOVA and Tukey test.

(B) The number of repopulating LT-HSCs per mouse at indicated time points post-transplantation were calculated by multiplying the number of phenotypic LT-HSCs shown in (A) by the frequency of long-term repopulating cells indicated in Table S1.

(C) BrdU incorporation kinetics over 12 days of LT-HSC (black) and ST-HSC (red) enriched populations isolated from pools of two to five mice engrafted with 70,000 Lin^- CB cells. BrdU was started either at 4 (left panel, expanding phase) or 20 weeks post-transplantation (right panel, equilibrium phase). n = 1–4 pools of three to five mice from six (4 weeks) or one (20 weeks) independent CB samples. Curve is least-squares fit. Left panel: $R^2 > 0.96$; right panel: $R^2 > 0.98$. Doubling times (half times of fit) in hours are shown in the insert. ***$p < 0.01$ by extra-sum of squares test.

(D and E) Derivation of a 241-gene signature distinguishing LT- and ST-HSCs in unperturbed CB over 20 weeks in a xenotransplant. (D) Examples of five expression profiles of genes with known HSC function over the course of 20 weeks of xenotransplant (black: LT-HSCs, red: ST-HSCs), mean ± S.E.M shown, n = 3 per time point. (E) Selected gene ontology terms significantly enriched in the 241-gene LT-HSC/ST-HSC core signature. Shown is the $-\log_{10}$ of the Benjamini-Hochberg adjusted p value.

See also Figure S1.

single LT- and ST-HSCs from CB and monitored their divisions over 140 hr in serum-free conditions. As expected, proliferation was higher in ST-HSCs (Figure S2C). The mean time to first division ($t_{FirstDiv}$) varied between CB samples, but on average it was 9 hr shorter in ST-HSCs compared to LT-HSCs (Figures 2G and 2H). The mean time to second division ($t_{SecondDiv} = t_{G1\text{-}S\text{-}G2\text{-}M}$) was also significantly shorter in ST-HSCs than in LT-HSCs (Figures 2I and S2D). Importantly the second division was always shorter than the first, identifying a latency restricted to HSCs that transition out from a non-stimulated quiescent state. In contrast to in vivo repopulation, cells in this assay do not return to G_0 after division (Figure S2E). Therefore, the latency phenomenon observed in the first division encompasses the events pertaining to the G_0 to G_1 transition but may also include portions of early G_1. For simplicity, it will be hereafter called "G_0 exit," and calculated as $t_{FirstDiv} - t_{SecondDiv}$. By this analysis, LT-HSCs egressed from G_0 less rapidly than ST-HSCs on average by 5.8 hr (Figure 2J). Similar results with slower kinetics were obtained when single LT- and ST-HSCs were cultured in a medium with lower cytokine and nutrient concentrations (Figures S2F and S2G). These parameters are not unique to

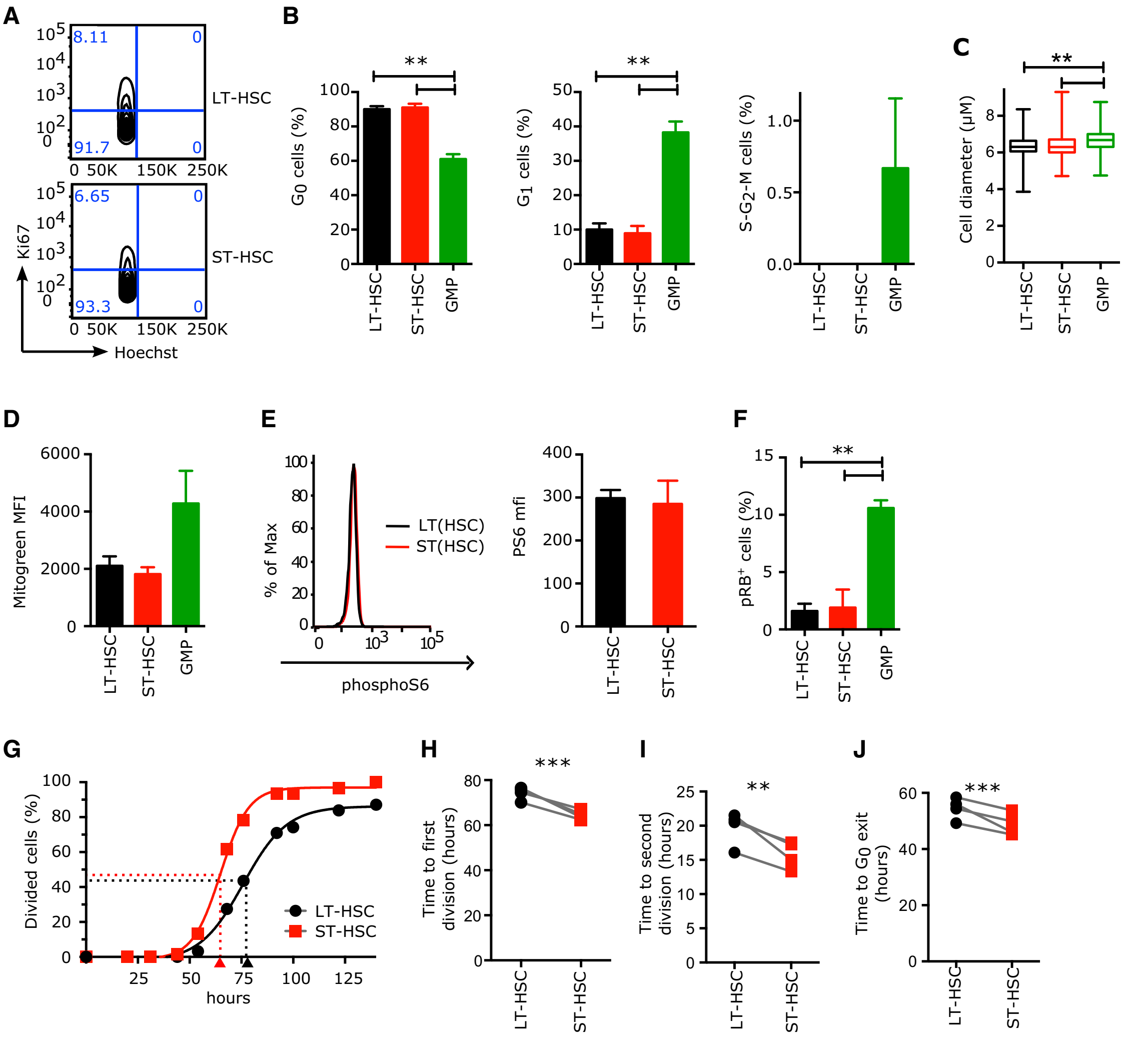

Figure 2. LT- and ST-HSCs Are Equally Quiescent, but upon Mitogenic Stimulation They Differ in the Duration of Divisions Starting from G_0 or G_1

(A and B) Proportion of human CB HSC and progenitor cells in each phase of the cell cycle. Parameters were assessed by flow cytometry using Ki67 and Hoechst ($Ki67^-$ 2n DNA content, G_0; $Ki67^+$ 2n DNA content, G_1; $Ki67^+$ > 2n DNA content, S-G_2-M). (A) Representative flow cytometry cell cycle profiles of CB LT- and ST-HSCs and the percentage of cells in each gate. Event count: LT-HSCs (top panel), 1,320 cells; ST-HSCs (bottom panel), 1,143 cells. (B) Mean ± SEM is shown; n = 3 CB samples.

(C) Cell diameter of indicated populations measured with ImageJ from microscopy pictures. n > 323 cells from four independent CB samples.

(D) Mitochondrial mass as measured by flow cytometry with MitoGreen. MFI, Mean Fluorescence Intensity; mean ± S.E.M shown, n = 2 independent CB samples.

(E) PhosphoS6 protein levels as measured by flow cytometry. Left panels: representative flow cytometry plots; black line, LT-HSCs; red line, ST-HSCs. Right panel: median fluorescence intensity of phosphoS6 staining. Mean ± SEM is shown. n = 2 CB samples.

(F) Percentage of cells positive for phosphoRB (S807/S811) as measured by flow cytometry; mean ± S.E.M shown, n = 2 independent CB samples. GMP, granulocyte-monocyte progenitors.

(G) Cumulative first division kinetics (excluding dead cells) of LT-HSCs (black) and ST-HSCs (red) from a representative CB example. Curve is least-squares sigmoid fit. $R^2 > 0.99$. Arrowheads represent time to first division as estimated from sigmoid fit ($t_{FirstDiv} = logEC_{50}$). Time 0 is the time of exposure to mitogenic stimulus.

(H) Mean time to first division (in hours).

(I) Mean time of cell cycle transit ($t_{SecondDiv} = logEC_{50}$ of sigmoid fit of cumulative second division kinetics; see Figure S2E).

(J) Mean time of G_0 exit (in hours) ($t_{G0exit} = t_{FirstDiv} - t_{SecondDiv}$).

In (H)–(J), individual CB samples are shown; gray lines connect LT-HSC and ST-HSC parameters from the same CB. **p < 0.05, ***p < 0.01 by paired t test. See also Figure S2.

CB: fully quiescent LT-HSCs isolated from adult bone marrow also displayed a significant delay in G_0 exit compared to ST-HSCs (Figure S2H). Collectively, upon stimulation by mitogenic signals, the duration of a division is consistently shorter in human ST-HSCs than in LT-HSCs, whether cells need to transition out of quiescence or continuously cycle.

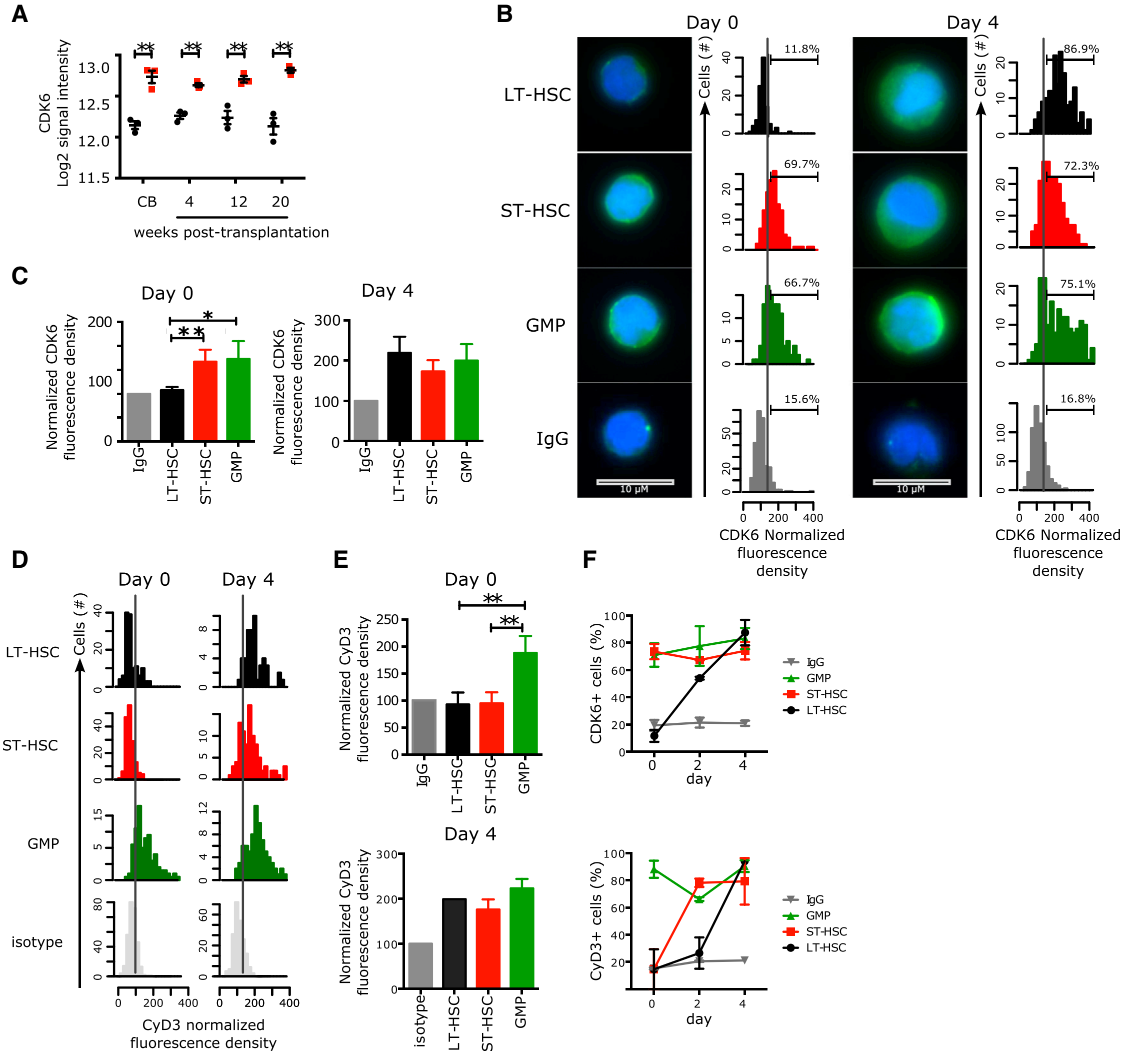

Figure 3. Distinct CDK6 Levels Govern the G_0 Exit Kinetics of LT- and ST-HSCs

(A) Log2 signal intensity for *CDK6* mRNA probe. Shown are individual measures (black circles: LT-HSCs, red squares: ST-HSCs, green triangles: GMPs) and the median and interquantile ranges (horizontal bars); n = 3. All multiple comparisons have been tested.

(B) Immunofluorescence for CDK6 protein in LT- and ST-HSCs sorted from CB (left panel) or cultured for 4 days (right panel). Representative pictures and histograms of CDK6 fluorescence density are normalized to the fluorescence density of the IgG control in the same population. Positivity threshold was set over the median + 1 SD of the IgG control distribution and the percetnage of positive cells is indicated. 100–570 cells are analyzed with n = 3 CB samples. Scale bar represents 10 μM.

(C) Normalized median CDK6 fluorescence density. Mean ± SEM is shown; n = 3 CB samples. GMPs, granulocyte-monocyte progenitors.

(D) Immunofluorescence for CyclinD3 protein in LT-HSCs, ST-HSCs, and GMPs from freshly isolated CB (Day 0, left panel) or after 4 days of culture (Day 4, right panel). Shown are histograms of CyclinD3 fluorescence density normalized to the fluorescence density of the IgG control in the same population. Positivity threshold (dotted line) was set over the median + 1 SD of the IgG control distribution. n = 135–315 cells analyzed for day 0 and n = 49–245 cells for day 4.

(E) Normalized median CyclinD3 fluorescence density at the indicated time points. Mean ± SEM is shown; n = 3 CB samples. **$p < 0.05$ by paired t test.

(F) Time course analysis of CDK6 and CyclinD3 upon stimulation by mitogenic signals. Percentages of $CDK6^+$ (top panel) or $CyclinD3^+$ (bottom panel) cells in each of the indicated populations at the indicated time points after isolation from CB are shown. Mean ± S.E.M shown. n = 3 CB samples, except for day 2, where n = 2 CB samples. **$p < 0.05$ by paired t test.

See also Figure S3.

Distinct Expression of CDK6 Protein in the Quiescent HSC Pool

To identify the molecular determinants underlying differences in the duration of LT- and ST-HSC divisions, we screened the 241-gene core signature for genes known to be involved in either G_0 exit or G_1 progression. *CDK6* was selected because its mRNA was consistently upregulated in ST-HSCs both in CB and upon xenotransplantation (Figures 3A, S3A, and S3B), and CDK6/CyclinD complexes regulate G_0 exit and early G_1 (Sherr and Roberts, 2004). Importantly, the CDK6 protein was undetectable in most of the quiescent CB LT-HSCs, but it was upregulated after 4 days of culture when all HSC subsets actively cycle (Figures 3B

and 3C). In sharp contrast, before culture, ST-HSCs already expressed high levels of CDK6 protein, similar to that found in G_1 GMPs, despite being quiescent (Figures 3B, 3C, and S3C). Similarly, adult ST-HSCs isolated from either bone marrow or mobilized peripheral blood also expressed high levels of CDK6, though it was undetectable in adult LT-HSCs (Figures S3D–S3F). To gain insight into how ST-HSCs can remain quiescent with high levels of CDK6 protein, and because CDK6 kinase activity depends on its association with CyclinD proteins, we examined the levels of CyclinD1 and CyclinD3 protein in sorted LT- and ST-HSC subsets from purified CB at day 0. We found that neither one expressed CyclinD1 nor CyclinD3; as expected, both were expressed in G_1 GMPs (Figures S3G, S3H, 3D, and 3E). Therefore the CDK6 in ST-HSCs is not part of an active complex, which explains the absence of RB phosphorylation in these cells (Figure 2F) and their quiescence. To gain insight into how the CyclinD-CDK6 complexes integrate proliferative signals once HSCs are activated, we did a time course analysis of CDK6, CyclinD1, and CyclinD3 protein expression. After 2 days of culture, less than 5% of LT- and ST-HSCs divided (Figure 2G). Interestingly, about 54% of LT-HSCs expressed CDK6 and 44% and 25% express CyclinD1 and CyclinD3, respectively (Figures S3H and 3F). In contrast, almost all ST-HSCs had upregulated CyclinD3 protein by day 2, and 52% express CyclinD1. By day 4, when all LT- and ST-HSCs actively cycle (Figure 2G), each HSC subset expresses both CDK6 and CyclinD3 proteins (Figure 3F). These data indicate that, upon activation by mitogens, the assembly of the CDK/CyclinD complex is more rapid and more robust in ST-HSCs than in LT-HSCs. Overall, these data reveal two unexpected findings. First, ST-HSCs exist in a G_0 state, yet they express a known driver of G_1 progression (CDK6) while lacking the cognate partners (CyclinD1 and CyclinD3) of CDK6. Second, the hierarchical organization based on functional repopulation properties also exhibits a hierarchy of CDK6/CyclinD complex components reflecting distinct cycling properties: LT-HSCs are negative for both CDK6 and CyclinD; ST-HSCs express exclusively CDK6; and lineage-restricted progenitors, e.g., GMPs, express both.

CDK6 Levels Regulate the Duration of G_0 Exit

To gain mechanistic insight into the correlation between CDK6 protein levels and cell division duration within HSC subsets, we altered CDK6 levels and investigated the effect on the kinetics of the first HSC division. To examine loss of function, we measured the duration of cell division when single LT- and ST-HSCs are exposed to a mitogenic stimulus in the presence of the highly specific CDK4-CDK6 inhibitor PD033299. The majority of LT-HSCs never divided in the presence of PD033299 (Figure 4A). Similarly there was a strong reduction in the number of ST-HSCs that could divide (Figure 4A). However, for those ST-HSCs that divided, inhibition of CDK6 brought the length of the first division to that of LT-HSCs (Figures 4B and 4C). Intriguingly, those 10% LT-HSCs dividing in the presence of PD033299 were not further delayed, potentially representing a subset of more “activated” cells within the LT-HSC phenotypic compartment. To examine the consequences of CDK6 gain of function, we enforced expression of CDK6 protein (CDK6 EE) with lentiviral vectors in LT- and ST-HSCs before their first division (Figures S4A and S4B). CDK6 EE did not change any ST-HSC cell cycle parameters (Figures 4D–4F, S4C, and S4E). By contrast, a division starting from G_0 (first division) of CDK6 EE LT-HSCs was significantly shortened to values similar to those of ST-HSCs (Figures 4D and 4E); control transduced LT-HSCs (LUC) showed no such changes. CDK6 EE did not decrease the duration of a division starting from G_1 that remained significantly longer than that of ST-HSCs (Figures 4F, S4C, and S4F). Also, CDK6 EE did not affect the long-term proliferative output of LT-HSCs in vitro in conditions where cells do not return to G_0 (Figure 4G). These approaches show that CDK6 shortens divisions starting from G_0, but not divisions starting from G_1. Moreover, variation in CDK6 protein levels between HSC subsets results in active regulation of the duration of the latency that is unique to HSCs transiting out of G_0, a process we define as G_0 exit. Importantly, our data also establish that pre-existent CDK6 in ST-HSCs primes them for earlier cell division upon mitogenic stimuli.

CDK6 EE Confers a Competitive Advantage to LT-HSCs without Exhaustion

In mouse models, failure to maintain quiescence and/or increased cycling are mostly associated with decreased self-renewal and eventual HSC exhaustion (Orford and Scadden, 2008; Pietras et al., 2011; Rossi et al., 2012). To examine the long-term effect of exclusively accelerating the duration of exit from quiescence upon reception of mitogenic stimuli, we enforced CDK6 expression in LT-HSCs in vivo, where HSCs return to G_0 after most divisions under homeostatic conditions (Wilson et al., 2008 and Figure S1B). Competitive xenotransplantation experiments showed that CDK6 EE does not confer a proliferative advantage within the first 4 weeks post-transplantation, during which HSCs are actively cycling (Figure 5A). However, by 20 weeks post-transplantation, most HSCs have regained quiescence. At this point, CDK6 EE cells, unlike LUC cells, significantly outcompete untransduced cells (median GFP^+ percentage: LUC, 59.4%; CDK6 EE, 76.2%, $p = 0.007$, Figure 5A) without displaying any lineage bias (Figures 5B and S5A). This expansion originated from LT-HSCs (Figures 5C and 5D) and extended to all progenitor populations (Figure S5B). To determine whether the CDK6 EE in phenotypic LT-HSCs altered serial repopulating capacity, secondary transplantation was performed. There was no significant difference in the graft size at 12 weeks after secondary transplantation when high numbers of control (LUC/GFP^-) and CDK6 EE LT-HSCs were transplanted (Figures 5E and S5C). However, limiting dilution analysis revealed a 4-fold increase in the frequency of repopulating LT-HSCs in the CDK6 EE group compared to controls (Figure 5F), confirming LT-HSC expansion over two rounds of transplantation. Importantly, like our in vitro results, CDK6 EE did not change the rate of cell cycle transit of LT- or ST-HSCs (Figure S5D) but accelerated the first division of LT-HSCs (Figure S5E). These data show that the unique shortening in the duration of G_0 exit conferred by CDK6 EE gives LT-HSCs a competitive advantage without altering self-renewal or differentiation abilities.

Simulating the Impact of G_0 Exit Durations on Hematopoietic Homeostasis

Our data show that upon activation, LT-HSCs are delayed in their quiescence exit. Because LT-HSCs have been estimated to divide very infrequently, approximately once every 135 and

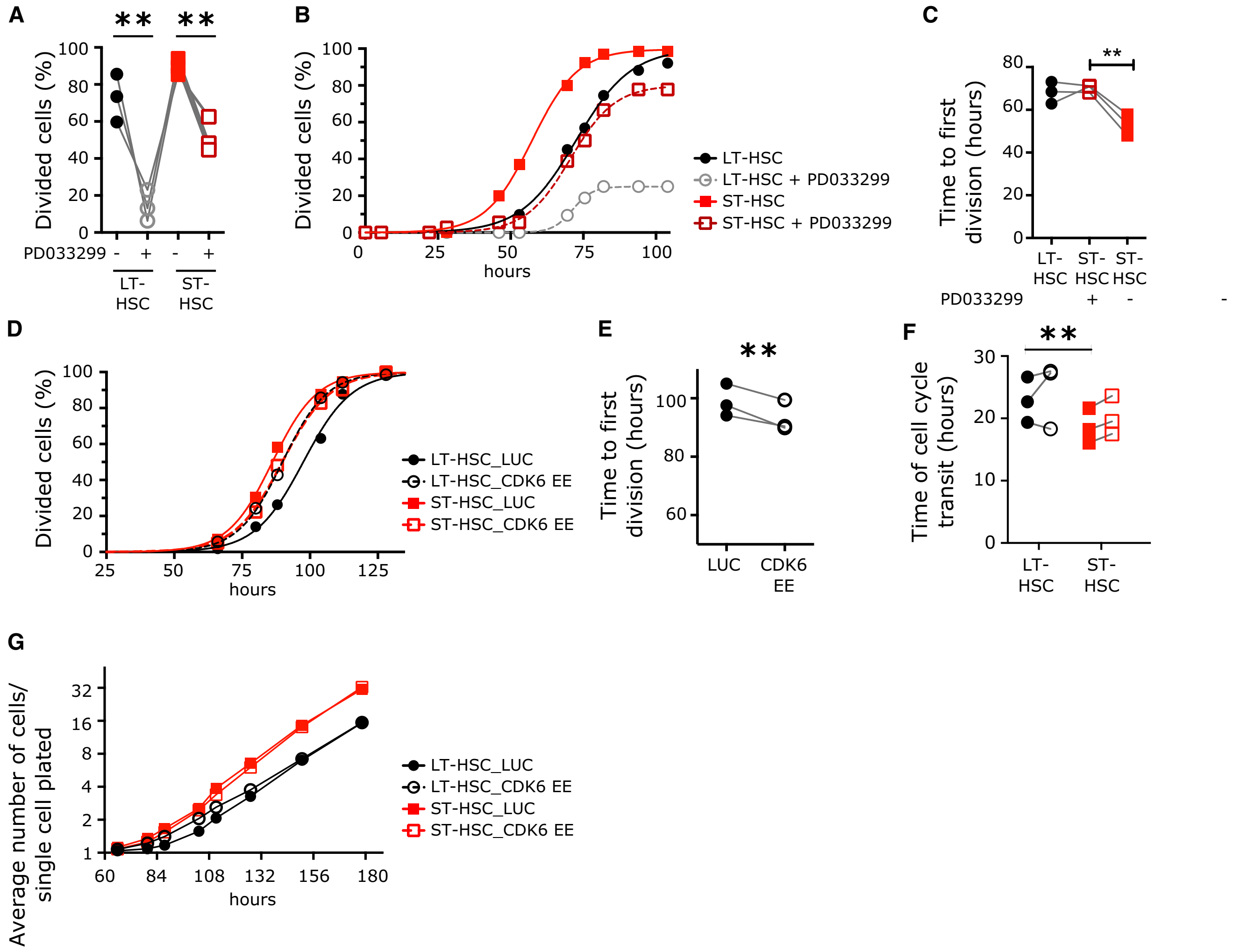

Figure 4. CDK6 Levels Determine the Duration of Quiescence Exit in the HSC Pool

(A–C) Cell division duration of single LT- and ST-HSCs after exposure to mitogenic signal in the presence or absence of PD033299 (50 nM). (A) Percentage of cells from the indicated populations that divided after 100 hr in culture. (B) Cumulative first division kinetics (excluding dead cells). Data from a representative CB example are shown. Curve is least-squares sigmoid fit. $R^2 > 0.99$. (C) Mean time to first division (hours) ($t_{firstDiv} = logEC_{50}$).

(D–G) Cell division duration of single LT- and ST-HSCs after exposure to mitogenic signal with or without CDK6 EE. (D) Cumulative first division kinetics (excluding dead cells) of indicated populations transduced with indicated lentiviral vectors. Data from a representative CB are shown. Curve is least-squares sigmoid fit. $R^2 > 0.99$. (E) Mean time to first division (hours) ($t_{firstDiv} = logEC_{50}$). (F) Time of cell cycle transit of indicated populations in hours.

(G) Expansion curves of LT- and ST-HSCs in culture. Shown is the average number of cells per single cell plated at the indicated time points after culture initiation. Data are from one representative experiment out of three. Time 0 represents the time of exposure to mitogenic stimulus.

In (A), (C), (E), and (F), individual CB samples are shown; gray lines connect parameters from the same condition. **p < 0.05 by paired t test. See also Figure S4.

280 days in mouse and human, respectively (Catlin et al., 2011; Wilson et al., 2008), we sought to quantify consequences of this delay to cell cycle entry in homeostatic conditions. Because it is impossible to experimentally examine homeostatic human HSC pool dynamics over long periods, we turned to computational modeling. Our data strongly suggest that control of cell division is achieved through regulation of quiescence exit and cell cycle transit as two discrete steps. We established an agent-based model to investigate (1) the consequences of independent control of the duration of quiescence exit and (2) the effect of the 5.8 hr delay in LT-HSC quiescence exit. In this model, the maintenance of the number of cells in the system is controlled in a closed loop, and dynamic properties of the model—how often cells divide and how quick the response to injury—arise purely from the different durations of the stages of cell division (Figure S6A). All parameters and assumptions of the model are reported in the Supplemental Experimental Procedures. Most parameters, in particular the division times (mean ± SD), were measured experimentally. When not possible (i.e., HSC pool exit rate and noise), we tested the full range of possible values (discussed in the Supplemental Experimental Procedures) and chose those predicting a number of HSC divisions per year that is in the range reported in the literature for human HSCs (Catlin et al., 2011) (Figures S6B–S6G). With this set of physiologically relevant parameters, we investigated the outcome of (1) a control situation in which division is controlled with one kinetic

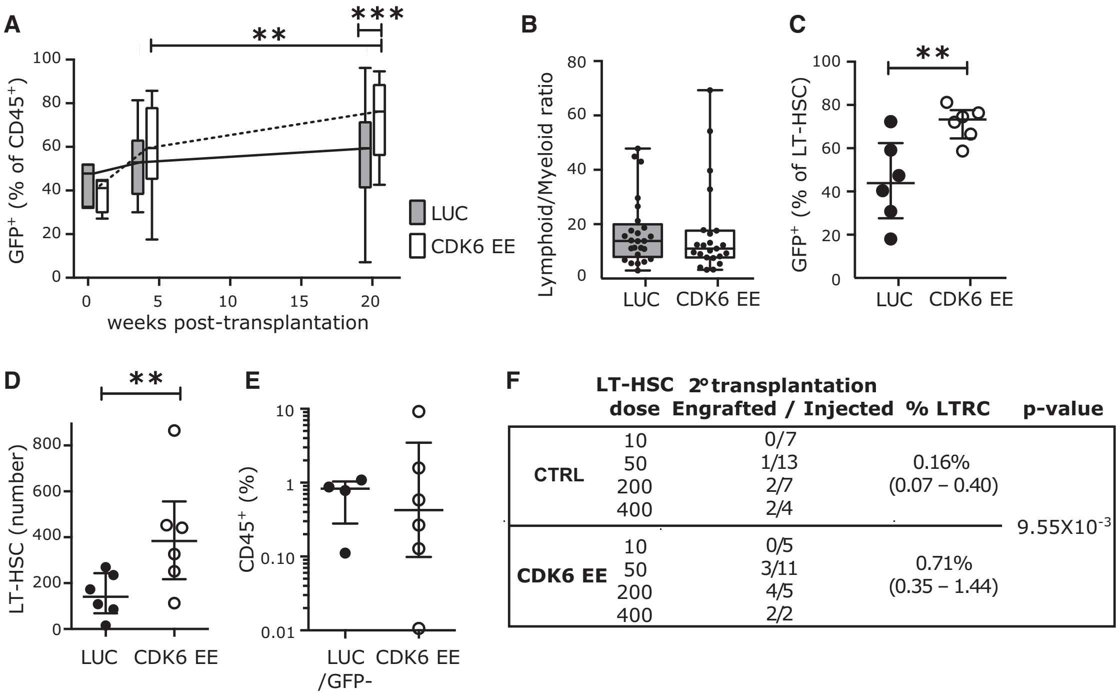

	LT-HSC dose	2° transplantation Engrafted / Injected	% LTRC	p-value
CTRL	10	0/7	0.16% (0.07 – 0.40)	9.55×10^{-3}
	50	1/13		
	200	2/7		
	400	2/4		
CDK6 EE	10	0/5	0.71% (0.35 – 1.44)	
	50	3/11		
	200	4/5		
	400	2/2		

Figure 5. CDK6 EE LT-HSCs Outcompete Wild-Type HSCs without Exhaustion

(A–D) NSG mice were injected with sorted Lin^- $CD34^+$ $CD38^-$ cells transduced with CDK6 EE or control (LUC) lentiviral vectors (GFP^+ cells) and untransduced competitive cells (GFP^-). Bone marrow was harvested at indicated time points post-transplantation and analyzed by flow cytometry. (A) Percentage of GFP^+ cells among engrafted human hematopoietic cells ($CD45^+$). Time 0 corresponds to percentage of GFP^+ cells before injection in four independent CB samples. 4 weeks post-transplantation: n = 13 LUC and 14 CDK6 EE mice; 20 weeks post-transplantation: n = 25 LUC and 23 CDK6 EE mice. (B) Lymphoid to myeloid ratio (percentage of $CD19^+/CD33^+$) among GFP^+ cells at 20 weeks post-transplantation. n = 25 LUC and 23 CDK6 EE mice. In (A) and (B), boxplots represent median, 25th, and 75th percentiles and whiskers represent min and max. Gray boxes, LUC; white boxes, CDK6 EE. (C) Percentage of GFP^+ cells among LT-HSCs at 20 weeks post-transplantation. (D) Absolute number of LT-HSCs at 20 weeks post-transplantation. In (C) and (D), n = 6 mice from two CB samples. Individual mice, median, and interquantile range are shown. In (A)–(D), *$p < 0.1$, **$p < 0.05$, ***$p < 0.01$ by Mann-Whitney test.

(E and F) CDK6 EE LT-HSCs expand over serial transplantation. LUC, CDK6 EE (GFP^+), or untransduced (GFP^-) LT-HSCs were sorted from primary transplanted mice (n = 2 pools of three to five mice) and injected at four different doses into secondary NSG mice. (E) Engraftment levels (percentage of $CD45^+$ cells) 12 weeks after secondary transplantation (>0.01% $CD45^+$ GFP^+ or $CD45^+$ GFP^-) at the two highest doses (200 and 400 cells/mouse). Individual mice, median, and interquantile range are shown. (F) Summary table of number of mice engrafted at each dose tested and estimation of LT-HSC frequencies in each group by the ELDA statistical method. See also Figure S5.

parameter (from reception of signal to the generation of two daughter cells, with cells committed to divide upon sensing the signal), and (2) a situation in which quiescence exit and cell cycle transit are controlled independently, and where commitment to division happens only once the cell has transitioned out of the quiescence exit phase (Figure 6A). We invariably found that the number of LT-HSC divisions is lower when the duration of a cell division starting from G_0 is defined by two independent kinetic parameters (quiescence exit and cell cycle transit) rather than a single parameter describing the average division time (Figures 6B and 6C). Furthermore, the overall number of LT-HSC divisions simulated to occur over 1 year was again decreased with a 5.8 hr delay in LT-HSC $t_{G0\ exit}$ (Figures 6B and 6C). In fact, a delay as short as 2.6 hr was sufficient to significantly spare the number of LT-HSC divisions (Figure 6D). In addition, in response to perturbation such as might be experienced under hematopoietic stress, the rate of recovery in the HSC and progenitor pools was considerably improved by regulation through two kinetic parameters and even further when the delay in $t_{G0\ exit}$ in LT-HSCs was included (Figures 6E, S6H, and S6I). Our model thus demonstrates that the ability to modulate the length of G_0 exit independently of changes in duration of cell cycle transit provides better robustness to homeostatic and stress response hematopoiesis. Importantly, a delay in the duration of G_0 exit in LT-HSCs compared to ST-HSCs leads to further optimization, indicating that regulation of the duration of the G_0 exit phase rather than that of a whole division is key to controlling HSC pool maintenance and hematopoietic system responses.

DISCUSSION

Our study provides key insights into the regulation of cycling within the HSC pool and furthers our understanding of quiescence. We establish that the level of CDK6 functions as a master regulator of the duration of quiescence exit. CDK6 is differentially regulated at the transcriptional and post-transcriptional level in HSC subsets. In particular, the absence of CDK6 protein in LT-HSCs results in a 5–6 hr delay to G_0 exit. The cumulative effect of this delay limits LT-HSC divisions and ultimately preserves

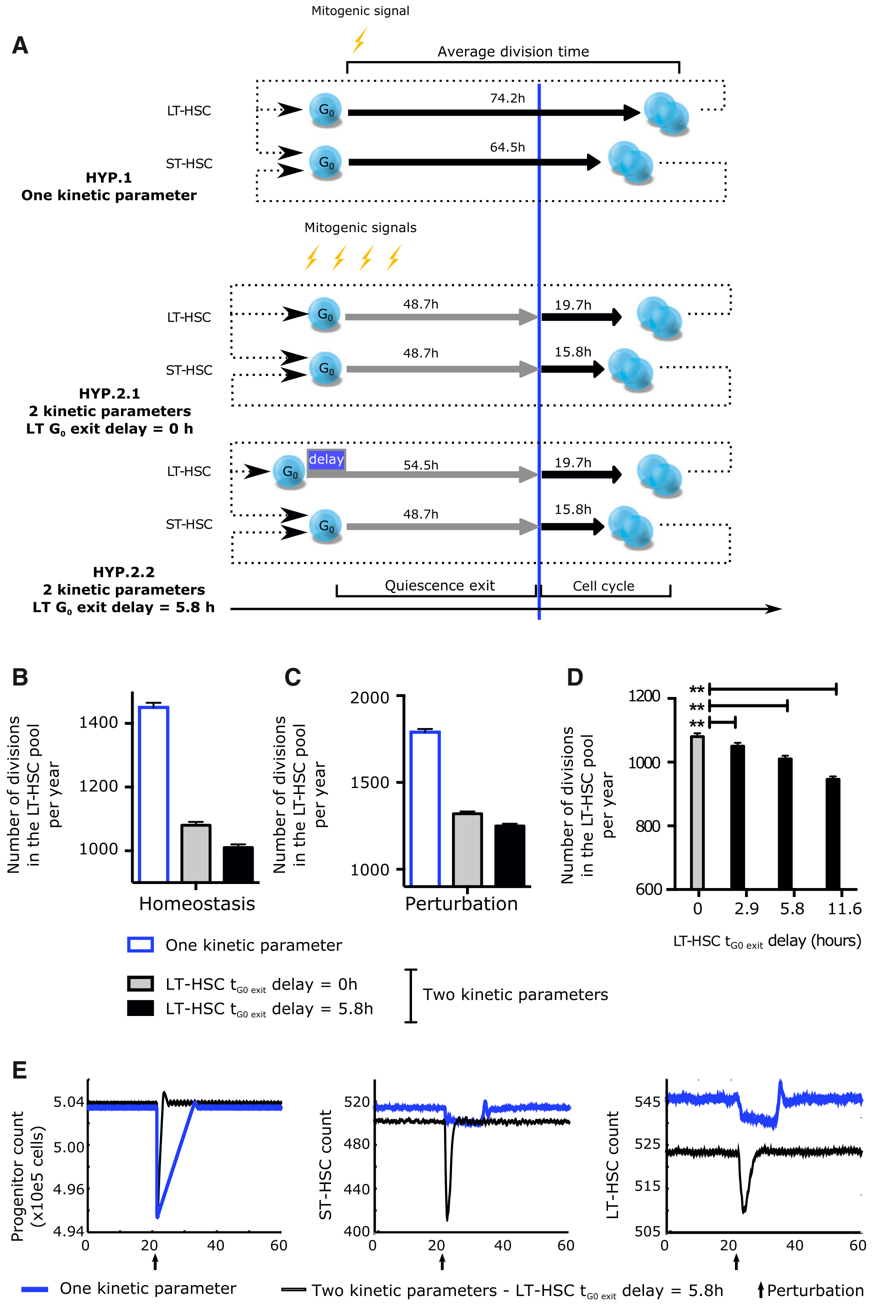

Figure 6. Simulation of the Impact of Delayed G_0 Exit in LT-HSCs on the HSC Pool with an Agent-Based Model

(A) Comparison between the three hypotheses tested by the modeling strategy. Earlier modeling strategies of homeostasis assumed that all HSCs started division upon receiving a mitogenic signal with one characteristic cycling time per HSC subtype (HYP. 1). Rather, we propose that G_0 exit and cell cycle progression are differentially and independently regulated. This results in two characteristic cycling times per HSC subtype without (HYP. 2.1) or with (HYP. 2.2) a delay in G_0 exit between LT- and ST-HSCs. Cycling times indicated are as measured in Figures 2G–2J. (B and C) Simulated number of LT-HSC divisions per year in the HSC pool at homeostasis (B) and after perturbation (C). (D) Effect of changing the LT-HSC G_0 exit delay parameter on the number of LT-HSC divisions. Shown is the number of divisions in the LT-HSC pool per year when the delay of G_0 exit of LT-HSCs (compared to ST-HSCs) is inputted at 0 (no delay), 2.9, 5.8 (experimental value), or 11.6 hr. (E) Perturbation model: 1% of the progenitor compartment was eliminated at the time indicated by an arrow to simulate injury. Number of progenitor cells (left panel), ST-HSCs (middle panel), and LT-HSCs (right panel) are displayed as a function of time. In (B)–(E), data represent the mean ± SD of 256 runs. The simulations were run with a noise parameter of 5% and an HSC pool exit rate of 24 cells per day. How parameters were chosen and results with different parameters are shown in Figure S6 and discussed in the Supplemental Experimental Procedures.

HSC pool integrity in the long term. Because human HSC possess unique mechanisms to prevent propagation when damaged (van Galen et al., 2014; Milyavsky et al., 2010), we speculate that delayed G_0 exit may also be crucial to coordinate repair and LT-HSC fate choices upon their exposure to stress.

In line with the importance of the relative levels of the CyclinD-CDK partners in mediating cell cycle entry and progression (Sherr and Roberts, 2004), our data indicate that the presence of CDK6 in ST-HSC is sufficient to place these cells in the "starting blocks" for division upon mitogenic signaling. Production and activation of CyclinD-CDK complexes is gradual and involves many levels of regulation including gene transcription, protein stability, assembly, and nuclear import (Sherr and Roberts, 2004). Consistent with our results a recent study found that a constitutive knockout of CDK6 does not affect HSCs in homeostasis, but their activation in vivo by mitogenic signals such as 5-FU or IFN is prevented (Scheicher et al., 2014). In line with what is seen in HSCs, memory T cells segregate fully formed CyclinD3/CDK6 complexes in their cytoplasm, which, upon antigen stimulation, allow them to enter cell cycle faster than naive T cells wherein both CDK6 and CyclinD3 are expressed at much lower levels (Veiga-Fernandes and Rocha, 2004). Together, these findings support a model in which any molecular configuration that puts cells closer to an active CDK/CyclinD complex is likely to result in faster cell cycle entry/G_0 exit. Our data further indicate that deeper (LT-HSCs) and shallower (ST-HSCs) states of quiescence are an embedded feature of the hematopoietic hierarchy at homeostasis. Furthermore, the CDK6-primed G_0 state of ST-HSCs does not overlap with G_{alert}, a recently described injury-stimulus-induced adaptive mechanism that positions stem cells to rapidly respond to further stress by activating the mTORC1 pathway (Rodgers et al., 2014). We found that

homeostatic ST-HSCs display similar levels of mitochondrial and mTORC activity to that of LT-HSCs, indicating that they are not in G_{alert}. Rather, the injury-independent pre-existent diversity in quiescent states that we report coexists with, and is upstream of, G_{alert}.

In a high output system like blood, which is sustained by a limited number of active HSCs, a number of theoretical frameworks describe how HSC heterogeneity, notably in division rates, contributes to lifelong maintenance of hematopoiesis. Previous modeling strategies (Glauche et al., 2009; Hoffmann et al., 2008; Roeder and Loeffler, 2002) describe the division properties of HSCs by a single, unique parameter that is usually derived from average division frequencies and thus includes the time spent in G_0 plus the time from the reception of the signal to the end of division. In contrast to these prior studies, we explicitly model the control of the duration of cell division from the time of the mitogenic signal, using a computational model where the signal is automatically generated depending on needs. This framework allows investigation of which division duration control strategy better preserves HSC pool integrity and maximizes system responsiveness. In this context, "quiescence exit" is a phase during which cells receive and accumulate signals prior to committing to division. Our simulations show that when cell division controlled by two independent characteristic times (one before and one after the commitment point), it is far more efficient than if cells are committed to divide within a fixed period after sensing a signal. Control is further optimized when quiescence exit is differentially regulated between LT- and ST-HSCs. Thus, we propose that, even though its molecular boundaries remain to be defined, the duration of quiescence exit is a biologically relevant time interval and the regulation of this duration inherently determines the dynamics of blood formation. Overall our data point to a model of homeostasis where the deeply quiescent state of LT-HSCs with a long duration of exit from quiescence and the CDK6-primed G_0 state of ST-HSCs together provide a means to achieve efficient production of cells from ST-HSCs while limiting the number of divisions that LT-HSCs undergo.

Our data show that when the G_0 exit delay is abolished due to CDK6-enforced expression, the CDK6 EE LT-HSCs divide more in repopulation assays, due to repeated rounds of accelerated G_0 exits. Accelerating exit from quiescence (at least via CDK6 EE) does not alter the balance between self-renewal and differentiation or impair LT-HSC maintenance; rather, LT-HSCs acquire a competitive advantage. This result is in striking contrast with most situations of increased cycling presented in the literature (reviewed in Pietras et al., 2011) that cause impaired HSC function. Interestingly, two of the most notable examples that increase HSC division without damaging their long-term function are p18 knockout (Yuan et al., 2004) and miR-126 knockdown (Lechman et al., 2012). Because p18 is an Ink4 family member known to repress CDK6, and because our recent data suggest that miR-126 also targets CDK6 (E. Lechman and J.E.D., unpublished data), it will be interesting to verify if the delay in G_0 exit of LT-HSCs is also suppressed in these models. Similarly, it needs to be addressed how G_0 exit duration is affected in cases where increased cycling leads to HSC exhaustion. Indeed, why increased cycling is generally associated with impaired LT-HSC maintenance remains hypothetical (Orford and Scadden, 2008; Pietras et al., 2011; Rossi et al., 2012). In view of our own results, we speculate that LT-HSCs may be pushed toward differentiation at the expense of self-renewal if they shorten or bypass phases of the cell cycle other than G_0 exit, undergo several rounds of division without returning to G_0, or show imbalances in key differentiation genes. Importantly, our experimental and computational modeling data establish that fully functional LT-HSCs can acquire competitive advantages in a purely kinetic way. Such a phenomenon may be crucial during aging or in the initial steps of leukemia, where clonal dominance may uniquely arise as a consequence of the accelerated duration of G_0 exit of LT-HSCs. Furthermore, recent work indicates that PD033299, which selectively inhibits CDK6, might be efficacious against multiple myeloma (Huang et al., 2012) and MLL-rearranged AML (Placke et al., 2014), malignancies where the preleukemic cell of origin is thought to be an HSC. Overall, the finding that the duration of G_0 exit is a highly relevant biological parameter that controls stem cell pool dynamics warrants further investigation of whether perturbation of stem cell-specific quiescence exit mechanisms represents an early step of malignancy.

EXPERIMENTAL PROCEDURES

CB Lineage Depletion

All CB samples were obtained with informed consent according to procedures approved by the institutional review boards of the University Health Network, Trillium, and Credit Valley Hospital. Mononuclear cells were obtained by centrifugation on Lymphoprep medium (Stem Cell Technologies) and were depleted of Lin^+ cells (lineage depletion) by negative selection with the StemSep Human Progenitor Cell Enrichment Kit according to the manufacturer's protocol (Stem Cell Technologies). Lin^- CB cells were stored at $-150°C$.

Cell Preparation for Cell Sorting

Lin^- cells were thawed by drop-wise addition of IMDM/DNase (100 μg/ml, Roche) and were resuspended at 1×10^6 cells /ml. Cells were then stained with the following (with all antibodies from BD, unless stated otherwise): FITC—anti-CD45RA (1:50, 555488), PE—anti-CD90 (1:50, 555596), PECy5—anti-CD49f (1:50, 551129), V450—anti-CD7 (1:33.3, 642916), PECy7—anti-CD38 (1:100, 335790), APC—anti-CD10 (1:50, 340923), and APCCy7—anti-CD34 (1:100, custom made by BD). Cells were sorted on FACS Aria III (Becton Dickinson) or MoFlo (Beckman Coulter) sorters, consistently yielding >95% purity. LT-HSCs were sorted based on the following markers: $CD34^+$ $CD38^-$ $CD45RA^-$ $CD90^+$ $CD49f^+$; ST-HSCs, based on $CD34^+$ $CD38^-$ $CD45RA^-$ $CD90^-$ $CD49f^-$; and GMPs, based on $CD34^+$ $CD38^-$ $CD10^-$ $CD7^-$ $CD45RA^+$.

Single-Cell Experiments

Single LT-HSCs or ST-HSCs were sorted into 96-well round-bottom Nunc plates in 100 μl of either high or low cytokines media, using FACS Aria III (Becton Dickinson). Cells were centrifuged 5 min at 400 × *g* and incubated at 37°C for 1 week. Cells were visualized and counted in each well twice a day using an inverted microscope. High cytokine condition medium was by StemPro (Stem Cell Technologies) supplemented with StemPro nutrients (Stem Cell Technologies), L-glutamine (GIBCO), Pen/Strep (GIBCO), human LDL (Stem Cell Technologies, 50 ng/ml), and the following cytokines (all from Miltenyi): SCF (100 ng/ml), Flt3L (20 ng/ml), TPO (100 ng/ml), EPO (3 units/ml), IL-6 (50 ng/ml), IL-3 (10 ng/ml), and GM-CSF (20 ng/ml). Low cytokine condition medium was composed of X-VIVO 10 medium (BioWhittaker) supplemented with 1% BSA (Roche), L-glutamine (GIBCO), Pen/Strep (GIBCO), and the following cytokines (all from Miltenyi): SCF (100 ng/ml), Flt3L (100 ng/ml), TPO (50 ng/ml), and IL7 (IL-7; 10 ng/ml).

Immunofluorescence

5×10^3 sorted LT-HSCs, ST-HSCs, or GMPs sorted by flow cytometry were fixed ovre 10 min at room temperature (RT) in PBS and 2%

paraformaldehyde, washed in PBS, distributed in 150 μl of PBS on polylysine-coated slides, and incubated overnight in a humidified chamber at RT. Cells were then permeabilized over 10 min in 0.2% Triton (SIGMA), washed twice in PBS, and blocked over 20 min using 150 μl of PBS and 10% Goat Serum (Life Technologies). Cells were stained over 1 hr at RT in 150 μl of primary antibody solution in PBS and 10% Goat Serum with appropriate concentrations (CDK6, mouse monoclonal ab54576, Abcam, or CDK6 B-10, Santa Cruz sc7961; mouse IgG, Santa Cruz sc-2025; Cyclin D3 (C-16), Santa Cruz: sc-182; rabbit IgG, Santa Cruz sc-2027). After cells were washed twice in PBS, secondary antibody solution (goat anti-mouse Alexa 488, Life Technologies, A11001) was added over 45 min at RT in the dark in 150 μl PBS (10% Goat Serum) with the appropriate concentration (usually 1:500). Slides were visualized on an Axioimager microscope and fluorescence quantification and cell diameter measurements were performed with ImageJ software.

Cell cycle analysis assay, xenotransplantation, the derivation of cell cycle parameters, modeling, transcriptome studies, bioinformatics, qPCR, lentiviral transduction, and mitochondrial mass measurements are reported in the Supplemental Experimental Procedures.

ACCESSION NUMBERS

The GEO database accession number for the gene expression data reported in this paper is GSE58299.

SUPPLEMENTAL INFORMATION

Supplemental Information for this article includes six figures, two tables, and Supplemental Experimental Procedures and can be found with this article online at http://dx.doi.org/10.1016/j.stem.2015.01.017.

AUTHOR CONTRIBUTIONS

E.L. and J.E.D designed the study; E.L., S.X., C.F. and C.F. analyzed and interpreted the data; E.L., S.X., C.F., R.F, S.Z., and SD performed experiments; C.F.D. performed computational modeling; A.N. and I.P. cloned lentiviral vector plasmids and did RT-PCR; J.C. and C.A. did gene-expression profiling experiments;, J.B.F. and N.I. supervised specific experiments; E.L. wrote the manuscript; E.L., S.X., C.F., R.F, C.F.D, N.I., and J.E.D edited the manuscript; and J.E.D. supervised the study. E.L., C.F., and S.X. contributed equally to this study.

ACKNOWLEDGMENTS

We thank the obstetrics unit of Trillium Health Partners (Missisauga and Credit Valley sites) for cord blood samples; N. Simard, P.A. Penttilä, A. Khandi, L. Jamieson, and S. Zhao at the UHN-SickKids Flow Cytometry Facility for cell sorting; V. Voisin and G. Bader for advice on bioinformatics; and M. Doedens for help with intrafemoral injections. This work was supported by the Swiss National Science Foundation (E.L.), Roche (E.L.), the Fondation Suisse pour les Bourses en Médecine et Biologie (E.L.), the Swedish Research Council (S.Z.); and a Canadian Institutes of Health Research (CIHR) fellowship in partnership with the Aplastic Anemia and Myelodysplasia Association of Canada (S.Z.). Work in J.E.D.'s laboratory is supported by grants from the CIHR, Canadian Cancer Society, Terry Fox Foundation, Genome Canada through the Ontario Genomics Institute, Ontario Institute for Cancer Research with funds from the province of Ontario, a Canada Research Chair, the Princess Margaret Hospital foundation, and the Ontario Ministry of Health and Long Term Care (OMOHLTC). Research in E.L.'s laboratory is currently supported by a recruitment support from the Wellcome Trust and a core support grant from the Wellcome Trust and MRC to the Wellcome Trust – Medical Research Council Cambridge Stem Cell Institute.

Received: November 25, 2014
Revised: January 10, 2015
Accepted: January 28, 2015
Published: February 19, 2015

REFERENCES

Aryee, M.J., Gutiérrez-Pabello, J.A., Kramnik, I., Maiti, T., and Quackenbush, J. (2009). An improved empirical bayes approach to estimating differential gene expression in microarray time-course data: BETR (Bayesian Estimation of Temporal Regulation). BMC Bioinformatics *10*, 409.

Benveniste, P., Frelin, C., Janmohamed, S., Barbara, M., Herrington, R., Hyam, D., and Iscove, N.N. (2010). Intermediate-term hematopoietic stem cells with extended but time-limited reconstitution potential. Cell Stem Cell *6*, 48–58.

Bradford, G.B., Williams, B., Rossi, R., and Bertoncello, I. (1997). Quiescence, cycling, and turnover in the primitive hematopoietic stem cell compartment. Exp. Hematol. *25*, 445–453.

Catlin, S.N., Busque, L., Gale, R.E., Guttorp, P., and Abkowitz, J.L. (2011). The replication rate of human hematopoietic stem cells in vivo. Blood *117*, 4460–4466.

Cheshier, S.H., Morrison, S.J., Liao, X., and Weissman, I.L. (1999). In vivo proliferation and cell cycle kinetics of long-term self-renewing hematopoietic stem cells. Proc. Natl. Acad. Sci. USA *96*, 3120–3125.

Copley, M.R., Beer, P.A., and Eaves, C.J. (2012). Hematopoietic stem cell heterogeneity takes center stage. Cell Stem Cell *10*, 690–697.

Foudi, A., Hochedlinger, K., Van Buren, D., Schindler, J.W., Jaenisch, R., Carey, V., and Hock, H. (2009). Analysis of histone 2B-GFP retention reveals slowly cycling hematopoietic stem cells. Nat. Biotechnol. *27*, 84–90.

Gazit, R., Garrison, B.S., Rao, T.N., Shay, T., Costello, J., Ericson, J., Kim, F., Collins, J.J., Regev, A., Wagers, A.J., and Rossi, D.J.; Immunological Genome Project Consortium (2013). Transcriptome analysis identifies regulators of hematopoietic stem and progenitor cells. Stem Cell Reports *1*, 266–280.

Glauche, I., Moore, K., Thielecke, L., Horn, K., Loeffler, M., and Roeder, I. (2009). Stem cell proliferation and quiescence—two sides of the same coin. PLoS Comput. Biol. *5*, e1000447.

Hoffmann, M., Chang, H.H., Huang, S., Ingber, D.E., Loeffler, M., and Galle, J. (2008). Noise-driven stem cell and progenitor population dynamics. PLoS ONE *3*, e2922.

Huang, X., Di Liberto, M., Jayabalan, D., Liang, J., Ely, S., Bretz, J., Shaffer, A.L., 3rd, Louie, T., Chen, I., Randolph, S., et al. (2012). Prolonged early G(1) arrest by selective CDK4/CDK6 inhibition sensitizes myeloma cells to cytotoxic killing through cell cycle-coupled loss of IRF4. Blood *120*, 1095–1106.

Jankovic, V., Ciarrocchi, A., Boccuni, P., DeBlasio, T., Benezra, R., and Nimer, S.D. (2007). Id1 restrains myeloid commitment, maintaining the self-renewal capacity of hematopoietic stem cells. Proc. Natl. Acad. Sci. USA *104*, 1260–1265.

Kataoka, K., Sato, T., Yoshimi, A., Goyama, S., Tsuruta, T., Kobayashi, H., Shimabe, M., Arai, S., Nakagawa, M., Imai, Y., et al. (2011). Evi1 is essential for hematopoietic stem cell self-renewal, and its expression marks hematopoietic cells with long-term multilineage repopulating activity. J. Exp. Med. *208*, 2403–2416.

Lajtha, L.G. (1963). On the concept of cell cycle. J. Cell. Physiol. *62* (1), L1, 143–145.

Laurenti, E., Varnum-Finney, B., Wilson, A., Ferrero, I., Blanco-Bose, W.E., Ehninger, A., Knoepfler, P.S., Cheng, P.-F., MacDonald, H.R., Eisenman, R.N., et al. (2008). Hematopoietic stem cell function and survival depend on c-Myc and N-Myc activity. Cell Stem Cell *3*, 611–624.

Laurenti, E., Doulatov, S., Zandi, S., Plumb, I., Chen, J., April, C., Fan, J.-B., and Dick, J.E. (2013). The transcriptional architecture of early human hematopoiesis identifies multilevel control of lymphoid commitment. Nat. Immunol. *14*, 756–763.

Lechman, E.R., Gentner, B., van Galen, P., Giustacchini, A., Saini, M., Boccalatte, F.E., Hiramatsu, H., Restuccia, U., Bachi, A., Voisin, V., et al. (2012). Attenuation of miR-126 activity expands HSC in vivo without exhaustion. Cell Stem Cell *11*, 799–811.

Micklem, H.S., and Ogden, D.A. (1976). Ageing of Haematopoietic Stem Cell Populations in the Mouse. In Stem Cells of Renewing Cell Population, A.B. Cairnie, P.K. Lala, and D.G. Osmond, eds. (Academic Press), pp. 331–341.

Milyavsky, M., Gan, O.I., Trottier, M., Komosa, M., Tabach, O., Notta, F., Lechman, E., Hermans, K.G., Eppert, K., Konovalova, Z., et al. (2010). A distinctive DNA damage response in human hematopoietic stem cells reveals an apoptosis-independent role for p53 in self-renewal. Cell Stem Cell *7*, 186–197.

Morrison, S.J., and Weissman, I.L. (1994). The long-term repopulating subset of hematopoietic stem cells is deterministic and isolatable by phenotype. Immunity *1*, 661–673.

Notta, F., Doulatov, S., Laurenti, E., Poeppl, A., Jurisica, I., and Dick, J.E. (2011). Isolation of single human hematopoietic stem cells capable of long-term multilineage engraftment. Science *333*, 218–221.

Oguro, H., Ding, L., and Morrison, S.J. (2013). SLAM family markers resolve functionally distinct subpopulations of hematopoietic stem cells and multipotent progenitors. Cell Stem Cell *13*, 102–116.

Orford, K.W., and Scadden, D.T. (2008). Deconstructing stem cell self-renewal: genetic insights into cell-cycle regulation. Nat. Rev. Genet. *9*, 115–128.

Passegué, E., Wagers, A.J., Giuriato, S., Anderson, W.C., and Weissman, I.L. (2005). Global analysis of proliferation and cell cycle gene expression in the regulation of hematopoietic stem and progenitor cell fates. J. Exp. Med. *202*, 1599–1611.

Pietras, E.M., Warr, M.R., and Passegué, E. (2011). Cell cycle regulation in hematopoietic stem cells. J. Cell Biol. *195*, 709–720.

Pietrzyk, M.E., Priestley, G.V., and Wolf, N.S. (1985). Normal cycling patterns of hematopoietic stem cell subpopulations: an assay using long-term in vivo BrdU infusion. Blood *66*, 1460–1462.

Placke, T., Faber, K., Nonami, A., Putwain, S.L., Salih, H.R., Heidel, F.H., Krämer, A., Root, D.E., Barbie, D.A., Krivtsov, A.V., et al. (2014). Requirement for CDK6 in MLL-rearranged acute myeloid leukemia. Blood *124*, 13–23.

Qiu, J., Papatsenko, D., Niu, X., Schaniel, C., and Moore, K. (2014). Divisional History and Hematopoietic Stem Cell Function during Homeostasis. Stem Cell Rep. *2*, 473–490.

Ren, S., and Rollins, B.J. (2004). Cyclin C/cdk3 promotes Rb-dependent G0 exit. Cell *117*, 239–251.

Rodgers, J.T., King, K.Y., Brett, J.O., Cromie, M.J., Charville, G.W., Maguire, K.K., Brunson, C., Mastey, N., Liu, L., Tsai, C.-R., et al. (2014). mTORC1 controls the adaptive transition of quiescent stem cells from G0 to G(Alert). Nature *510*, 393–396.

Roeder, I., and Loeffler, M. (2002). A novel dynamic model of hematopoietic stem cell organization based on the concept of within-tissue plasticity. Exp. Hematol. *30*, 853–861.

Rossi, L., Lin, K.K., Boles, N.C., Yang, L., King, K.Y., Jeong, M., Mayle, A., and Goodell, M.A. (2012). Less is more: unveiling the functional core of hematopoietic stem cells through knockout mice. Cell Stem Cell *11*, 302–317.

Scheicher, R., Hoelbl-Kovacic, A., Bellutti, F., Tigan, A.-S., Prchal-Murphy, M., Heller, G., Schneckenleithner, C., Salazar-Roa, M., Zöchbauer-Müller, S., Zuber, J., et al. (2014). CDK6 as a key regulator of hematopoietic and leukemic stem cell activation. Blood *125*, 90–101.

Sherr, C.J., and Roberts, J.M. (2004). Living with or without cyclins and cyclin-dependent kinases. Genes Dev. *18*, 2699–2711.

Suda, T., Suda, J., and Ogawa, M. (1983). Proliferative kinetics and differentiation of murine blast cell colonies in culture: evidence for variable G0 periods and constant doubling rates of early pluripotent hemopoietic progenitors. J. Cell. Physiol. *117*, 308–318.

Takizawa, H., Regoes, R.R., Boddupalli, C.S., Bonhoeffer, S., and Manz, M.G. (2011). Dynamic variation in cycling of hematopoietic stem cells in steady state and inflammation. J. Exp. Med. *208*, 273–284.

Uchida, N., Dykstra, B., Lyons, K.J., Leung, F.Y.K., and Eaves, C.J. (2003). Different in vivo repopulating activities of purified hematopoietic stem cells before and after being stimulated to divide in vitro with the same kinetics. Exp. Hematol. *31*, 1338–1347.

van Galen, P., Kreso, A., Mbong, N., Kent, D.G., Fitzmaurice, T., Chambers, J.E., Xie, S., Laurenti, E., Hermans, K., Eppert, K., et al. (2014). The unfolded protein response governs integrity of the haematopoietic stem-cell pool during stress. Nature *510*, 268–272.

Veiga-Fernandes, H., and Rocha, B. (2004). High expression of active CDK6 in the cytoplasm of CD8 memory cells favors rapid division. Nat. Immunol. *5*, 31–37.

Wilson, A., Laurenti, E., Oser, G., van der Wath, R.C., Blanco-Bose, W., Jaworski, M., Offner, S., Dunant, C.F., Eshkind, L., Bockamp, E., et al. (2008). Hematopoietic stem cells reversibly switch from dormancy to self-renewal during homeostasis and repair. Cell *135*, 1118–1129.

Yuan, Y., Shen, H., Franklin, D.S., Scadden, D.T., and Cheng, T. (2004). In vivo self-renewing divisions of haematopoietic stem cells are increased in the absence of the early G1-phase inhibitor, p18INK4C. Nat. Cell Biol. *6*, 436–442.

Cell Stem Cell

Supplemental Information

CDK6 Levels Regulate Quiescence Exit in Human Hematopoietic Stem Cells

Elisa Laurenti, Catherine Frelin, Stephanie Xie, Robin Ferrari, Cyrille F. Dunant, Sasan Zandi, Andrea Neumann, Ian Plumb, Sergei Doulatov Jin Chen, Craig April, Jian-Bing Fan, Norman Iscove, and John E. Dick

Supplemental Information

Figure S1

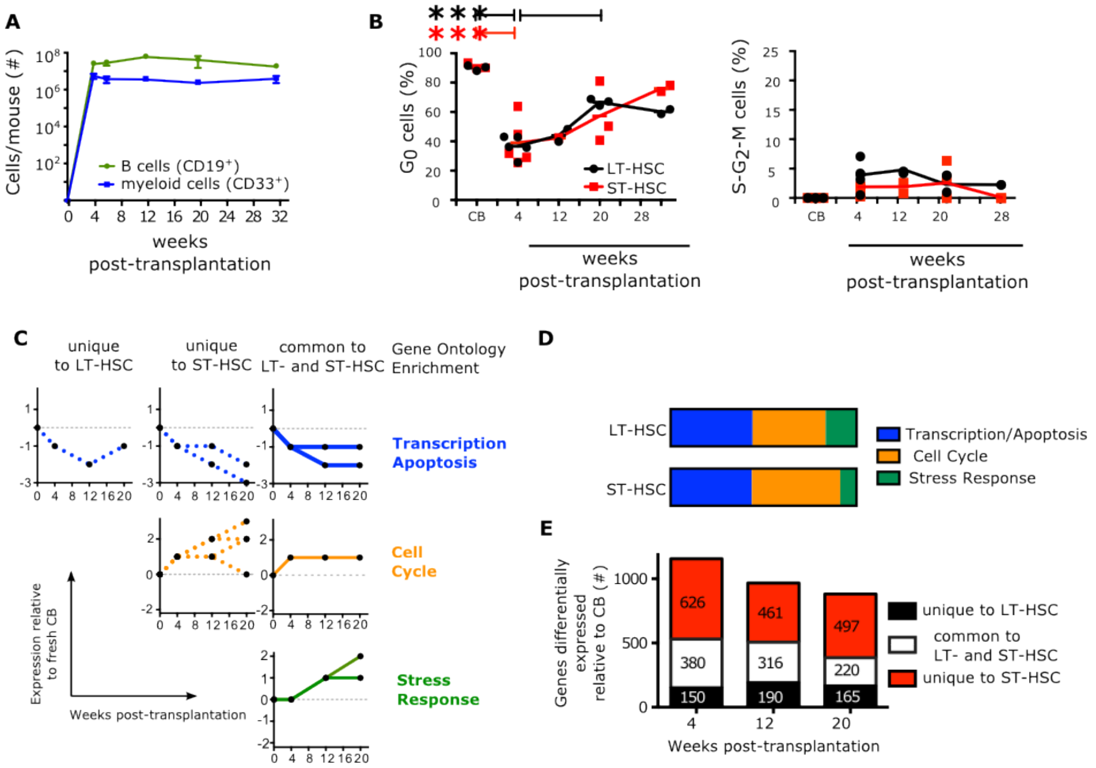

Figure S1, related to Fig.1: Human LT- and ST-HSC functional and transcriptome kinetics upon xenotransplantation.

A, Kinetics of reconstitution of the lymphoid (CD19$^{+)}$ and myeloid lineage (CD33^{+}) in the bone marrow of xenotransplanted mice. Number of cells per mouse of the indicated populations at the indicated time points post transplantation of 70000 Lin^{-} CB (saturating number of LT-HSCs). Median and interquantile range shown. **B,** Quantification of LT-, ST-HSC and progenitor cells in G_0 (Ki67^{-} Hoechst^{-} assayed by flow cytometry as in CB or at the indicated time points post-transplantation. Each point represents individual pools of CB or individual pools of 3-5 mice, and the horizontal bars represent the mean in 2 to 4 independent experiments; black: LT-HSCs, red: ST-HSCs. ***: p<0.01 by ANOVA and Tukey test. **C,** Significant gene

expression profiles observed upon transplantation into NSG mice identified by the STEM algorithm. Lines connecting points represent changes in gene expression relative to CB for each profile. Branching indicates multiple possible behaviors for that group of genes. Profiles with significant correlation were grouped in transcriptional programs (rows), and selected Gene Ontology terms associated with each program are shown in the right panel. **D,** Proportion of genes (%) changed across the time course belonging to the transcriptional programs indicated. **E,** Number of differentially expressed genes in LT- or ST-HSC at the indicated time points after transplantation relative to the same population in CB as determined by analysis with the limma algorithm (FDR<0.05).

Figure S2

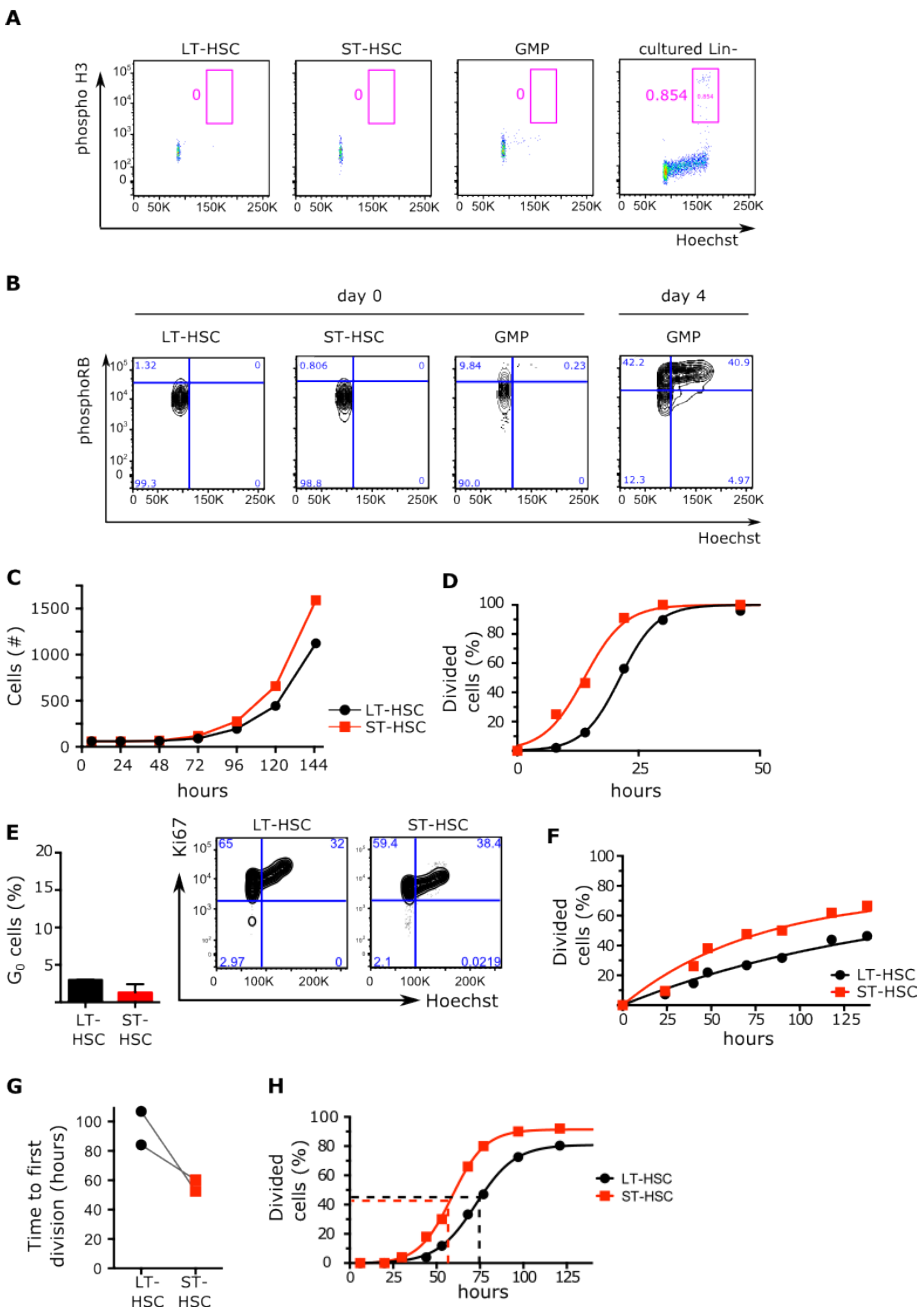

Figure S2, related to Fig.2: characterization of LT- and ST-HSC quiescent states and duration of division upon mitogenic stimulation.

A, absence of phosphoH3 in LT- and ST-HSCs isolated from CB. Examples of representative flow cytometry plots. Pink box represents the gate for phosphoH3 positive cells. n=2 CB. **B,** phosphoRB is absent in LT- and ST-HSCs isolated from

CB. Representative flow cytometry plots of phosphoRB staining (S807/S811 phosphorylation). Cells in the indicated populations either freshly isolated from CB (top panel: day 0) or cultured for 4 days (positive control for the staining, right panel). **C,** ST-HSCs proliferate faster than LT-HSCs in vitro. Growth curve of LT- (black) and ST-HSCs (red), one representative CB is shown, number of initial cells: LT-HSCs: 61, ST-HSC:58. **D,** Cumulative second division kinetics of LT-HSCs (black) and ST-HSCs (red) from a representative CB example. Curve is best sigmoid fit (least squares). R^2 >0.99. **E,** Percentage of cells in the G_0 phase of the cell cycle after 3 days of culture, as measured by flow cytometry ($Ki67^-$ 2n DNA content), representative plot in right panel, quantification from n=2 independent CBs in left panel. **F,G:** In vitro proliferation assay of single LT-HSCs and ST-HSCs in low cytokine and nutrient conditions. n>59 cells from 2 independent CBs. **F,** Cumulative first division kinetics of LT-HSCs (black) and ST-HSCs (red) from a representative CB example. Curve is fit from one-phase association. R^2 >0.96. **G,** Time to first division in hours (half-time). **H,** Cumulative first division kinetics (excluding dead cells) of LT-HSCs (black) and ST-HSCs (red) from a representative adult bone marrow example. Curve is best sigmoid fit (least squares). R^2 >0.99. Arrowheads represent time to first division as estimated from sigmoid fit ($t_{FirstDiv}$ = logEC50).

Figure S3

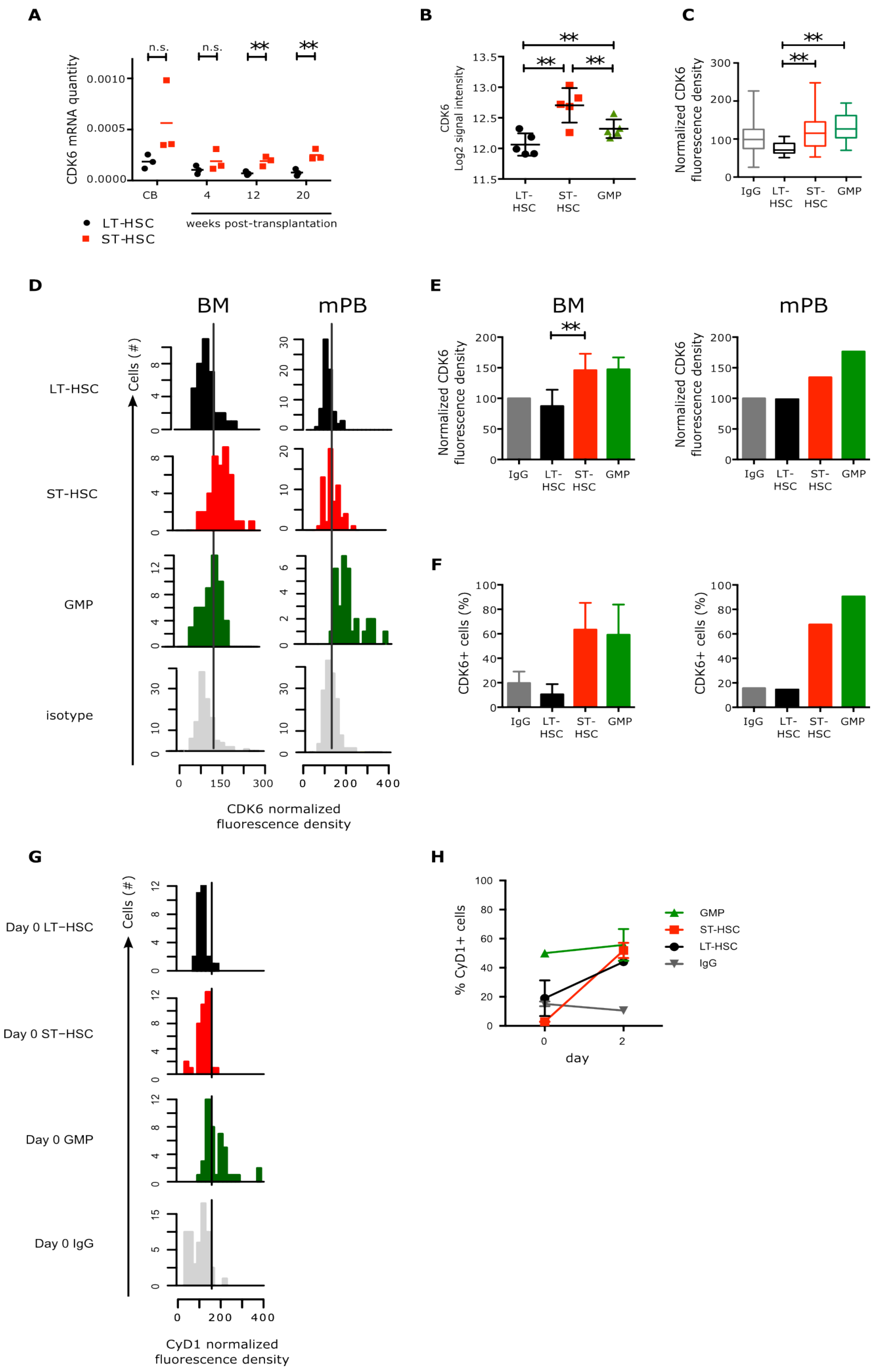

A
CDK6 mRNA quantity
0.0010
0.0005
0.0000
n.s.
n.s.
**
**
CB
4
12
20
weeks post-transplantation
LT-HSC
ST-HSC
B
CDK6
Log2 signal intensity
13.5
13.0
12.5
12.0
11.5
LT-HSC
ST-HSC
GMP
C
Normalized CDK6 fluorescence density
300
200
100
0
IgG
LT-HSC
ST-HSC
GMP
D
BM
mPB
LT-HSC
ST-HSC
GMP
isotype
Cells (#)
CDK6 normalized fluorescence density
E
BM
mPB
Normalized CDK6 fluorescence density
200
150
100
50
0
IgG
LT-HSC
ST-HSC
GMP
F
CDK6+ cells (%)
100
80
60
40
20
0
G
Day 0 LT-HSC
Day 0 ST-HSC
Day 0 GMP
Day 0 IgG
Cells (#)
CyD1 normalized fluorescence density
H
% CyD1+ cells
day
GMP
ST-HSC
LT-HSC
IgG

Figure S3, related to Fig.3: CDK6 is transcriptionally and post-transcriptionally upregulated in ST-HSCs across ontogeny.

A, *CDK6* mRNA is expressed at significantly higher levels in ST-HSCs than in LT-HSCs purified from CB. *CDK6* mRNA quantity as measured by qRT-PCR at the indicated time points. mRNA quantity was normalized to the geometric mean of 2 housekeeping genes (*GAPDH* and *ACTB*). All multiple comparisons have been tested. **B,** Log2 signal intensity for the *CDK6* microarray probe in the dataset attached to this manuscript. Mean ± S.E.M. shown. **A,** n=3; **B,** n=5. **C,** CDK6 protein levels in CB assessed with a different antibody from that used in Fig. 3. Boxplots represent median, 25th and 75th percentiles, whiskers represent min and max. n=33-129 cells from 2 independent CB samples. **D,** CDK6 protein expression in LT- and ST-HSCs isolated from adult hematopoietic tissues. Immunofluorescence for CDK6 protein in LT- and ST-HSCs sorted from adult bone marrow (BM, left panel) or mobilized peripheral blood (mPB, right panel). Shown are histograms of CDK6 fluorescence density normalized to the fluorescence density of the IgG control in the same population. Positivity threshold (dotted line) was set over the median ± 1s.d. of the IgG control distribution. n= 75-194 cells analyzed for BM and n=32-152 cells for mPB. **E,** normalized median CDK6 fluorescence density. Mean ± SEM shown. Left panel: BM; Right panel: mPB. **F,** Percentage of $CDK6^{+}$ cells. Mean ± SEM shown; n=2 BMs and n=1 mPB. **: p<0.05 by paired t-test. **G-H**: Time course analysis of CyclinD1 upon stimulation with mitogenic signals Immunofluorescence for CyclinD1 protein in LT-, ST-HSCs and GMPs from freshly isolated CB (**G**) and 2 days after mitogenic stimulation (**H**). **G:** shown are histograms of CyclinD1 fluorescence density normalized to the fluorescence density of the IgG control in the same population. Positivity threshold (black line) was set over the median + 1s.d. of the

IgG control distribution. **H:** Percentage of CDK6 (top panel) or CyclinD3 (bottom panel) positive cells in each of the indicated populations at the indicated time points after isolation from CB. n=61-191 cells analyzed for day 0 and n=31-156 cells for day 2. Mean ± S.E.M. shown.

Figure S4

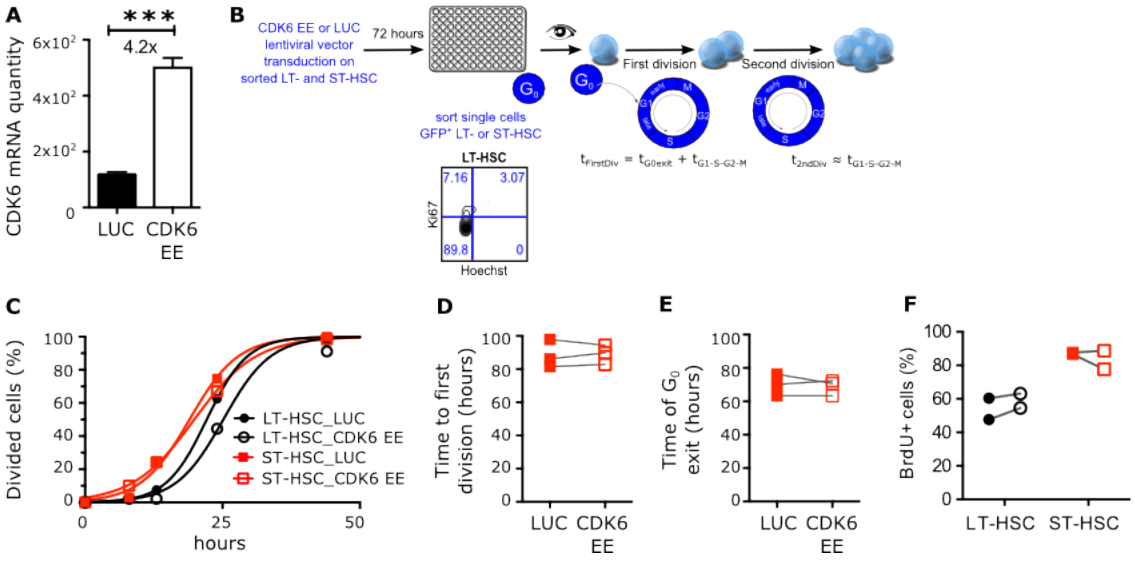

Figure S4, related to Fig.4: CDK6 EE does not affect ST-HSCs.

A, mRNA levels of *CDK6* in Lin⁻ CB cells 3 days after transduction with LUC or CDK6 EE virus. Values are normalized to *GAPDH* and *ACTB*. Mean ± S.E.M. shown. n=3 CB. **B-E:** single cell tracking of LUC or CDK6 EE LT- and ST-HSCs. **B,** Schematic representation of the experiment. Importantly after transduction, cells are cultured in low cytokine and nutrient conditions and do not exit G_0 by the time of the second sort. One representative flow cytometry plot shown (lower panel). Sorted single GFP^+ cells are cultured in high cytokine and nutrient conditions and divisions are monitored. Time 0 represent the time of exposure to mitogenic stimulus. **C,** Cumulative second division kinetics of LT-HSCs (black) and ST-HSCs (red) transduced with LUC (solid symbols) or CDK6EE (empty symbols) from a representative CB example. Curve is best sigmoid fit (least squares). R^2 >0.99. **D,** Time to first division of LUC or CDK6 EE ST-HSC. **E,** Time of G_0 exit LUC or CDK6 EE ST-HSC. ***: p<0.01 and **: p<0.05 and n.s.: non significant by paired t-test. **C-E:** LT-HSCs: black; ST-HSCs: red; filled symbols: LUC; empty symbols:

CDK6 EE. **F.** Relative measure of cell cycle progression through a short pulse of BrdU incorporation**.** LT- and ST-HSCs were transduced with either control (LUC, full symbols) or CDK6 EE (empty symbols), cultured for 4 days then exposed to 100uM BrdU for 8 hours. % BrdU+ cells was then assessed by flow cytometry. n=2 CB

Figure S5

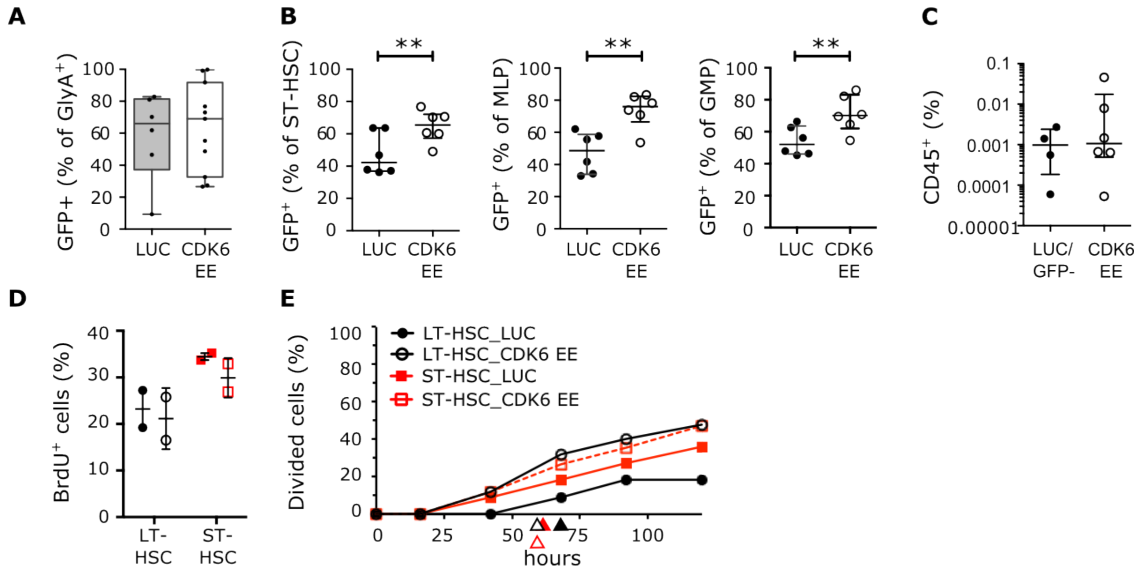

Figure S5, related to Fig.5: CDK6 EE in vivo expands all progenitor populations but does not decrease HSC self-renewal

Competitive xenotransplant experiments were carried out as detailed in Fig. 5. **A,** % GFP^+ among engrafted human erythroid cells ($GlyA^+$). Mice that had less than 0.3% GlyA+ cells were excluded. Boxplots represent median, 25th and 75th percentiles, whiskers represent min and max. n=6 mice for LUC, n=11 mice for CDK6 EE. **B,** Competitive advantage of CDK6 EE LT-HSC persists in downstream progenitors. % GFP^+ among ST-HSC, MLP and GMP at 20 weeks post-transplantation. n=6 mice from 2 CB. Individual mice, median and interquantile range shown. **: $p<0.05$ by Mann-Whitney test. MLP: Multilymphoid Progenitors, GMP: Granulocyte-Monocyte progenitors. **C,** The repopulation potential of CDK6 EE LT-HSCs does not decrease upon secondary transplant. Shown is the % human $CD45^+$ cells relative to the number of CDK6EE or control (LUC) LT-HSCs injected into secondary xenografts. The analysis was performed 12 weeks after secondary transplantation. Shown are

individual mice. n=4 for LUC and n=6 for CDK6 EE. **D,E**: CDK6 EE division kinetics in vivo. **D,** CDK6 EE does not increase BrdU incorporation in vivo. Mice transplanted with CDK6 EE or control (LUC) transduced cells were administered BrdU for 2 days at 8 weeks post-transplantation. The percentage of BrdU positive cells was assayed by flow cytometry in CDK6 EE or LUC LT- and ST-HSCs. Shown are individual measures (circles, LT-HSCs; squares, ST-HSCs) and median (horizontal bar). n=2 pools of 3-5 mice. **E,** CDK6 EE LT-HSCs harvested from xenografts perform their first division as fast as ST-HSCs. Single LT- and ST-HSCs with LUC or CDK6 EE were isolated from xenografted mice at 20 weeks post-transplantation and cultured in cytokine conditions promoting HSC self-renewal (SCF, TPO, FLT3). Divisions were monitored over time. Arrowheads indicate time to first division. n=25-66 cells from pools of 2-3 mice.

Figure S6

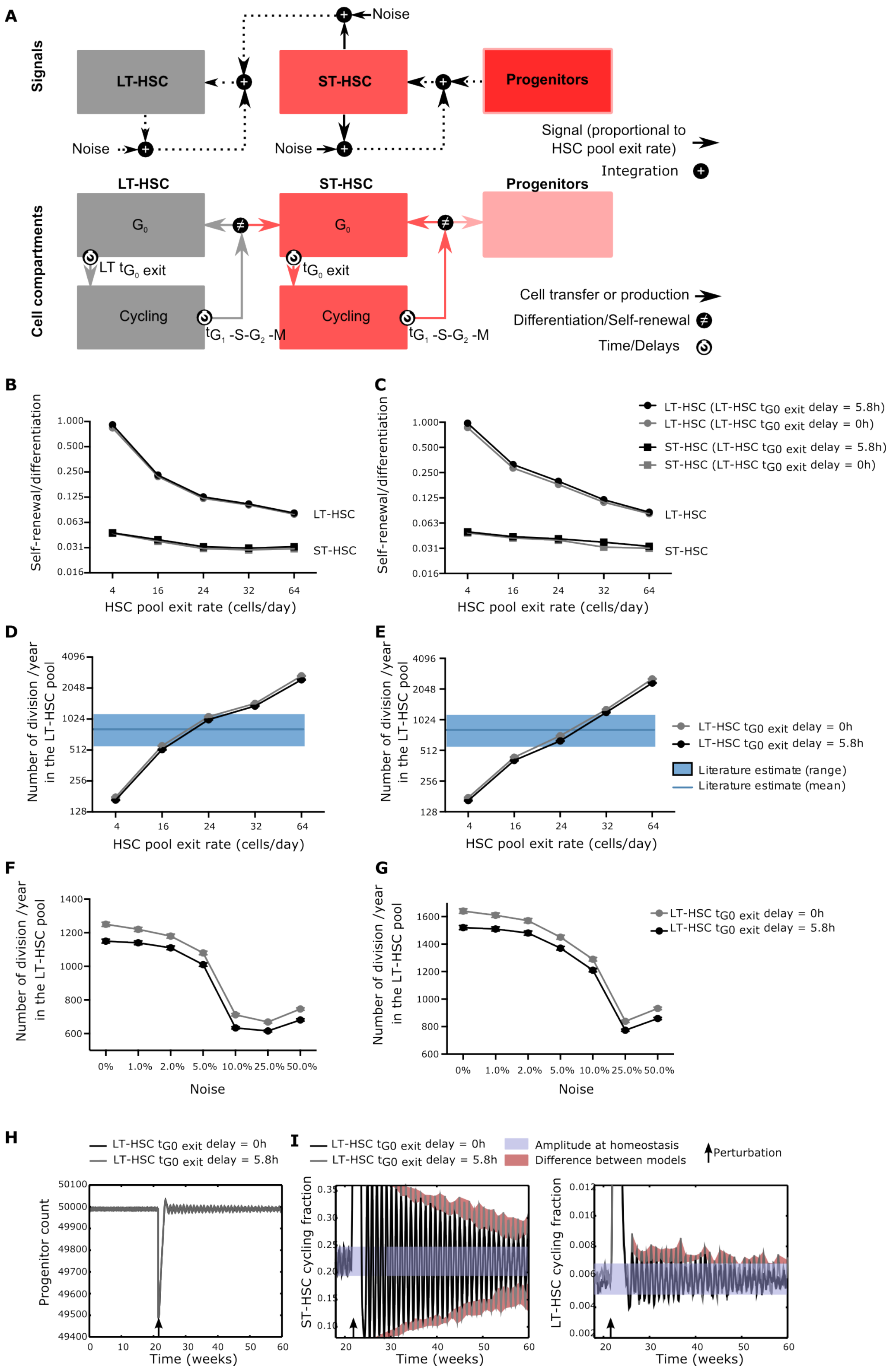

A
Signals
LT-HSC
ST-HSC
Progenitors
Noise
Signal (proportional to HSC pool exit rate)
Integration
Cell compartments
LT tG0 exit
tG0 exit
Cycling
tG1 -S-G2 -M
Cell transfer or production
Differentiation/Self-renewal
Time/Delays
B
C
Self-renewal/differentiation
HSC pool exit rate (cells/day)
LT-HSC (LT-HSC tG0 exit delay = 5.8h)
LT-HSC (LT-HSC tG0 exit delay = 0h)
ST-HSC (LT-HSC tG0 exit delay = 5.8h)
ST-HSC (LT-HSC tG0 exit delay = 0h)
D
E
Number of division /year in the LT-HSC pool
LT-HSC tG0 exit delay = 0h
LT-HSC tG0 exit delay = 5.8h
Literature estimate (range)
Literature estimate (mean)
F
G
Noise
H
I
Progenitor count
Time (weeks)
ST-HSC cycling fraction
LT-HSC cycling fraction
Amplitude at homeostasis
Difference between models
Perturbation

Figure S6, related to Fig.6 and Supplemental Experimental procedures: Agent-based model of the impact of LT-HSC G_0 exit delay on hematopoiesis.

A, Schematic representation of the agent-based model. Top panel: the signals that push cells into division move upstream as indicated by the dashed arrow. Bottom panel: hierarchical organisation of populations and how cells move from one compartment to the other (solid arrows) upon division. Moving from any state (G_0 to Cycling, Cycling to G_0) is characterised by a time unique to the state and cell type as measured experimentally and indicated in Fig.6A. **B-E**: Effect of modifying the HSC pool exit rate. **B-C,** Self-renewal/differentiation ratio of LT- and ST-HSCs as a function of the HSC exit rate (cells/day) when the simulations were run either with 5% (**B**) or 10% (**C**) noise. **D-E:** number of divisions in the LT-HSC pool (500 cells) per year as a function of the HSC exit rate (cells/day) when the simulations were run either with 5% (**D**) or 10% (**E**) noise. The blue band represents the range of LT-HSC division per year based on experimental estimations (Catlin et al., 2011). **F-G,** Effect of modifying noise levels. Number of divisions in the LT-HSC pool (500 cells) per year as a function of the noise (%) when the simulations were run either with an exit rate of 24 (**F**) or 32 (**G**) cells/day. **H-I** Perturbation model: 1% of the progenitors compartment was eliminated at the time indicated by an arrow to simulate injury. Shown are the simulations run with the noise parameter at 5%. **H,** Number of progenitor cells as a function of time. **I,** Fraction of HSC cycling as a function of time. Left panels: ST-HSC; Right panels: LT-HSCs. Homeostatic amplitude is highlighted by a light purple box, while the difference between the model with LT-HSC t_{Goexit} delay = 0 hours and that with LT-HSC t_{Goexit} delay = 5.8 hours is shown in the red shaded area. The data relative to this figure panels are also discussed in Supplemental Experimental Procedures.

Table S1, related to Fig.1: Limiting dilution analysis of cell populations isolated from xenografts at the time indicated.

Time of isolation after primary transplantation	Cell surface phenotype	Dose (cells)	Secondary transplantation Engrafted /Total mice	% LTRC	p-value
4 weeks	CD49f$^+$ CD90$^+$	50	2/11	0.48%	$1.91x10^{-3}$
		300	4/5	(0.20 - 1.10)	
	CD49f$^-$ CD90$^-$	50	0/8	0 - 0.19	
		300	0/4		
12 weeks	CD49f$^+$ CD90$^+$	150	2/6	0.40%	$2.64x10^{-3}$
		750	6/6	(0.17 - 0.92)	
	CD49f$^-$ CD90$^-$	150	0/6	0.04%	
		750	2/6	(0.01 - 0.17)	
20 weeks	CD49f$^+$ CD90$^+$	10	0/2	1.28%	$1.34x10^{-6}$
		50	4/6	(0.61 - 2.70)	
		200	6/6		
		400	4/4		
	CD49f$^-$ CD90$^-$	50	0/3	0 - 0.15	
		200	0/5		
		400	0/2		

Primary xenograft mice were transplanted with 70000 Lin$^-$ cells to establish robust human engraftment. At the indicated time points after primary transplantation (4, 12 or 20 weeks, first column), Lin$^-$ CD34$^+$ CD38$^-$ CD45RA$^-$ CD49f$^+$ CD90$^+$ and Lin$^-$ CD34$^+$ CD38$^-$ CD45RA$^-$ CD49f$^-$ CD90$^-$ were purified from pools of 5-9 primary mice, and were transplanted at the indicated doses in secondary mice. All secondary xenografts were sacrificed 12 weeks after secondary transplantation to verify human engraftment. Engrafted mice: >0.05% human CD45$^+$ in injected femur and non-injected bones. LTRC: Long term repopulating cells. These data collectively show that the same cell surface marker phenotypes used to purify LT- and ST-HSCs out of cord blood can be used to purify LT- and ST-HSCs enriched populations after transplantation into NSG mice.

Table S2, related to Fig.1: list of 241 genes differentially expressed between LT- and ST-HSCs as determined by BETR algorithm.

Laurenti_TableS2.xlsx

Supplemental Experimental Procedures

Cell cycle analysis assays

For Ki67-Hoechst and pRB assays cells were fixed after thawing or flow-sorting with Cytofix/Cytoperm buffer (BD) for 15 min on ice. Cells were stained with FITC-anti-Ki67 (1:50, BD, 556027) or anti-pRB (S807/811, 1:30, Cell Signalling, #8516) overnight in Permwash buffer (BD). For pRB staining, cells were then washed and stained with anti-rabbit IgG Alexa488 (1:500, Life Technologies, A11008) for 30 min, then with Hoechst 33342 (1:10,000; Life Technologies) for 5 min at RT. Samples were analyzed on a LSRII cytometer (BD). For assessment of HSC proliferation in vivo using BrdU incorporation assays, mice were administered drinking water complemented with 4% sucrose and 1 mg/mL BrdU for a 2 to 10 days. Mice were then sacrificed at the indicated timepoints; bone marrow and human CD34+ cells were enriched as described above. Cells were stained with FITC–anti-CD45RA (1:50), PE–anti-CD90 (1:50), PECy5–anti-CD49f (1:50), PECy7–anti-CD38 (1:100), APCCy7–anti-CD34 (1:100), then the BrdU staining was performed with the APC BrdU Flow Kit (BD Pharmingen) with APC-anti-BrdU according to the manufacturer's protocol.

Xenotransplantation

Animal experiments were done in accordance to institutional guidelines approved by University Health Network Animal care committee. Treatments were randomized. NSG mice (NOD.Cg-*Prkdc*scid*Il2rg*tm1Wjl/SzJ; Jackson Laboratory) were sublethally irradiated (250 rads) 24 hours before intrafemoral injection. For xenografts with saturating number of HSC, 6-7x10^4 Lin- CB cells were transplanted per mouse (males or females, 12-16 weeks old), directly after thawing. Competitive

transplantation was performed by injecting each mouse with the equivalent of $1x10^4$ $CD34^+$ $CD38^-$ cells after 4 days of culture in low cytokine conditions medium. In limiting dilution secondary transplantation assays, the indicated sorted populations were injected at defined doses in female mice (12-16 weeks old). LT-HSC frequency was estimated using the ELDA software (http://bioinf.wehi.edu.au/software/elda/; (Hu and Smyth, 2009). For analysis, mice were euthanized at the indicated time points after transplantation. The injected femur and other bones were flushed separately in Iscove's modified Dulbecco's medium (IMDM) and cells were stained with: (dilution of 1:100 and all from BD and unless specified otherwise): phycoerythrin–anti-CD19 (349209), PE–anti-GlyA (Beckman Coulter, A07792), PECy5–anti-CD45 (Beckman Coulter, A07785), PECy7–anti-CD14 (1:200; Beckman Coulter, A22331), APC–anti-CD33 (551378) and V450–anti-CD15 (642917). For LDA experiments 2 CD45 antibodies were used: PECy5–anti-CD45 (Beckman Coulter, A07785) and APC-Cy7-anti-CD45 (557833). For purification of human HSC populations from xenotransplanted mice, bone marrow from individual mice or from pools of 2-10 mice was first enriched for CD34+ cells either by negative selection with the StemSep Human Progenitor Cell Enrichment Kit (Stem Cell Technologies) or by positive selection with the CD34 Microbead kit (Miltenyi) and double column purification by autoMACS technology (Miltenyi). CD34+ enriched cells were then either directly analyzed by flow cytometry or flow-sorted.

Derivation of cell cycle parameters

Single cell experiments data were analyzed with a custom-made R script (version 2.15.2). Briefly the script first excludes all wells with either no cell or more than one cell reported at the beginning of the experiment. The number of cells that have

divided at least once at each time point is then counted. Cumulative first division counts are then plotted over time. Cells that died without dividing are excluded. Similar curves were derived for the second division. The curves obtained by this method were fitted with GraphPad Prism Software (version 6.0b). For high cytokine medium experiments, curves were fitted with a sigmoid model with variable slope. For low cytokine medium conditions curves were fitted with a one-phase association model. Best fits were evaluated with R^2 corrected value. EC50 was calculated as the time at which 50% of cells have divided.

Lentiviral vector constructs and transduction.

Overexpression studies used a bidirectional MA1 vector (Amendola et al., 2005) in which transgene expression is driven by the SFFV promoter (van Galen et al., 2014b). CDK6 cDNA obtained from the Mammalian Gene Collection through PlasmID repository. Viral particles were produced as described (Mazurier et al., 2004) and were titrated on 293T human embryonic kidney cells. For transduction, $5x10^4$ $CD34^+$ $CD38^-$ cells, $5x10^3$ LT- or ST-HSCs purified by flow cytometry were incubated for 3–5 h in low cytokine condition medium. Cells were then incubated for 16-24 hours in the same medium supplemented with virus at a multiplicity of infection of 30–60 transforming units per mL.

Quantitative RT-PCR

RNA was extracted from 1×10^3 to 5×10^3 cells in TRIzol (Life Technologies) supplemented with 25 mg linear polyacrilamyde (LPA; Life Technologies) according to the manufacturer's protocol. Then, cDNA was reverse-transcribed with SuperScript VILO cDNA synthesis kit (Life Technologies) and was purified with QIAquick PCR

purification Kit (QIAGEN). Real-time PCR was done with SYBR Green PCR Master Mix (Applied Biosystems) and 200 nM primers (Qiagen) on an Applied Biosystems 7900HT instrument. *CDK6* and *GAPDH* primers used were Quantitect Primer Assays (Qiagen, *CDK6*: cat #QT00019985, *GAPDH*: cat #QT00079247); *ACTB* primers: forward, CCTGGCACCCAGCACAAT; reverse, GGGCCGGACTCGTCATAC. SDS software (Applied Biosystems) was used for absolute gene-expression quantification by the standard curve method. Two housekeeping genes (*ACTB* and *GAPDH*) were analyzed for comparison; data presented are relative to the geometric mean of expression of those two genes.

Mitochondrial mass measurements

Cord blood mononuclear cells were stained with CD34-APC-Cy7, CD38-PE-Cy7, CD90-PE, CD45RA-FITC, CD49f-PE, washed and then stained with Mitotracker Green FM (Life Technologies, M7514) for 30 minutes at 37°C at 1:40000 for analysis by flow cytometry.

Microarray profiling

RNA was extracted with TRizol (Life Technologies)- cDNA synthesis and preamplification were done as described (Fan et al., 2012). Samples were randomized on the microarray chips to minimize batch effects. Whole-genome gene-expression analysis was performed using the human Whole-Genome DASL HT-12 Assay (version 4.0) R2 (Illumina) (April et al., 2009).

Bioinformatics analysis.

Software of the R project for statistical computing (version 2.15.2) and Bioconductor (version 2.10) were used for all bioinformatics analyses. Quality control was performed using the *P*-detection values of <0.05, hierarchical clustering and PCA based on the Pearson correlation coefficient of all samples. Data were quantile-normalized ('normalizeQuantiles' command of limma software (Linear Models for Microarray Data; version 3.6.9), then were log_2-transformed. All subsequent analyses used this data set. For the derivation of transcriptional programs, the STEM algorithm(Ernst et al., 2005) was used with the following parameters: a maximum of 50 model profiles, a maximum unit change between time points of 1 and a minimum correlation for clustering similar profiles >0.5. For gene-ontology enrichment with this software, *P* values were corrected with 500 randomizations and were considered significant with an FDR of <0.05. Genes differentially expressed between CB and any individual time point post-transplantation were identified with the limma package (version 3.6.9), which calculates the moderated *t*-test statistic for a particular contrast. All *t*-test scores were controlled for multiple-hypothesis testing with the Benjamini-Hochberg method, and genes with an adjusted *P* value of <0.05 were considered differentially expressed. To derive a core signature of gene expression differences between LT- and ST-HSCs in the time-course experiment, the BETR algorithm(Aryee et al., 2009) was used, with α set as 0.05. All genes with adjusted p.value < 0.05 were considered differentially expressed between LT- and ST-HSCs.

Derivation of a model describing the impact of G_0 exit delay on HSC pool dynamics.

Modeling was performed with a dynamic agent based model in a C++ custom code, compiled on g++ -std=c++11 -O3, g++ (SUSE Linux) 4.8.1 20130909 [gcc-4_8-branch revision 202388]. As the dynamics of the model essentially stem from delays, the system is formally of infinite order. It cannot therefore be expressed in differential form. The model consists of two modules: one that concerns the duration of the divisions (Fig.S6A – bottom panel), and one that concerns how the signals to divide are generated (Fig.S6A – top panel). Each cell in the HSC compartment considered is modeled individually: it has its own G_0 exit time and cell cycle transit time. The progenitor compartment is simplified: only the total number of cells in that compartment is tracked. The rules governing the life of each cell are: 1) Each cell has an individual timer. 2) When a new cell arises, it is given a G_0 exit time and cycle time randomly following a Gaussian distribution. The parameters of that Gaussian are given by the mean ± SD measured in the experiments (Fig. 2G-J; Fig.6A). 3) If a cell receives a signal for division for a duration equal or superior to its G_0 exit time, it divides. The signal also contains the information of which cell type is missing. The missing cell type information is averaged over the G_0 exit time. 4) The division gives rise to a new cell, the old one is kept. The nature of the new cell is determined randomly, according to the proportion received in the signal. For LT-HSCs, self-renewal gives rise to a LT-HSC, and asymmetric division gives rise to a new ST-HSCs. For ST-HSCs, self-renewal gives rise to a ST-HSC, and asymmetric division gives rise to a new progenitor. Symmetric division yielding to two progenitors does not occur in this model. 5) Upon division, the cell may die with a 5% probability (corresponding to the average death rate experimentally measured in the HSC compartment; data not shown). In this model, 3 types of signals are generated: 1) The number of progenitors is computed and the difference to the homeostatic number is

calculated. A noise is applied. 2) The number of ST-HSCs is computed and the difference to the homeostatic number is calculated. A noise is applied. 3) the number of LT-HSCs is computed and the difference to the homeostatic number is calculated. A noise is applied. At each time step of the simulation (2 minutes): 1) Cells increment their individual clocks. 2) If necessary, cells divide, self-renew, differentiate or die. 3) The progenitor pool loses a number of cells equal to the HSC exit rate. 4) Signals 1 and 2 are sent to the ST-HSC pool. Signals 2 and 3 to the LT-HSC pool. HSC pool exit occurs following a circadian rhythm where the frequency goes from 0 to double over the day. A control with no circadian rhythm was run in each case: no significant differences were observed. All runs were repeated 256 times, which was enough to reduce the standard error of all tracked quantities to 1% at most.

The model is based on the following assumptions and experimentally measured parameters: 1) The populations considered are hierarchically organized in the following order: LT-HSC, ST-HSC, progenitors. 2) Initially these populations contain a fixed number of cells at a ratio of 1:1:1000 for LT-HSC:ST-HSC:Progenitors which represents the experimental measure by flow cytometry and limiting dilution assays (data not shown). The simulations were run with initial populations set at 500 LT-HSCs, 500 ST-HSCs and 50000 Progenitors, which are also defined to be the target numbers to be maintained for homeostasis. 3) Each cell within the LT- and ST- HSC compartments is modeled to be in G_0, G_0 exit, or a cycling state. 4) Signals to divide or not divide are generated from the difference between the number of cells in a pool and the target number. 5) LT and ST HSCs always go back to G_0 when they divide. 6) There is no symmetrical division that produces 2 differentiated cells. 7) HSC death rate was defined by AnnexinV experimentally at

5% (data not shown). 8) There is noise in the system (the noise function is generated as a uniformly distributed random integer). The number of cells receiving the signal to exit G_0 is thus equal to the number of missing cells plus a randomly distributed uniform number representing noise, all other cells remain quiescent.

There are 2 parameters that cannot be measured experimentally and whose value may affect the outcomes of the simulations: 1) the HSC pool exit rate: this parameter determines how many cells get a signal to divide 2) different levels of noise. Therefore, in this implementation we tested all range of possible HSC pool exit rates: from 0 to 100% of compartment (Fig. S6B-E) as well as all reasonable noise values (Fig. S6F-G), to ensure our conclusions are robust. For values of HSC pool exit above 128 cells/day (25% of the pool), the number of progenitors cannot be maintained. By comparison, given our measured cycle times, a pool of 500 cells could give rise to a maximum of 177 cells/days if they divided continuously. The noise simulates variability in the mitogenic signals received. Indeed, it is unlikely that the number of missing cells be known to the unit, and, on the other hand, a signal containing no information cannot be used in a control loop.

There are 2 parameters that arise for any given HSC exit rate and noise value: 1) the self-renewal/differentiation ratio and 2) the number of divisions/year. This last parameter has been estimated experimentally in humans so we have chosen the combination of HSC pool exit rate (24 cells/day) and noise (5%) that outputs the number of divisions per year closest to the range reported in the literature (Catlin et al., Blood, 2011).

Two experiments were conducted: one at steady-state (homeostasis) and one where a perturbation is applied to simulate a stress/injury situation. The outcomes of maintaining a 5.8 hours G_0 exit delay in LT-HSCs compared to ST-HSCs (LT-HSC t_{G0exit} delay = 5.8h) were compared to a situation where there is no difference in G_0 exit duration between LT- and ST-HSCs (LT-HSC t_{G0exit} delay = 5.8h), or a situation in which HSCs are governed by a unique kinetic parameter (Fig.6A). The perturbation consisted in killing 10% of the progenitors at once, which subsequently requires all ST-HSCs to divide at least 10 times to return to the steady-state situation. As discussed above, simulations were run with 5 distinct HSC pool exit rates and 6 different noise parameters and all converged to the same conclusions. Five robust findings emerge. First, while the choice of the HSC pool exit rate and noise parameters affects the absolute values of the self-renewal/differentiation ratios, these are always higher for LT-HSCs, thus corroborating experimental data attributing the highest self-renewal capacity to LT-HSCs. This ratio is largely not influenced by the existence of a delay in G_0 exit in LT-HSCs (Fig.S6B-C). Second, in all the combinations of parameters tested, and maintaining a cell cycle transit time of LT-HSCs longer than that of ST-HSCs, the number of divisions in LT-HSCs was always minimized when LT-HSCs were set to have a delayed G_0 exit than ST-HSCs (Fig. 6B-C, Fig.S6D-G). This is an important property for the HSC pool as it is believed that LT-HSCs form a reservoir of pristine cells and each division is likely to accumulate damage. Third, we verified the dynamic range in which longer LT-HSC quiescence exit times may minimize LT-HSC divisions. Even if the LT-HSC G_0 exit delay is halved (2.9 hours), the number of LT-HSC divisions is still significantly lower than when quiescence exit times are not differentially maintained in LT- and ST-HSCs. Fourth, the progenitor population is maintained with similar efficiency

independent of whether the LT-HSCs cells have a longer t_{G0exit} than ST-HSCs (Fig.S6H). Fifth, after perturbation, the return to steady-state is faster when the LT-HSCs are delayed in their G_0 exit (Fig. 6H, Fig.S6H-I). In the case in which there is no delay in G_0 exit times in LT-HSC compared to ST-HSC, spikes in the number of cycling cells mean that the system at that point is less responsive to further perturbations (Fig.S6I). This last finding indicates that the control of the number of downstream cells is overall more robust when the G_0 exit times differ between LT- and ST-HSCs. Collectively, additional differential control of the delay in G_0 exit of LT-HSCs further limits LT-HSC division and increases the overall robustness of the system. Differential regulation of G_0 exit duration in the HSC hierarchy is thus key to maintain the integrity of the HSC pool.

Supplemental References

Amendola, M., Venneri, M.A., Biffi, A., Vigna, E., and Naldini, L. (2005). Coordinate dual-gene transgenesis by lentiviral vectors carrying synthetic bidirectional promoters. Nat. Biotechnol. *23*, 108–116.

April, C., Klotzle, B., Royce, T., Wickham-Garcia, E., Boyaniwsky, T., Izzo, J., Cox, D., Jones, W., Rubio, R., Holton, K., et al. (2009). Whole-genome gene expression profiling of formalin-fixed, paraffin-embedded tissue samples. PloS One *4*, e8162.

Aryee, M.J., Gutiérrez-Pabello, J.A., Kramnik, I., Maiti, T., and Quackenbush, J. (2009). An improved empirical bayes approach to estimating differential gene expression in microarray time-course data: BETR (Bayesian Estimation of Temporal Regulation). BMC Bioinformatics *10*, 409.

Catlin, S.N., Busque, L., Gale, R.E., Guttorp, P., and Abkowitz, J.L. (2011). The replication rate of human hematopoietic stem cells in vivo. Blood *117*, 4460–4466.

Ernst, J., Nau, G.J., and Bar-Joseph, Z. (2005). Clustering short time series gene expression data. Bioinforma. Oxf. Engl. *21 Suppl 1*, i159–i168.

Fan, J.-B., Chen, J., April, C.S., Fisher, J.S., Klotzle, B., Bibikova, M., Kaper, F., Ronaghi, M., Linnarsson, S., Ota, T., et al. (2012). Highly Parallel Genome-Wide Expression Analysis of Single Mammalian Cells. PLoS ONE *7*, e30794.

Van Galen, P., Kreso, A., Wienholds, E., Laurenti, E., Eppert, K., Lechman, E.R., Mbong, N., Hermans, K., Dobson, S., April, C., et al. (2014). Reduced Lymphoid

Lineage Priming Promotes Human Hematopoietic Stem Cell Expansion. Cell Stem Cell *14*, 94–106.

Mazurier, F., Gan, O.I., McKenzie, J.L., Doedens, M., and Dick, J.E. (2004). Lentivector-mediated clonal tracking reveals intrinsic heterogeneity in the human hematopoietic stem cell compartment and culture-induced stem cell impairment. Blood *103*, 545–552.
